# YTH Domain Proteins Play an Essential Role in Rice Growth and Stress Response

**DOI:** 10.3390/plants11172206

**Published:** 2022-08-25

**Authors:** Weiwei Ma, Song Cui, Zhenfei Lu, Xiaofeng Yan, Long Cai, Yongfa Lu, Kefeng Cai, Huacheng Zhou, Rongrong Ma, Shirong Zhou, Xiaole Wang

**Affiliations:** 1 Institute of Crop Sciences, Ningbo Academy of Agricultural Sciences, Ningbo 315000, China; 2 State Key Laboratory for Crop Genetics and Germplasm Enhancement, Jiangsu Plant Gene Engineering Research Center, Nanjing Agricultural University, Nanjing 210095, China

**Keywords:** YTH domain protein, CRISPR, rice, growth, stress response

## Abstract

As the most prevalent epi-transcriptional modification, m^6^A modifications play essential roles in regulating RNA fate. The molecular functions of YTH521-B homology (YTH) domain proteins, the most known READER proteins of m^6^A modifications, have been well-studied in animals. Although plants contain more YTH domain proteins than other eukaryotes, little is known about their biological importance. In dicot species Arabidopsis thaliana, the YTHDFA clade members ECT2/3/4 and CPSF30-L are well-studied and important for cell proliferation, plant organogenesis, and nitrate transport. More emphasis is needed on the biological functions of plant YTH proteins, especially monocot YTHs. Here we presented a detailed phylogenetic relationship of eukaryotic YTH proteins and clustered plant YTHDFC clade into three subclades. To determine the importance of monocot YTH proteins, YTH knockout mutants and RNAi-induced knockdown plants were constructed and used for phenotyping, transcriptomic analysis, and stress treatments. Knocking out or knocking down *OsYTHs* led to the downregulation of multicellular organismal regulation genes and resulted in growth defects. In addition, loss-of-function *ythdfa* mutants led to better salinity tolerance whereas *ythdfc* mutants were more sensitive to abiotic stress. Overall, our study establishes the functional relevance of rice *YTH* genes in plant growth regulation and stress response.

## 1. Introduction

In the past 30 years, epigenomic modifications (DNA modifications, RNA modifications, and histone modifications) have been widely studied and shown to play essential roles in regulating gene expression, stress resistance, development, and other key biological processes [1,2,3,4]. Current studies demonstrate that the epi-transcriptional modifications, especially the most prevalent m^6^A modification, exist widely in eukaryotic RNAs and function in regulating RNA fate, such as alternative splicing, RNA export, 3′ untranslated region (UTR) processing, and RNA stability [5]. In plants, m^6^A modification is an essential and extensive molecular mechanism in regulating organ development [6,7,8,9,10,11,12,13,14], circadian rhythm [15], fruit ripening [16], and stress tolerance [11,12,17,18,19,20]. A recent study also revealed that FTO-mediated m^6^A demethylation in rice caused a more than threefold increase in grain yield [21]. As a critical post-transcriptional regulatory pathway, dynamic reversible m^6^A modification after RNA transcription is an exciting and efficient new way to regulate gene expression, which provides a new approach to understanding epigenetic regulation in plants and further improves agronomic traits in plant breeding.

As the most known READER proteins of m^6^A modifications, YT521-B homology (YTH) domain proteins are also found in most eukaryotic species and are well-studied, especially in vertebrates [5]. Three categories can be classified in animal YTH proteins, namely the YTHDF (YTH domain-containing family protein) family, YTHDC1 (YTH domain-containing protein 1, also called DC1), and YTHDC2 (YTH domain-containing protein 2, also called DC2). Most vertebrates have three YTHDF members, whereas only one YTHDF protein was found in invertebrate species. By influencing RNA stability, alternative splicing, or translation, YTHDFs mainly take part in stem cell differentiation/cancer progression [22,23,24] and neuronal function [25,26,27,28,29,30,31]. In contrast, YTHDC1 and YTHDC2 function in sex determination by affecting the alternative splicing and 3′ untranslated region (UTR) processing [28,29,30,32,33,34,35,36,37,38].

Compared to the restricted members of YTH proteins in animals, plants contain much more YTH proteins, whereas little is known about their biological importance. In Arabidopsis, the ECT2/3/4 are well-studied and important for cell proliferation and plant organogenesis. ECT2 forms a complex in vivo with m^6^A RNA via its YTH domain, whereas plants depleted of ECT2 show a trichome branching defect [12]. Weaker trichome branching defects can also be observed in ect3 single mutants, whereas ect2/ect3 double mutants exhibit more severe trichome defects and delayed post-embryonic leaf formation [13]. By additional mutation of ECT4, ect2/ect3/ect4 triple mutants have exacerbated the slow growth of leaf primordia and serrated-edged leaves, which resembles that of mta knockdown plants [39]. Recently, Arribas-Hernández, Rennie et al., reveal the mode of RNA recognition by YTH domain-containing proteins (ECT2/3) using HyperTRIBE which coheres the genetic and molecular studies of m^6^A-YTH function [20,40]. Other than YTHDF proteins, AtCPSF30L, as one of the YTHDC members, modulates nitrate content by regulating nitrate transport and assimilation in plants [41]. This raises the question, what are the functions of the rest of plant YTH proteins? Why does the plant need so many YTH proteins? 

To answer these questions, we present our research on YTH proteins in a monocot model plant—rice. In this research, a detailed phylogenetic relationship of eukaryotic YTH proteins was solved, and a total of five subclades could be found within the plant YTHDF clade, and no YTHDC2 was found in plant species. To determine the importance of rice YTH proteins, YTH knockout mutants and RNAi-induced knockdown plants were constructed. Agricultural trait changes were observed within these transgenic plants, and transcriptomic data supported that the expression level of multicellular organismal regulation genes has changed. In addition, transcriptomic data showed that most mutants enriched stimulus response genes, indicating that *OsYTH* genes may also contribute to a stress response which was further supported by a stress tolerance assay. In conclusion, we hypothesize that the large number of YTH proteins in vascular plant species participated in balancing self-growth and adaptation to variant growth environmental changes that plants cannot avoid.

## 2. Results

### 2.1. Phylogenetic Analysis of YTH Proteins

Previous studies have conducted much work on the phylogenetic relationship of YTH family proteins, but either these works focused only on a specific group of species or YTH proteins in which the researchers are interested [42,43]. In this research, 561 YTH domain-containing proteins (Supplementary Tables S1–S3) from 37 plant species, 47 animal species, and two yeasts were collected, covering most eukaryotic categories. YTH domains were used for phylogenetic tree construction.

**Figure 1:**
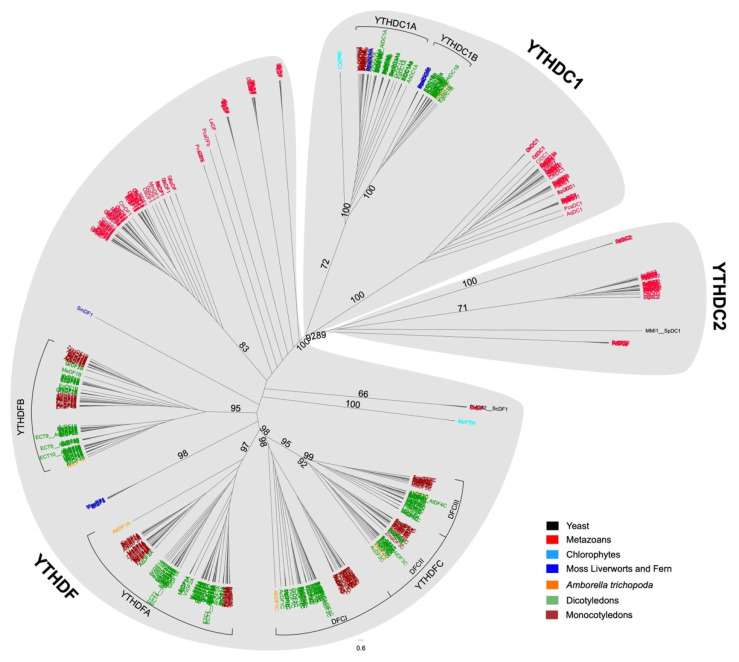
Phylogenetic tree of YTH domains from animal and plant YTH proteins using maximum likelihood method. Phylogenetic analysis of the eukaryotic YTH family. An unrooted maximum likelihood tree was constructed with iqtree focusing on major model species representing a wide taxonomic range. Numbers on branches indicate the percentage of trees in which associated taxa clustered together during bootstrap analysis (1000 bootstraps). Only the values on the main clades are shown.

In coincidence with previous studies on animal YTH proteins, the phylogenetic tree of YTH proteins can be classified into three categories (Figure 1), which are YTHDC1, YTHDC2, and YTHDF family, representing the three categories described in animals [6]. All the plant YTHDC proteins are more closely related to YTHDC1 proteins, and no YTHDC2 homolog was found in plant species (Figure 1). Besides, the plant YTHDF proteins are paralogs to animal YTHDF proteins. The YTHDF genes of non-vascular plant species such as algae, liverworts, or mosses formed the outgroup of all plant YTHDF proteins whereas vascular plant YTHDF genes formed three well-supported clades YTHDFA/B/C (Support value = 95, 97, 98, respectively) as the previous study suggested [12]. Furthermore, by adding the basal angiosperm species *Amborella trichopoda* into the construction of the phylogenetic tree, three subclades can be recognized in the YTHDFC clade and named DFCI (including OsYTHDF2C, OsYTHDF3C, ECT6, and ECT7), DFCII (including OsYTHDF1C, and ECT8), DFCIII (including OsYTHDF4C, OsYTHDF5C, and ECT11) with high support value (98, 92, 99, respectively) (Figure 1). Each YTHDFC subclade contains one AtrYTHDFC protein and 0–4 YTHDFC proteins from other angiosperm species (Supplementary Table S1).

Unlike the conserved YTHDC1 genes in all species collected in our research (most animal species have only one copy of YTHDC1), plant YTHDC1 can be divided into two subclades, DC1A and DC1B (Figure 1). Moss, liverworts, fern, and most dicots have both DC1A and DC1B proteins, whereas monocot plants have only DC1A protein, which indicates that a loss event has occurred in the common ancestor of modern monocot species. Other than YTHDC1 proteins, the number of YTHDFs also varied from species to species (Supplementary Tables S1 and S2). Most non-vascular or non-vertebrate species have only limited YTHDF genes (usually 1–2), whereas vascular or vertebrate species have more. Within Chondrichthyes and vertebrate animals, exactly three YTHDF genes can be recognized. The only exception is in species from Salmoniformes and Neoteleostei families, which have an additional copy of the YTHDF1 gene (Figure 1 and Supplementary Table S2). In contrast, vascular plants have more YTHDF genes, which usually vary from 4 to 20 (Supplementary Tables S1 and S2). 

Numerous works have been conducted to reveal the molecular function of animal YTHs, whereas a few focused on Arabidopsis YTH proteins (ECT2/3/4 and AtCPSF30L). To obtain an initial understanding of the functions of YTH domain proteins in monocot plants, rice YTH proteins were used for further study.

### 2.2. Expression Analysis of the OsYTH Genes in Different Tissues/Organs

**Figure 2:**
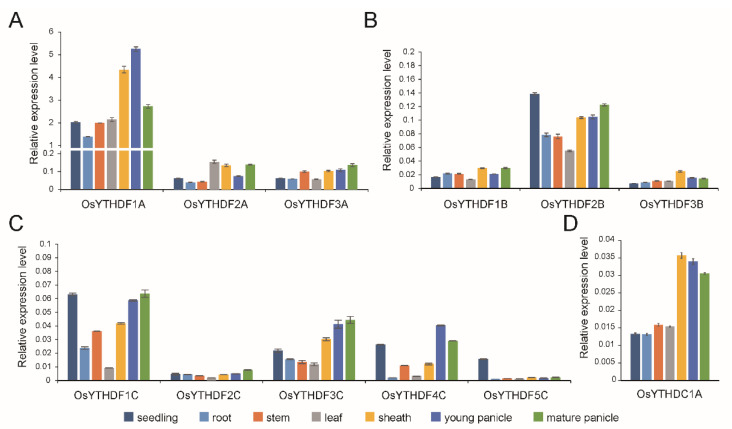
The tissue expression patterns of the *YTHDFA* (**A**), *YTHDFB* (**B**), and *YTHDFC* (**C**) members and *YTHDC1A* (**D**) in different tissues of rice. Different colors denoted different tissues. Values are means ± SD of three independent biological replicates.

Seven tissues/organs (young seedling, root, stem, leaf, sheath, young panicle, and matured panicle) at different development stages were used for tissue expression pattern analysis. The result showed that most *OsYTHs* are ubiquitously expressed across plant development, except for *OsYTHDF5C*, which is merely expressed in tissues other than young seedlings (Figure 2). Among the tissues tested, the *OsYTHs* showed relatively higher expression levels in sheath and panicles than other tissues. *OsYTHDF1A* showed the highest expression level of all the *YTH* genes (Figure 2A). The other two *YTHDFA* members also showed higher expression levels than *YTHDFB* and *YTHDFC* genes (Figure 2). These results are consistent with the previous study that *ECT2* and *ECT3* are the most highly and widely expressed members of the YTH family in *Arabidopsis* [13]. The rest of the *YTH* members showed relatively lower expression levels, with *OsYTHDF2B* and *OsYTHDF1C* performing the highest expression level in each clade (Figure 2B–D).

### 2.3. Characterization of Loss-of-Function Mutants and Knockdown Plants

To learn the function of YTH proteins in rice, the CRISPR-Cas9 system was used to generate loss-of-function mutations. Except for *OsYTHDF2C* and *OsYTHDC1A*, ten loss-of-function mutants with either an insertion or deletion of one or few bases at the target sequences which caused early termination were successfully gained and confirmed by sequencing (Supplementary Figures S1–S4). For *OsYTHDF2C*, although several lines carrying a loss-of-function mutation were detected in T0 generation and seeds were collected (Supplementary Figures S1C and S4B), all the T1 seeds failed to germinate. On the other hand, osythdc1a mutations were detected in multiple transgenic lines, but all the mutations did not lead to frameshift changes (Supplementary Figures S1D and S4F). These results indicate that OsYTHDF2C and OsYTHDC1A might be very important for plant development, whose loss-of-function mutation will be lethal for plants. For these two genes, three lines of siRNA-induced knockdown plants were carried out for each gene whose expression level has been confirmed by RT–PCR results (Supplementary Figure S5) and used for further studies. 

**Figure 3:**
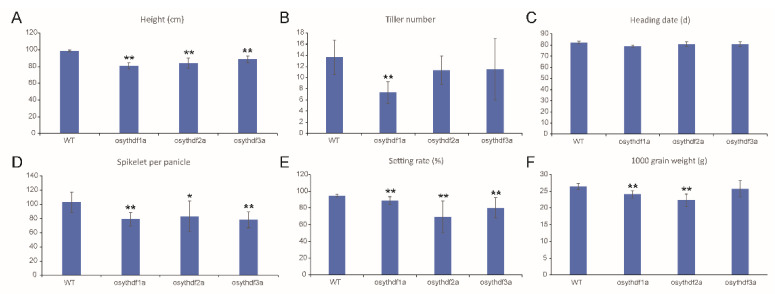
Quantification of several agricultural traits of *DFA* clade mutants including plant height (**A**), tiller number (**B**), heading date (**C**), spikelets number per panicle (**D**), setting rate (**E**), and 1000 grain weight (**F**). Values are shown as mean ± SD. The significance of difference was determined by Student’s *t*-test (*, *p* < 0.05; **, *p* < 0.01).

In all the mutants/knockdown plants, agricultural trait changes have been observed (Figure 3 and Supplementary Figure S6). Most mutants/knockdown plants showed decreases in plant height, the number of spikelets per panicle, setting rate, and grain weight (Figure 3A,D–F and Supplementary Figures S6A,D–F), whereas the heading date did not change too much in either mutation or knockdown plants (Figure 3C and Supplementary Figure S6C). Loss-of-function of *YTHDFA* clade protein led to a significantly decreased tiller number whereas other mutants/knockdown plants performed conversely (Figure 3B and Supplementary Figure S6B). These results indicated that the rice YTH proteins are essential for shaping the plant architecture.

### 2.4. OsYTHs Are Essential for Plant Growth

**Figure 4:**
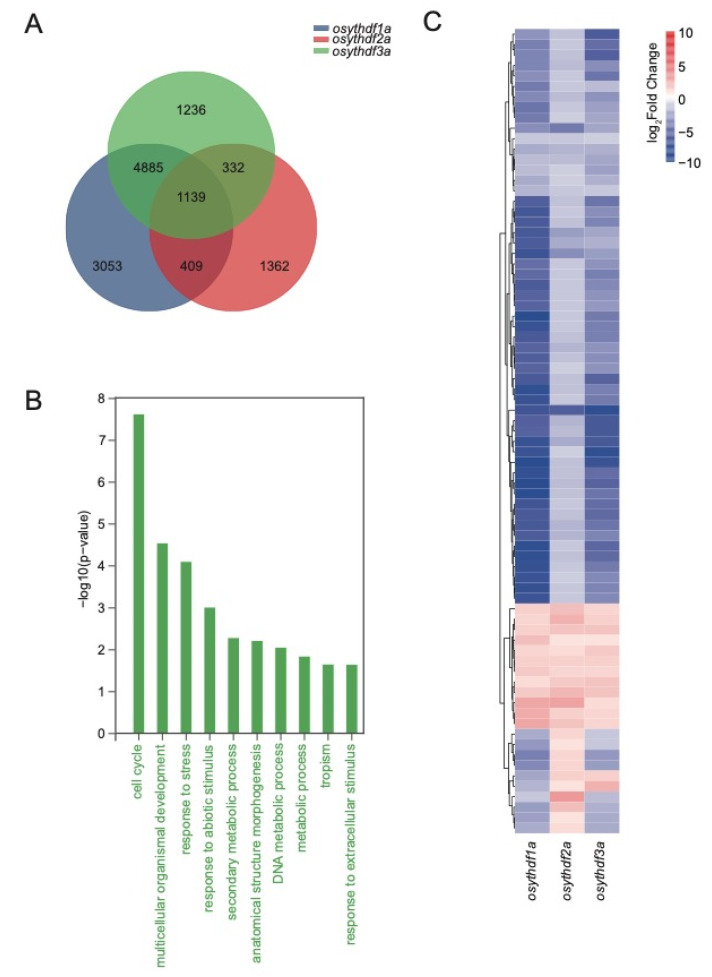
The Venn diagram, GO enrichment analysis, and heatmap of DEGs in YTHDFA clade mutants. (**A**) The Venn diagram showing unique and shared DEGs in YTHDFA mutants. (**B**) The GO enrichment analysis of 1139 DEGs shared within YTHDFA clade. (**C**) Expression heatmap of 77 organismal development-related genes. Heatmap was drawn according to log_2_FC values, which were calculated pairwise based on the expression level of each mutant and wild-type plant.

Transcriptomic analyses were carried out for a deeper view of “how does lack of YTH proteins interference with the plant”. Thousands of significantly differentially expressed genes (DEGs) were found within *yth* mutants or knockdown plants compared to the wildtype transcriptomic data (Figure 4A and Supplementary Figure S7). 

Within the *YTHDFA* clade, which showed the most significant agricultural trait changes, 1139 different expression genes (DEGs) were shared by all three *ythdfa* mutants (Figure 4A), with the multicellular organismal development process enriched by GO analysis (Figure 4B). A total of 77 organismal development-related genes exhibited expression changes in *YTHDFA* clade mutants, out of which 65 genes were significantly decreased, including *OsYABBY5* [44], *OsCD1* [45,46], *OsAPO2* [47,48], *OsARF5* [49], *OsAGO1c* [50], *SD1* [51], *OsAS2* [52], whose mutants/knockdown plants were previously reported and showed developmental defects (Figure 4C and Supplementary Table S4). Other than *YTHDFA* members, the multicellular organismal development process was also enriched in *osythdf4c* and *RNAi-OsYTHDC1A* plants (Supplementary Figure S8). In combination with the mutation phenotype of these genes, these results indicate that rice YTH domain-containing proteins, mainly *YTHDFA* clade members and *OsYTHDC1A*, affect plant architecture by regulating the expression of organismal development-related genes.

### 2.5. YTH Proteins Are Required for Stress Response

**Figure 5:**
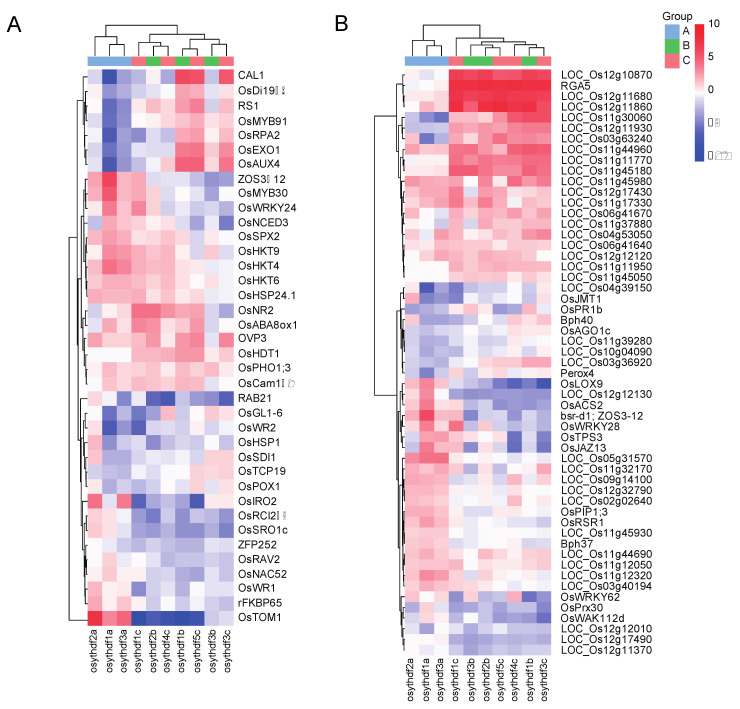
Expression heatmap of 38 abiotic response genes (**A**) and 55 biotic response genes (**B**) which have been previously reported. Heatmap was drawn according to log2FC values, which were calculated pairwise based on the expression level of each mutant and wild-type plant.

Previous studies have proven that m^6^A modifications and ECT2 take part in stress response [11,12,17,18,19]. Interestingly, “response to biotic stimulus” and “response to abiotic stimulus” were also commonly enriched by GO analysis in most mutants/knockdown plants, except for *osythdf3a* (Supplementary Figure S8), indicating that the rice YTH proteins also take part in stress response progresses. Among the DEGs related to “response to biotic stimulus” and “response to abiotic stimulus”, 38 abiotic response genes (including response to drought, salt, cold, heat, ABA, Ion deficiency, and UV damage), 17 biotic response genes (including response to rice blast, bacterial blight, sheath blight, rice dwarf, planthopper, etc.) and 38 LRR disease resistance genes were recognized. Heat map clustering showed that the mutants that carried YTHDFA clade mutations have a similar pattern in both biotic and abiotic stress response genes. In contrast, mutants that carried YTHDFB or YTHDFC clade mutations gathered into another clade (Figure 5, Supplementary Tables S6 and S7) (only mutants were used for heat map analysis due to relatively low expression changes in knockdown plants). Around half of the abiotic stress response genes (17 out of 38) and a third of the biotic stress response genes (20 out of 55) showed an opposite expression change from ythdfa clade mutants to others (Figure 5). A total of 21 LRR genes were significantly upregulated with three downregulated in ythdfb and ythdfc mutants whereas the ythdfa mutants did not show significant changes (Figure 5B). 

As the transcriptomic result reveals that the OsYTHs take part in abiotic stress responses, four abiotic stress response patterns were tested in a cultivated variety of Nipponbare, including salt, drought/PEG-induced osmotic stress, and cold. As ABA plays a vital role in the stress response and tolerance of plants, the ABA response was also determined. Most *OsYTH* genes significantly upregulated after abiotic stress treatment for 3–6 h, whereas some *OsYTHs* responded later (Supplementary Figure S9). In contrast, most YTH genes didn’t show significant expression changes under ABA treatment. These results indicated that the *OsYTH* genes take part in stress response and might not through the ABA signal pathway. 

**Figure 6:**
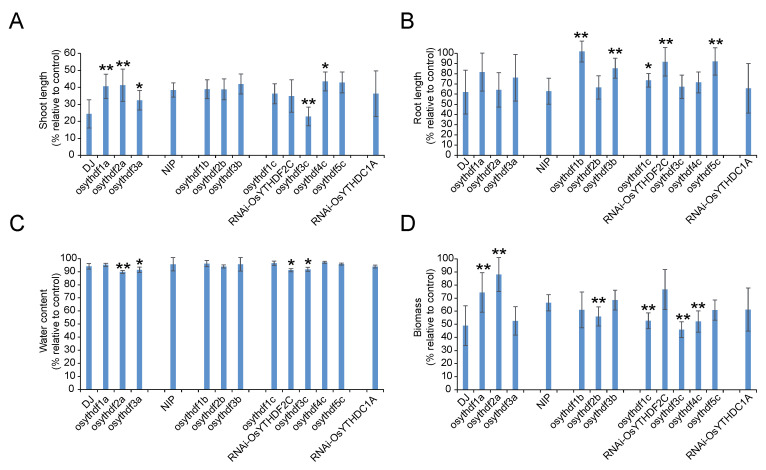
Phenotypic evaluation of YTH mutants/knockouts under salt stress. The shoot length (**A**), root length (**B**), water content (**C**), and biomass (**D**) values correspond to a % of the change in salt relative to control. Values are shown as mean ± SD. The significance of the difference was determined by Student’s *t*-test (*, *p* < 0.05; **, *p* < 0.01).

To further investigate the connection between YTH proteins and phenotypes under abiotic stress, loss-of-function mutants and knockdown plants were treated with 100 mM/150 mM NaCl as described in the methods section. At the end of the 100 mM NaCl treatment, all plants showed complete growth retardation with shorter shoots/roots, lower water content, and lower biomass (Figure 6). In comparison to the WT (DJ for *YTHDFA* clade mutants and NIP for *YTHDFB/YTHDFC* clade mutants and *RNAi-OsYTHDC1A*), all the *ythdfa* mutants showed significantly lower growth retardation on shoot/root length and biomass, whereas the *ythdfb* and *ythdfc* mutants showed marked reduction (Figure 6A,B,D). The percentage of water content was not substantially different between WT and most mutants (Figure 6C). In order to determine the survival rate under salt stress, 150 mM NaCl was applied to all the wild-type plants and mutants with different treatment times (7 days for assays 1 and 2, 8 days for assay 3). Most *ythdfc* mutants and *RNAi-OsYTHDC1A* plants showed lower survival rates than the wild-type for all the three assays, whereas *ythdfb* mutants remained similar to NIP (Supplementary Figure S10A). The *ythdfa* mutants did not show significant changes under the 7-day treatment, but the survival rates of *osythdf2a* and *osythdf3a* were significantly higher when the salt treatment time was extended to 8 days (Supplementary Figure S10A,B). In conclusion, the loss of *ythdfa* genes results in better tolerance to salinity treatment, with *ythdfc* and *ythdc1a* mutants being more sensitive to salt stress. These results indicated that apart from regulating plant growth, which is mainly related to the function of YTHDFA clade members and YTHDC1A, OsYTHs also contribute to stress response progresses.

## 3. Discussion

In this research, we constructed a fine phylogenetic tree of YTH proteins covering most of the eukaryotic categories, which helped us to solve the relationship between animal YTH proteins and plant YTH proteins, clustered the plant YTH proteins, and further raised our interest in the importance of numerous YTH proteins in plants, especially monocot plants. Expression analysis showed that most *YTH* genes have ubiquitous expression patterns with the exception of *OsYTHDF5C*. The transcriptomic analysis of knockout mutants and knockdown plants revealed that *OsYTH* genes might take part in growth regulation and stress responses progress. The in-field phenotype proved that the loss of *YTHDFA* clade members and *OsYTHDC1A* would lead to defects in plant architecture development. Other than growth regulation, the *OsYTHs* also contribute to stress response progress which was proved by stress response analysis and salinity tolerance assay. 

One of the main functions of animal YTHs is to regulate stem cell differentiation/cancer progression [22,23,24]. *Arabidopsis* ECT2/3/4 are also reported to be important for organogenesis and cell proliferation through binding to the m^6^A site and affecting mRNA stability [6,12,13,53,54]. Single mutants in *AtYTH* genes do not exhibit obvious developmental phenotypes other than abnormal trichome branching, and double or triple mutations in *ECT2*/*ECT3*/*ECT4* lead to delayed growth and aberrant morphology [6,12,13,53]. In this study, most single mutants of *OsYTHs* showed multiple growth defects, including decreased plant height, changed tiller number, and smaller panicles, showing that rice YTH proteins play similar roles in regulating plant architecture. The transcriptomic results also supported that the knockout of rice *YTHDFA* clade members, *OsYTHDF4C*, and knockdown of *OsYTHDC1A*, will lead to expression changes of multicellular organismal development-related genes. Notably, the heading date did not change in both mutation and knockdown plants, indicating that the rice YTH proteins do not take part in regulating the timing of flowering. In addition, we also found that *OsYTHDF2C* and *OsYTHDC1A* loss-of-function mutations are lethal for plants. Previous studies have also proved that *ect2*/*ect3*/*ect4* triple mutations slow down the growth of leaf primordia, but do not affect the initiation timing of leaf primordia [53]. These results indicate that the m^6^A -YTH regulatory module plays a vital role in plant organogenesis by regulating the expression level of organogenesis-related genes, especially in monocot species. 

Qian and his team member have reported that the nuclear YTHDF2 preserves 5′UTR methylation of stress-induced transcripts under heat stress, further promoting cap-independent translation initiation [55]. Aside from the response to abiotic stress, m^6^A modification and YTHDF2 also act as critical regulators of immune cell homeostasis by mediating the gene expression of immune-related signaling pathways [56,57]. In plants, stress response genes were upregulated in ect2/3/4 mutants, although possibly through indirect effects [20], and the expression pattern of *YTH* genes can also be induced by biotic and abiotic stress [58,59]. In this study, we found that not only do the expression levels of *OsYTHs* respond to stress stimulus but also that the *OsYTHs* take part in a stress response by regulating the expression of stress response genes. Transcriptomic results showed that the expression levels of numbers of the stress response genes were changed in most *osyth* mutants. NaCl treatment also demonstrated that *OsYTHs* are important for abiotic stress resistance, especially for YTHDFA clade and YTHDFC clade members. Aside from abiotic stress, biotic stimulus response genes were also enriched in transcriptomic results. The function of OsYTHs in biotic stress remains further studied. In addition, the extra YTHDF1-like gene found in five species of Salmoniformes and Neoteleostei (Supplementary Table S2) which usually lived in harsh environments (such as highly variable salinity, temperature fluctuations, low oxygen levels, and heavily polluted ecosystems) also reminds us that this additional YTHDF1-like gene might contribute to their adaptation to different environments. After all, these findings indicate that YTH domain-containing proteins and m^6^A modifications might be related to the adaptability to the environment not only in animals but also in plants. 

With the help of the phylogenetic tree we constructed in this study, we can discover that the expansion event mainly occurred after the initiation of land plants, which have more complicated organs (leaf, stem, roots, flowers, and fruits) and live in more complicated environments (Figure 1). Transcriptomic analysis and phenotype evidence, together with the previous study on ECT2/3/4 [6,13,14,54], indicated that the land plant YTHDFA clade members contributed to the organogenesis progress. In addition, knocking out YTHDFA clade members results in growth defects and increased tolerance to abiotic stress, whereas knocking out YTHDFC clade members (especially YTHDFCI and YTHDFCII subclade members) results in more tiller number and more sensitivity to abiotic treatments. Taken together, we hypothesized that the numerous numbers of plant YTH proteins balanced the development of multicellular organisms and their responses to environmental stimuli, which allowed the land plants to generate various tissue types and adapt to different living environments.

Numerous studies have shown that m^6^A and YTH domain-containing proteins play important roles in animals, including cell differentiation [22,23,24], learning, memory, and fitness to environment [25,26,27,28,29,30,31]. It attracts attention to the study of m^6^A and its readers in plants. In this study, we mainly focused on the phenotype and transcriptomic changes (especially organogenesis and stress response) of loss-of-function mutants and knockdown plants and showed that the plant YTH proteins are not only crucial for plant architecture but also take part in stress response, which, given an initial work on the function of YTH domain-containing proteins in rice, provides an advancement in the further studies of the biological functions of plant YTH proteins. Substantial work is left to be conducted on screening out the target genes of YTH proteins and exploring the molecular mechanisms of YTH-mediated post-transcriptional regulating systems.

## 4. Materials and Methods

### 4.1. Sequence Selection, Multiple Sequence Alignments, and Phylogenetic Reconstruction

BLAST searches (blastp) were performed starting from known Human and Arabidopsis YTH domains on 86 species representing the diversity of the Eukaryote lineage at the Uniprot (https://www.uniprot.org/blast/ (accessed on 18 August 2022)), NCBI (https://www.ncbi.nlm.nih.gov/ (accessed on 18 August 2022)), and Phytozome 13 (https://phytozome-next.jgi.doe.gov/ (accessed on 1 November to 15 December 2018)). Each time a new YTH was found in a given species, it was used as a query in a new BLAST search until no new YTH gene was found. Sequences annotated as “partial sequence” or “low-quality sequence” were discarded, and other YTH sequences were aligned using the MUSCLE v3.8.425 [60] on Geneious v2021 software using standard parameters. Maximum likelihood (ML) trees were reconstructed using the iqtree web server [61] (http://iqtree.cibiv.univie.ac.at/ (accessed on 18 August 2022)) with the JTT + I+G4 amino acids replacement matrix. Main branch support values were estimated from bootstrap analyses of 1000 replicates. All manipulations on phylogenetic trees were performed with Figtree (v1.3.1).

### 4.2. Growth Conditions and Abiotic Stress Treatment

*Oryza sativa* sub. Japonica var. Nipponbera (NIP) and Dongjin (DJ) were used as the wild-type ecotypes. Transgenic and wild-type seeds were soaked in water for germination at 37 °C for three days and then grown in a paddy field in Nanjing. For tissue expression pattern analysis and transcriptomic analysis, wild-type and transgenic plants were grown in climate chambers (BES1500QH-LED, BOERSI) after germination with 70% humidity and 14 h light/10 h dark photoperiod for two weeks before sampling. For determination of YTH gene expression response pattern to abiotic stress, 14-day-old NIP seedlings were grown in nutrient solution supplemented with 150 mM NaCl/100 mM ABA/20% PEG4000 for salt/ABA/PEG treatment for previously determined time, respectively. Cold treatment was conducted under 5 °C. For determining the salinity tolerance, germinated seeds were grown in the nutrient solution with or without (mock) 100 mM NaCl under the same growth condition at 25 °C under 14 h light/10 h dark in a growth room. The plant height, root length, water content, and biomass were measured after 14 days. For determining the survival rates under salt stress, 14-day-old seedlings were subjected to the nutrient solution containing 150 mM NaCl for seven days (for assays 1 and 2) or eight days (for assay 3) at 25 °C under 14 h light/10 h dark in a growth room and subsequently grown in the nutrient solution for recovery. The survival rates were calculated after recovery for seven days. 

### 4.3. Vector Construction and Rice Transformation

To knock out the OsYTH genes, 18-bp gene-specific spacer sequences of each target gene were cloned into the entry vector pCRAC harboring both Cas9 and sgRNA segments. Mutation type was determined by PCR and sequencing. 

To knock down OsYTHDF2C and OsYTHDC1A, both anti-sense and sense versions of specific 416 bp or 418 bp fragments from the coding region of either gene were amplified and successively inserted into the SacI and SnaBI restriction site of the LH-FAD1390RNAi binary vector to form the RNAi construct. 

All the resulting plasmids were transformed into rice variety Nipponbera (NIP, as transgenic receptor for ythdfb/ythdfc/ythdc1a knockout/knockdown plants) and Dongjin (DJ, as transgenic receptor for ythdfa knockout mutants) by Agrobacterium-mediated transformation and subsequently confirmed by PCR sequencing. Primer sequences for vector constructions and sequencing are listed in Supplementary Table S5.

### 4.4. Measurement of Morphological Traits

For measuring the plant height, tiller number, and heading date of each loss-of-function mutant/knockdown, 6 individuals were selected and measured manually in the field. For panicle traits, at least 9 panicles from 3 individuals were selected and dried at 50 °C for 5 days. The number of empty grains was counted manually and the number of filled grains and 1000-grain weight were measured by SC-G automatic seeds test and thousand kernels weightier (WANSHEN). The setting rate was calculated by numbers of filled grains/numbers of total grains.

For the salinity-tolerant assay, the shoot length (cm) was measured from the base of the stem to the tip of the topmost leaf of the plants. Root length (cm) was measured for each plant. Fresh weight (FW) was determined immediately after collecting the samples, followed by tissue drying at 50 °C for 7 days for the determination of dry weight (DW). Water content was estimated as follows %WC = (FW-DW)/DW) × 100 and biomass refers to the dry weight. All parameters are presented as percentages relative to control plants.

### 4.5. Total RNA Isolation and RT–PCR Analysis

Young seedlings were collected 14 days after germination and leaf blades, sheath, stems, and panicles were collected 60 days after germination. Total RNA were isolated from frozen tissues using a ZR Plant RNA MiniPrep Kit (ZYMO Research, Beijing, China) and reverse transcribed using a QuantiTect reverse transcription kit (Qiagen, Shanghai, China) according to the manufacturer’s protocol. RT–PCR was performed with an SYBR premix Ex Taq Kit (TaKaRa, Kusatsu, Japan) according to the operation manual and amplified in an ABI 7500 using primers listed in Supplementary Table S5. Data from three biological replicates were analyzed following the ΔΔCT method.

### 4.6. Transcriptomic Analysis

For transcriptomic analysis, three 2-week-old seedlings from two or three different transgenic lines were sampled and total RNA were extracted. Experiments were conducted following standard procedures of Shanghai Applied Protein Technology, Co., Ltd. (APT, Shanghai, China). Briefly, total RNAs were extracted with RNAprep Pure Plant Kit (TIANGEN, Beijing, China) and quantified by NanoDrop 2000C spectrophotometer (Thermo Fisher Scientific, Waltham, MA, USA). RNA integrity was checked by RNA Nano 6000 Assay Kit of the Agilent Bioanalyzer 2100 system (Agilent Technologies, Santa Clara, CA, USA). For cDNA synthesis, a total 1 μg RNA for each sample was treated with DNase I to eradicate the genomic DNA and then used as a template for reverse transcription (QuantiTect Reverse Transcription Kit, Qiagen, China).

We added fragment buffer to break into short segments using short segment RNA as template. Sequencing libraries were created using NEB Next Ultra RNA Library Prep Kit following manufacturer′s instructions. The index codes were added to each sample. Paired-end cDNA libraries with an insert size of 300 bp were constructed for transcriptome sequencing and sequenced on Illumina HiSeq 4000 platform (Illumina Inc., San Diego, CA, USA) at Shanghai Applied Protein Technology, Co., Ltd. (APT, Shanghai, China).

## Data Availability

The raw sequence data can be downloaded at https://www.ncbi.nlm.nih.gov/geo/query/acc.cgi?acc=GSE207213 (accessed on 18 August 2022).

## Supplementary Materials

The following supporting information can be downloaded at: https://www.mdpi.com/article/10.3390/plants11172206/s1, Figure S1: CRISPR/Cas9-induced mutation types of YTHDFA clade (A), YTHDFB clade (B), YTHDFC clade (C), and YTHDC clade (D) mutants in rice T1 transgenic plants (18bp target sequences were showed in blue with PAM in red). Figure S2: The multiple-sequence alignment of wild-type and mutation-type YTHDFA clade proteins used in this study. CRISPR-Cas9 system induced mutations lead to a serial of amino acid changes and early termination. Red box above the sequences shows the YTH domain. Figure S3: The multiple-sequence alignment of wild-type and mutation-type YTHDFB clade proteins used in this study. CRISPR-Cas9 system induced mutations lead to a serial of amino acid changes and early termination. Red box above the sequences shows the YTH domain. Figure S4: The multiple-sequence alignment of wild-type and mutation-type YTHDFC clade proteins (A–E) and OsYTHDC1A (F) used in this study. CRISPR-Cas9 system induced mutations lead to a serial of amino acid changes and early termination. Red box above the sequences shows the YTH domain. Figure S5: (A,B) Expression fold change of OsYTHDF2C (A) and OsYTHDC1A (B) mRNA in knockdown plants. The values obtained with wild type plants were referred to as 1 and shown as mean ± SD. Figure S6: Quantification of several agricultural traits of mutants/RNAi plants except DFA clade mutants, including plant height (A), tiller number (B), heading date (C), spikelets number per panicle (D), setting rate (E), and 1000 grain weight (F). Values are shown as mean ± SD. The significance of difference was determined by Student’s-test (*, *p* < 0.05; **, *p* < 0.01). Figure S7: The Venn diagrams show unique and shared DEGs in YTHDFB (A) and YTHDFC (B) mutants. Figure S8: GO enrichment analysis of YTH mutants/knockdown plants. A–L represent for the GO enrichment results for genes differentially expressed in osythdf1a (A), osythdf2a (B), osythdf3a (C), osythdf1b (D), osythdf2b (E), osythdf3b (F), osythdf1c (G), RNAi-OsYTHDF2C (H), osythdf3c (I), osythdf4c (J), osythdf5c (K), and RNAi-OsYTHDC1A (L) compared with wild type. “Multicellular organismal development”, “Response to stress”, “Response to abiotic stimulus”, and “Response to biotic stimulus” were marked in black. Figure S9: Expression patterns of OsYTH genes under different abiotic stresses (salt, drought, PEG, cold) and exogenous ABA application. Expression data are means of three independent biological replicate and normalized with the relative expression levels at the same treatment time under mock treatment. Figure S10: OsYTH genes contribute to salt stress response progress. (A) percentage survival rates (Percentage is the ratio of number of plants with green new leaves to number of total plants) of WT/mutant seedlings after 150 mM salt stress treatment in three assays. About 20 seedlings were used in each assay. (B) phenotypes of WT/mutant seedlings under salt stress for 8 days and recovery for 7 days (assay 3); Table S1: Gene numbers of plant YTH genes. Table S2: Gene numbers of animal and yeast YTH genes. Table S3: YTH proteins used in phylogenetic analysis. Table S4: the relative expression level of 77 organismal-development-related genes used for heatmap analysis. Table S5: Primers used in this study. Table S6: Log2 expression changes of abiotic response genes in OsYTH mutants. Table S7: Log2 expression changes of biotic-response genes in OsYTH mutants.
