# Supplementary material for: YTH Domain Proteins Play an Essential Role in Rice Growth and Stress Response
Source: Plants (Basel). 2022 Aug 25;11(17):2206. doi: 10.3390/plants11172206 (PMC9460353; doi:10.3390/plants11172206)
Supplement: Supplementary file 1 [file plants-11-02206-s001.zip › Supplementary Figure revised.pdf]

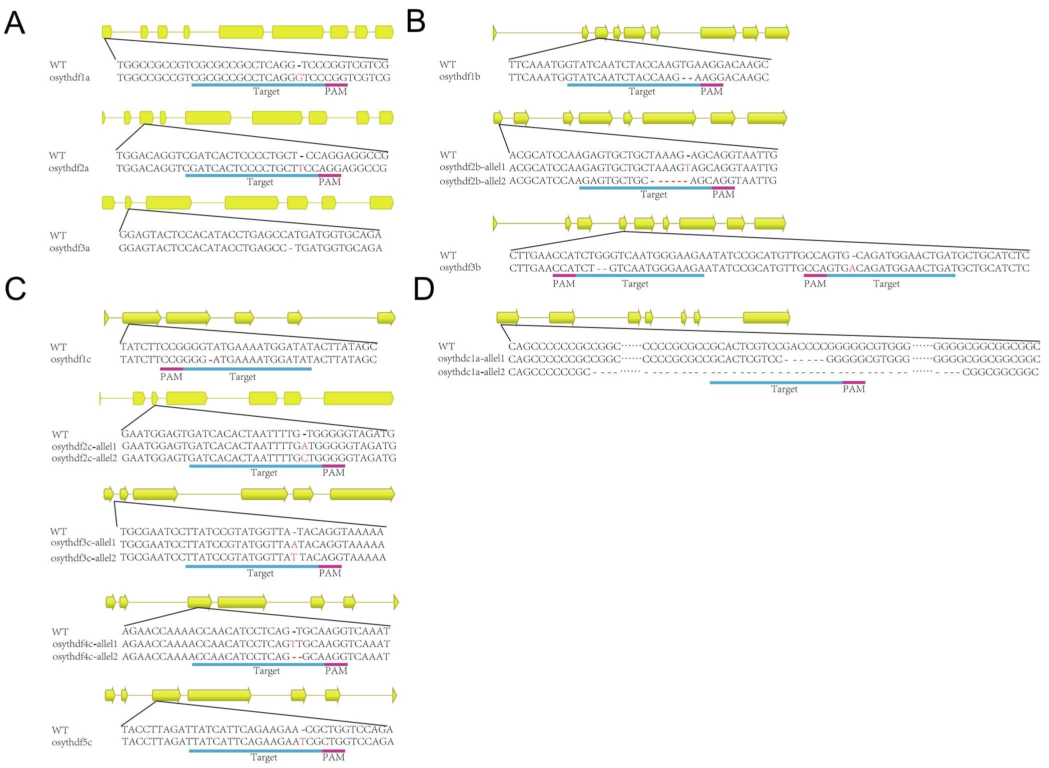

Supplementary Figure S1 CRISPR/Cas9-induced mutation types of YTHDFA clade (A), YTHDFB clade (B), YTHDFC clade (C), and YTHDC clade (D) mutants in rice T1 transgenic plants (18bp target sequences were showed in blue with PAM in red).

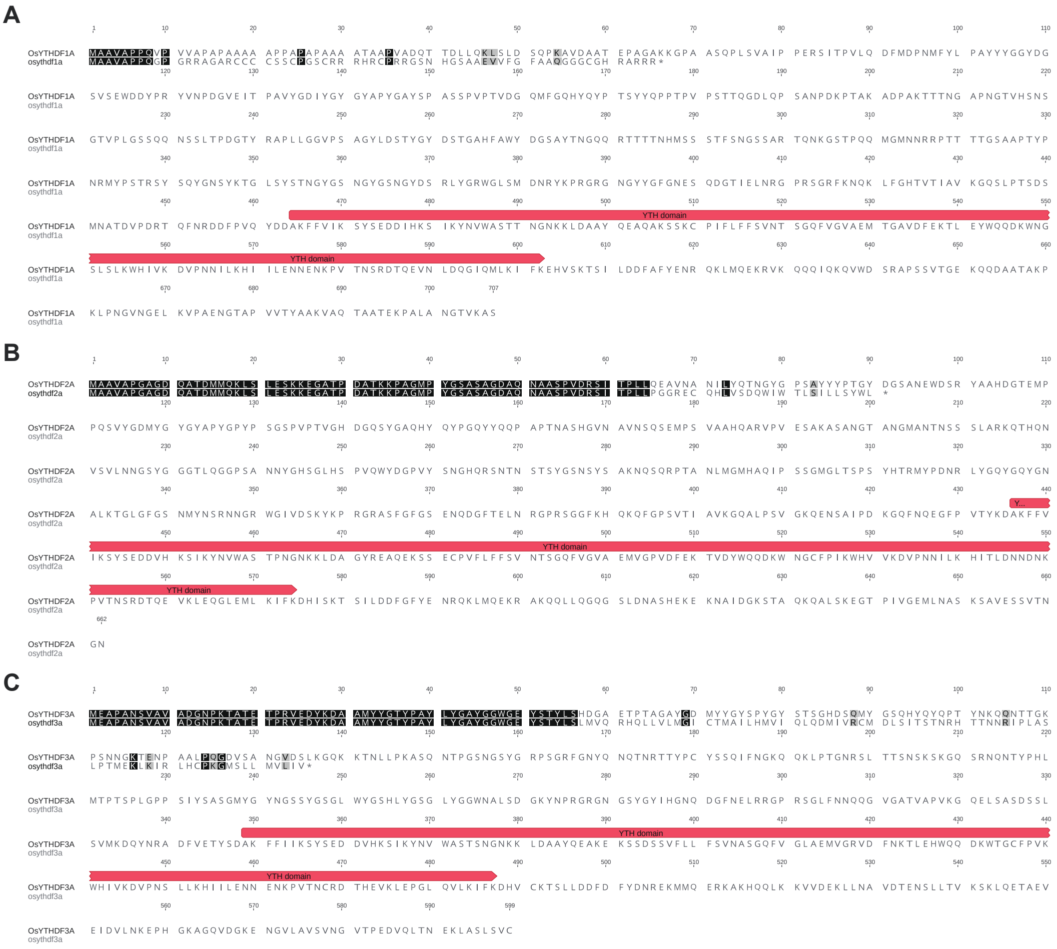

Supplementary Figure S2 The multiple-sequence alignment of wild-type and mutation-type YTHDFA clade proteins used in this study. CRISPR-Cas9 system induced mutations lead to a serial of amino acid changes and early termination. Red box above the sequences shows the YTH domain.

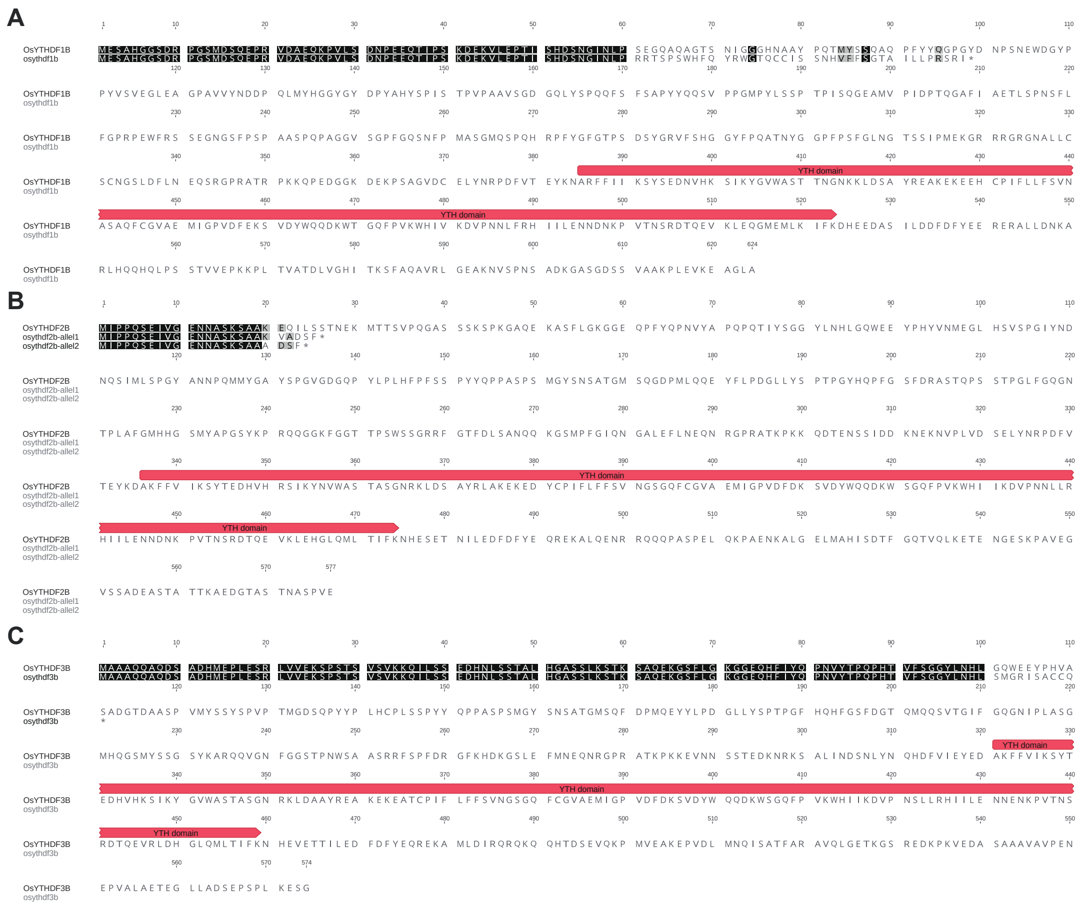

Supplementary Figure S3 The multiple-sequence alignment of wild-type and mutation-type YTHDFB clade proteins used in this study. CRISPR-Cas9 system induced mutations lead to a serial of amino acid changes and early termination. Red box above the sequences shows the YTH domain.

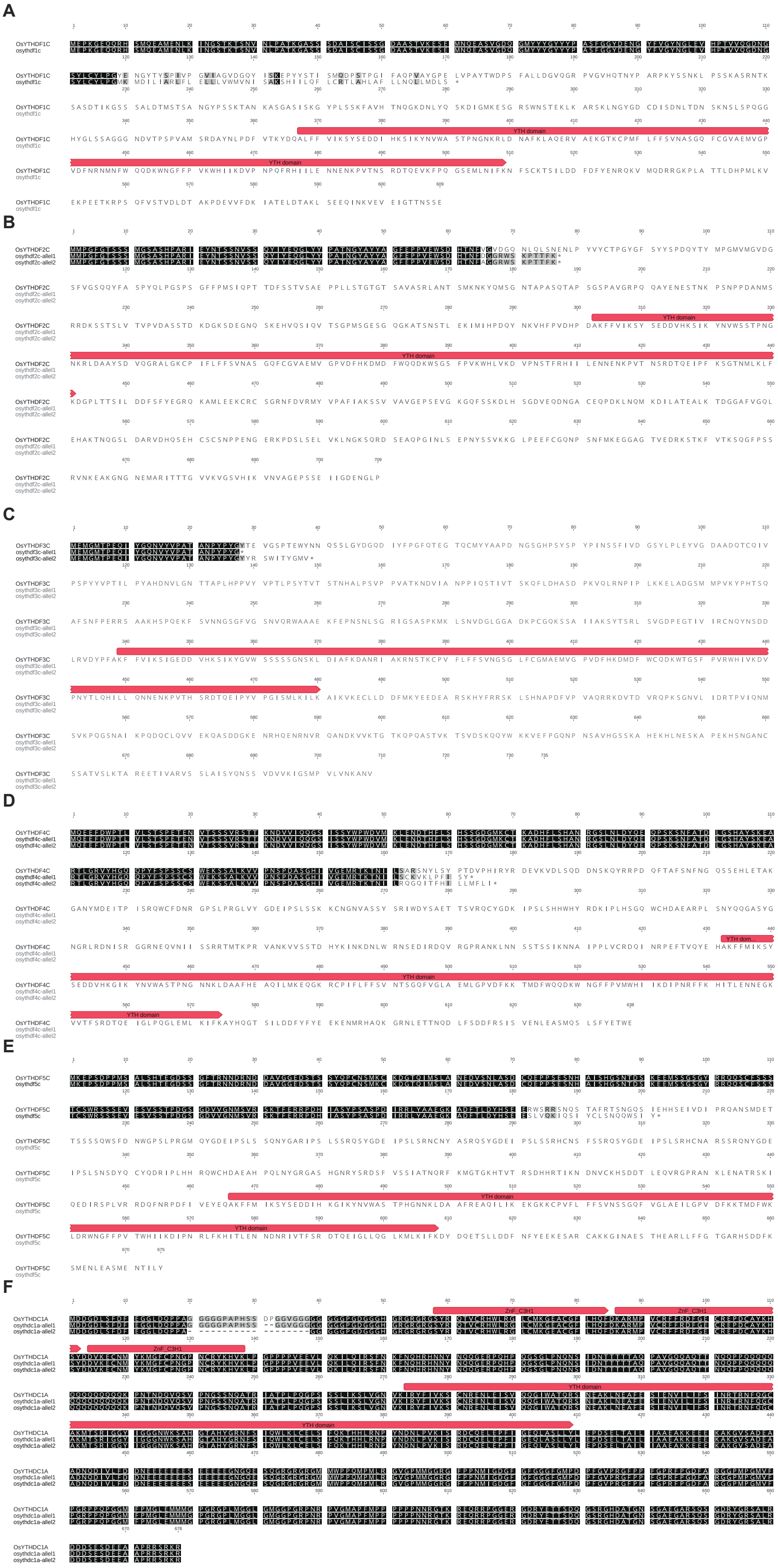
A
B
C
D
E
F
YTH domain
ZnF_C3H1

Supplementary Figure S4 The multiple-sequence alignment of wild-type and mutation-type YTHDFC clade proteins (A-E) and OsYTHDC1A (F) used in this study. CRISPR-Cas9 system induced mutations lead to a serial of amino acid changes and early termination. Red box above the sequences shows the YTH domain.

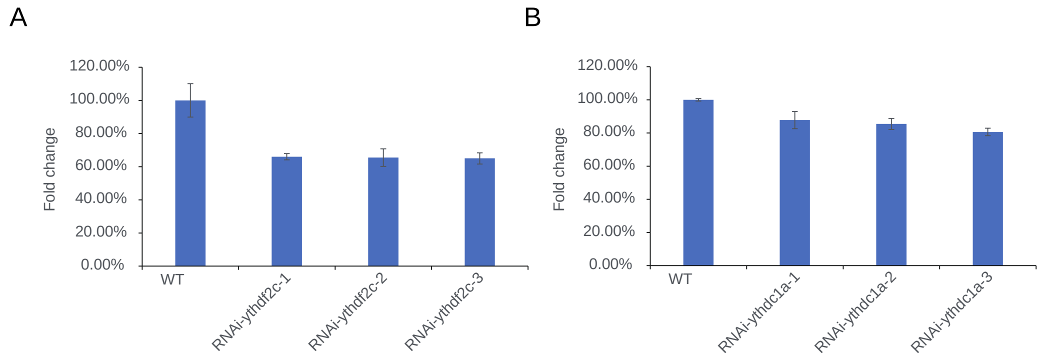

Supplementary Figure S5 (A-B) Expression fold change of OsYTHDF2C (A) and OsYTHDC1A (B) mRNA in knockdown plants. The values obtained with wild type plants were referred to as 1 and shown as mean ± SD.

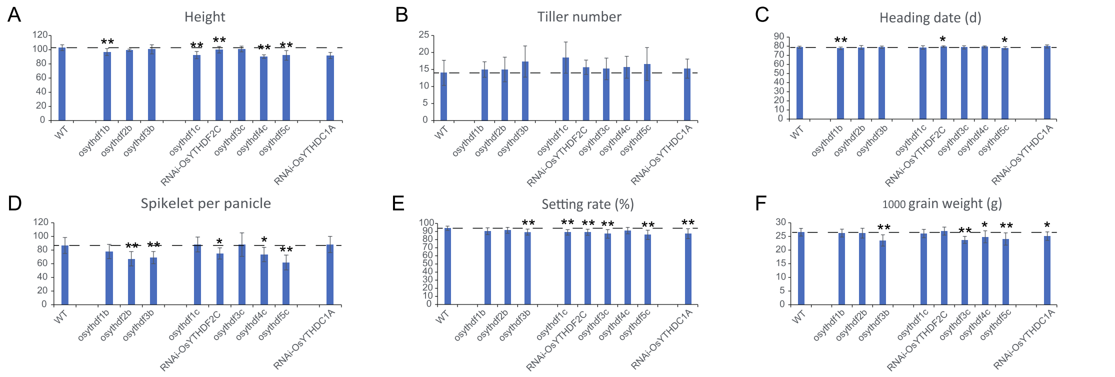

Supplementary Figure S6 Quantification of several agricultural traits of mutants/RNAi plants except DFA clade mutants, including plant height (A), tiller number (B), heading date (C), spikelets number per panicle (D), setting rate (E), and 1000 grain weight (F). Values are shown as mean ± SD. The significance of difference was determined by Student's-test (*, P< 0.05; **,P< 0.01).

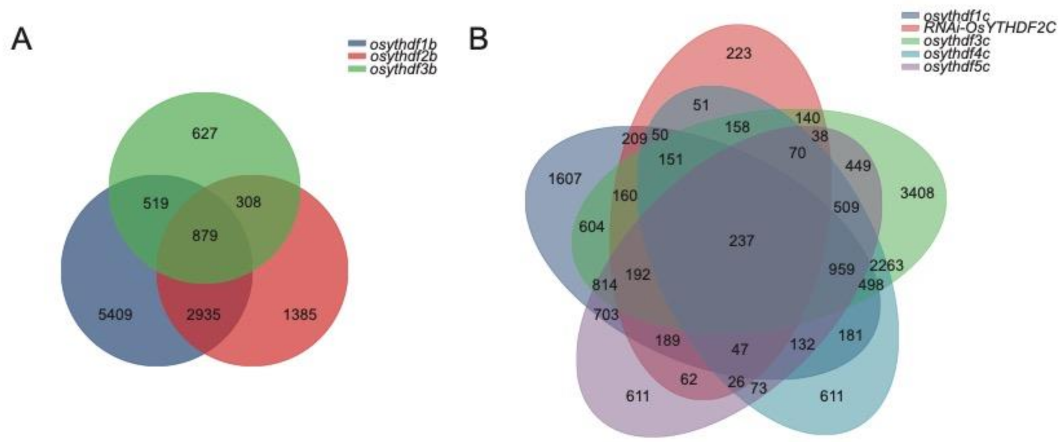

Supplementary Figure S7 The Venn diagrams show unique and shared DEGs in YTHDFB (A) and YTHDFC (B) mutants.

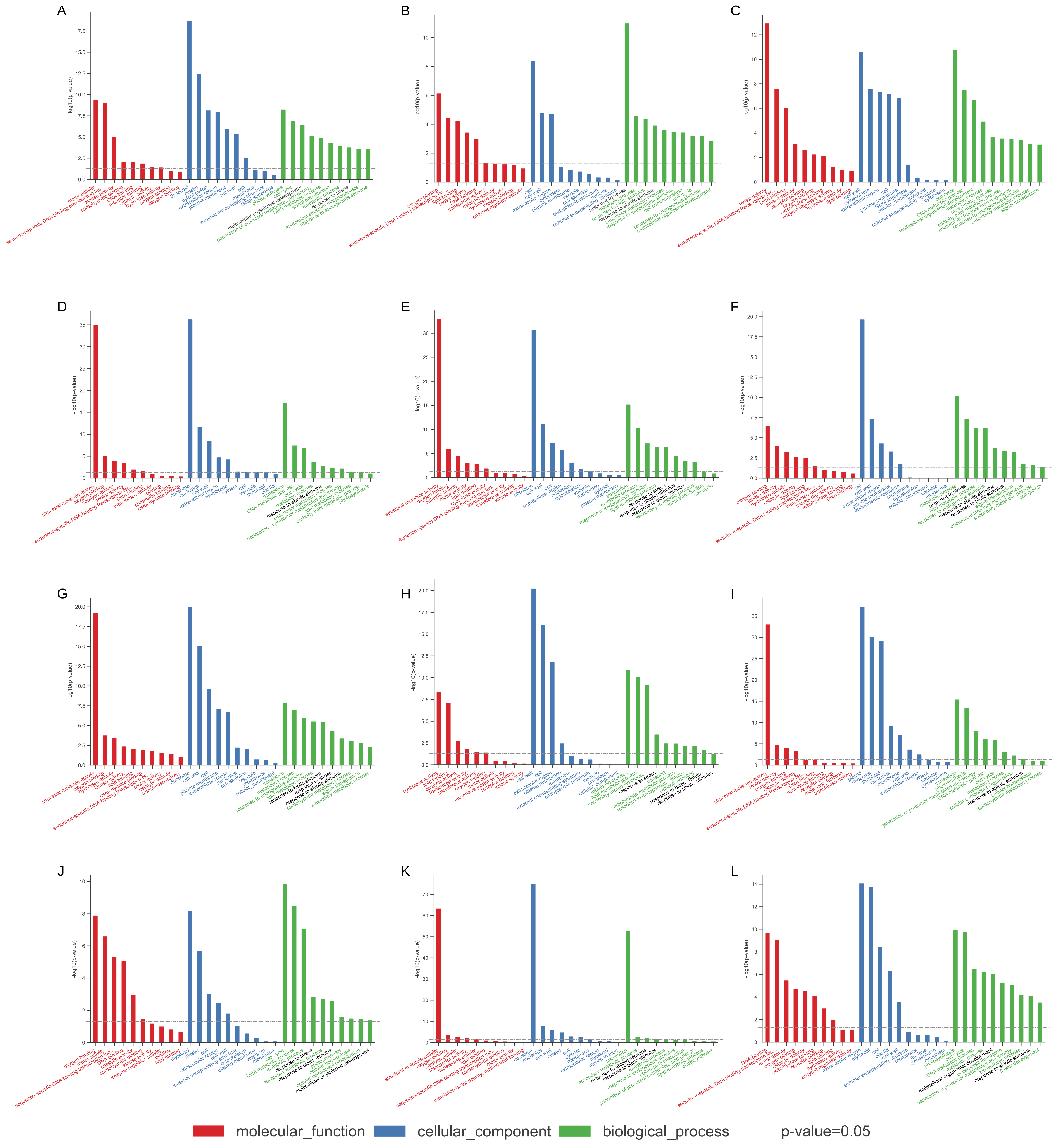

Supplementary Figure S8 GO enrichment analysis of YTH mutants/knockdown plants. A-L represent for the GO enrichment results for genes differentially expressed in osythdf1a (A), osythdf2a (B), osythdf3a (C), osythdf1b (D), osythdf2b (E), osythdf3b (F), osythdf1c (G), RNAi-OsYTHDF2C (H), osythdf3c (I), osythdf4c (J), osythdf5c (K), and RNAi-OsYTHDC1A (L) compared with wild type. “Multicellular organismal development”, “Response to stress”, “Response to abiotic stimulus”, and “Response to biotic stimulus” were marked in black.

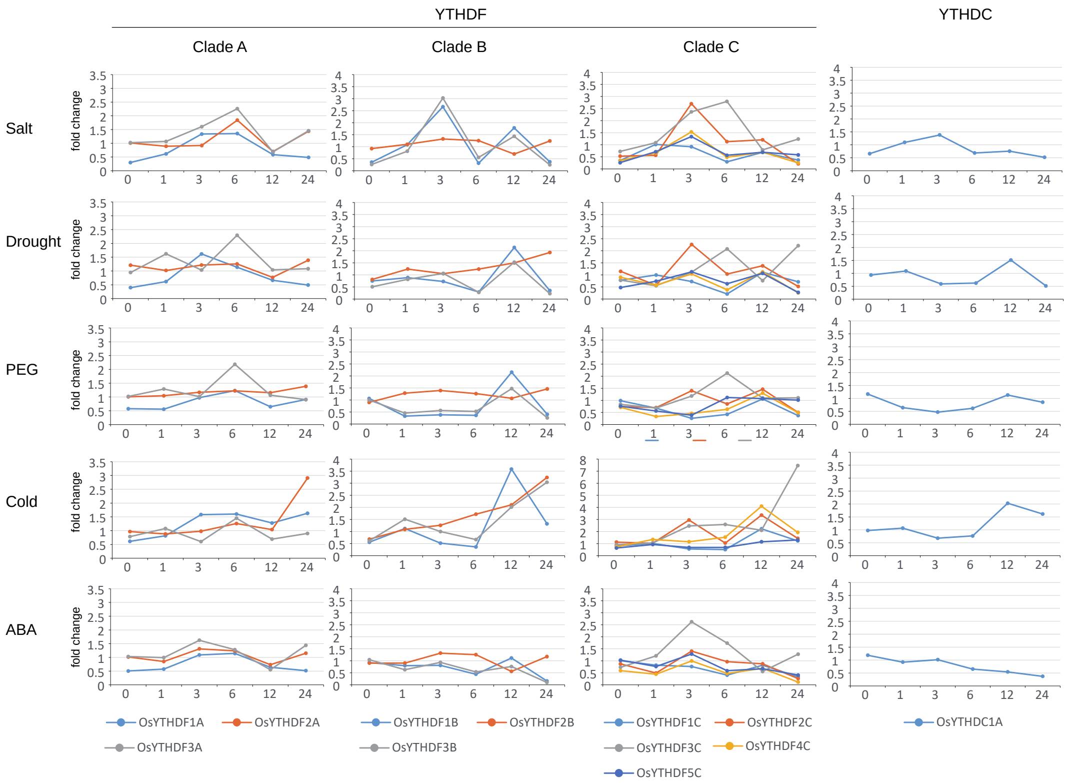

Supplementary Figure S9 Expression patterns of OsYTH genes under different abiotic stresses (salt, drought, PEG, cold) and exogenous ABA application. Expression data are means of three independent biological replicate and normalized with the relative expression levels at the same treatment time under mock treatment.

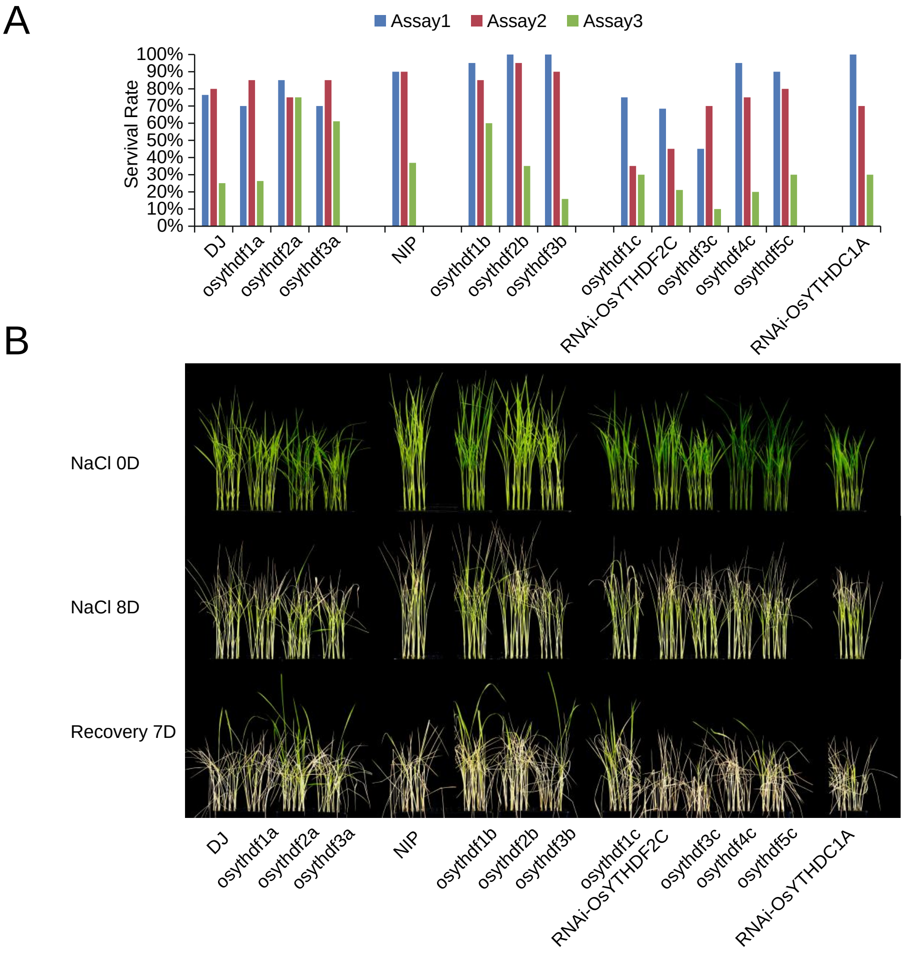

Supplementary Figure S10 OsYTH genes contribute to salt stress response progress. (A) percentage survival rates (Percentage is the ratio of number of plants with green new leaves to number of total plants) of WT/mutant seedlings after 150mM salt stress treatment in three assays. About 20 seedlings were used in each assay. (B) phenotypes of WT/mutant seedlings under salt stress for 8 days and recovery for 7 days (assay 3).
