# Supplementary material for: YTH Domain Proteins Play an Essential Role in Rice Growth and Stress Response
Source: Plants (Basel). 2022 Aug 25;11(17):2206. doi: 10.3390/plants11172206 (PMC9460353; doi:10.3390/plants11172206)
Supplement: Supplementary file 1 [file plants-11-02206-s001.zip › Supplementary Table revised.pdf]

Supplementary Table S1 Gene numbers of plant YTH genes

Taxon	Name	Brief		YTHDF						YTHDC		
			TOTAL	DF total	DFA	DFB	DFCI	DFCII	DFCIII	DC total	DC1	DC2
	Arabidopsis Thaliana	At	13	11	4	3	2	1	1	2	2	
	Capsella rubella	Cr	12	10	3	3	2	1	1	2	2	
	Boechera stricta	Bs	12	10	3	3	2	1	1	2	2	
	Brassica rapa	Br	22	18	7	5	1	4	1	4	4	
	Gossypium raimondii	Gr	13	11	5	3	2	0	1	2	2	
	Eucalyptus grandis	Eg	6	4	2	1	1	0	0	2	2	
	Populus trichocarpa	Pt	17	14	3	5	2	2	2	3	3	
	Salix purpurea	Sp	20	18	5	7	2	3	1	2	2	
	Linum usitatissimum	Lu	15	12	2	5	2	0	3	3	3	
Dicot	*Manihot esculenta*	Me	14	12	5	4	1	1	1	2	2	
	Glycine max	Gm	18	14	5	2	4	2	1	4	4	
	Phaseolus vulgaris	Pv	11	9	3	1	2	2	1	2	2	
	Medicago truncatula	Mt	9	8	3	1	2	1	1	1	1	
	Vitis vinifera	Vv	10	8	2	3	1	1	1	2	2	
	Solanum lycopersicum	Sl	7	4	1	1	1	0	1	3	3	
	Solanum tuberosum	St	8	4	1	1	1	0	1	4	4	
	Mimulus guttatus	Mg	10	7	2	1	2	0	2	3	3	
	Amaranthus hypochondriacus	Ah	9	7	2	2	1	0	2	2	2	
	Aquilegia coerulea	Ac	7	6	2	1	1	1	1	1	1	
	Zea mays	Zm	15	15	5	5	2	2	1	0	0	
	Setaria italica	Si	13	12	4	3	3	1	1	1	1	
	Panicum virgatum	Pvi	22	20	8	6	3	2	1	2	2	
	Panicum hallii	Ph	12	11	4	3	2	1	1	1	1	
Monocot	*Brachypodium stacei*	Bst	11	10	3	1	3	1	2	1	1	
	Oryza sativa	Os	12	11	3	3	2	1	2	1	1	
	Musa acuminata	Ma	19	17	7	5	2	2	1	2	2	
	Ananas comosus	Aco	9	8	1	2	2	1	1	1	1	
	Zostera marina	Zma	8	7	1	1	1	3	1	1	1	
Basal Angiosperm	*Amborella trichopoda*	Atr	7	5	1	1	1	1	1	2	2	
Fern	*Selaginella moellendorffii*	Sm	3	1	1					2	2	

Liverworts	*Marchantia polymorpha*	Mpo	3	1	1	2	2	
Moss	*Physcomitrella patens*	Pp	4	2	2	2	2	
	Sphagnum fallax	Sf	5	3	3	2	2	
Algae	*Chlamydomonas reinhardtii*	Cre	1	0	0	1	1	
	Dunaliella salina	Ds	1	0	0	1	1	
	Micromonas pusilla	Mp	1	1	1	0	0	
	Micromonas commoda	Mc	1	1	1	0	0	

Supplementary Table S2 Gene numbers of animal and yeast YTH genes

Taxon	Latin	Brief		YTHDF				YTHDC		
			TOTAL	DF total	YTHDF1	YTHDF2	YTHDF3	DC total	DC1	DC2
Mammalia	*Homo sapiens*	Hs	5	3	1	1	1	2	1	1
	Pongo abelii	Pa	5	3	1	1	1	2	1	1
	Macaca fascicularis	Mf	5	3	1	1	1	2	1	1
	Bos taurus	Bt	5	3	1	1	1	2	1	1
	Mus musculus	Mm	5	3	1	1	1	2	1	1
	Dipodomys ordii	Do	5	3	1	1	1	2	1	1
	Ornithorhynchus anatinus	Oa	5	3	1	1	1	2	1	1
Aves	*Gallus gallus*	Gg	5	3	1	1	1	2	1	1
Amphibia	*Xenopus tropicalis*	Xt	5	3	1	1	1	2	1	1
Osteichthyes	*Carassius auratus*	Ca	5	3	1	1	1	2	1	1
	Danio rerio	Dr	5	3	1	1	1	2	1	1
	Fundulus heteroclitus	Fh	6	4	2	1	1	2	1	1
	Nothobranchius furzeri	Nf	6	4	2	1	1	2	1	1
	Poecilia formosa	Pf	5	4	2	1	1	1	1	
	Oncorhynchus mykiss	Om	11	8	4	2	2	3	2	1
	Salmo salar	Ss	11	8	4	2	2	3	2	1
Chondrichthyes	*Callorhinchus milii*	Cm	5	3	1	1	1	2	1	1
Cephalochordata	*Branchiostoma floridae*	Bf	2	1	1			1	0	1
Ascidiacea	*Ciona intestinalis*	Ci	3	1	1			2	1	0
Echinodermata	*Strongylocentrotus purpuratus*	Spu	3	1	1			2	1	0
	Acanthaster planci	Ap	3	1	1			2	1	1
Brachiopoda	*Lingula anatina*	La	3	1	1			2	1	1
Mollusca	*Octopus bimaculoides*	Ob	3	1	1			2	1	1
	Mizuhopecten yessoensis	My	3	1	1			2	1	1
	Pomacea canaliculata	Pc	3	1	1			2	1	1
	Aplysia californica	Aca	3	1	1			2	1	1
	Crassostrea gigas	Cg	3	1	1			2	1	1
	Biomphalaria glabrata	Bg	3	1	1			2	1	1
Insecta	*Drosophila hydei*	Dh	2	1	1			1	1	0
	Drosophila melanogaster	Dm	2	1	1			1	1	0
	Stomoxys calcitrans	Sca	3	1	1			2	1	0

	Lucilia cuprina	Lc	3	1	1	2	1	0
	Habropoda laboriosa	Hl	3	1	1	2	1	0
	Pieris rapae	Pr	3	1	1	2	1	1
	Frankliniella occidentalis	Fo	3	1	1	2	1	1
	Hyposmocoma kahamanoa	Hk	3	1	1	2	1	1
	Bombyx mori	Bm	3	1	1	2	1	1
	Tribolium castaneum	Tc	3	1	1	2	1	1
Priapulida	*Priapulus caudatus*	Pca	3	2	1	1	1	0
Nematoda	*Caenorhabditis elegans*	Ce	1	0	0	1	0	0
Platyhelminthes	*Clonorchis sinensis*	Cs	2	1	1	1	1	0
	Opisthorchis viverrini	Ov	2	1	1	1	1	0
	Schistosoma haematobium	Sh	2	1	1	1	1	0
Cnidaria	*Orbicella faveolata*	Of	3	1	1	2	1	1
	Exaiptasia pallida	Ep	3	1	1	2	1	1
	Stylophora pistillata	Spi	2	1	1	1	0	1
Porifera	*Amphimedon queenslandica*	Aq	2	1	1	1	1	0
YEAST	*Saccharomyces cerevisiae*	Sc	1	0	1	0	0	0
	Schizosaccharomyces pombe	Sp	1	0	0	0	1	0

Supplementary Table S3 YTH proteins used in phylogenetic analysis

Species name	Accession number*	name code**	YTH motif
Ananas Comosus	Aco001739.1	AcoYTHDF1C	AK------------FFVIKSYSEDDVHK--SIKYSVWSSTVSGNRKLECAFE---DARKR-SAG--KTRNCP-----VFLFFS---VNASGQFC-----------------------------------GVAEMVGPVDFHTDM-DFWQ-QD-------------------------------------KW-SGCFPVKWHIIKDVSNAK-LRHIILENNENK-PVTNSRDTQEIP-----------------------YNTGINMLKIF
	Aco003339.1	AcoYTHDF4C	AK------------FFMIKSFSEDDVQK--SIKYNVWASTTYGNKKLDMAFR---DAQMV-VKE--KGTKCP-----IFLFFS---VNTSGQFV-----------------------------------GLAEMIGPVDFKKNM-EFWQ-QD-------------------------------------RW-NGFFPLTWHIIKDIPNRH-FHSIKLENNDNQ-VVTFSRDTQEIG-----------------------LPQGLKMLEIF
	Aco004279.1	AcoYTHDF1A	AK------------FFVIKSYSEDDVHK--SIKYNVWASTPNGNKKLDAGYQ---EAQEK-A---GGCP-----VFLFFS---VNTSGQFV-----------------------------------GVAEMVGPVDFNKTV-EYWQ-QD-------------------------------------KW-NGCFPVKWHIVKDVPNSI-LKHITLENNDNK-PVTNSRDTQEVK-----------------------LEQGLEMLKIF
	Aco004441.1	AcoYTHDF2C	AL------------FFVIKSYSEDDIHK--SIKYNVWSSTPNGNRRLDNAFQ---VGQEK-SRE---KGTKCP-----VFLFFS--VNASGQFC-----------------------------------GLAEMIGRVDFSKNM-DFWQ-QD-------------------------------------KW-NGYFPVKWHIIKDIPNPQ-FRHLILENNDNK-PVTNSRDTQEVK-----------------------FPQGTEMLSIF
	Aco007343.1	AcoYTHDF3C	AK------------FFIIKSYSEDDVHK--SIKYNVWSSTPNGNKKLDAAYE---DAQRL-SAL--KGVNCP-----VFLFFS--VNGSGRFC-----------------------------------GVAEMLGRVDFQRDM-DFWQ-QD-------------------------------------KW-SGSFSVKWHIIKDVPNTN-FRQITLENNENK-PVTHSRDTQEMY-----------------------IP-GTTMLKIF
	Aco007480.1	AcoYTHDF2B	AM------------FFVIKSFSEDNIHK--SIKYGVWASTPSGNKKLDSAYH---GAKER--GDPCP---VNASGHFC-----------------------------------GVAEMIGPVDFEKSV-DFWQ-QN-------------------------------------RW-SGCFPVKWRIVKDVPNNL-FRHIVLENNDNK-PVSNSRDTQEVN-----------------------LEQGLEMLNIF
	Aco008320.1	AcoYTHDF1B	AR------------FFIIKSYSEDNVHK--SIKYGVWASTANGNKKLDSAFH---EAKER--EDPYP-----IFLFFS---VNASGQFC-----------------------------------GVAEMVGPVDFEKSV-DYWQ-QD-------------------------------------KW-TGQFPVKWHIVKDVPNNL-FRHIILENNENK-PVTNSRDTQEVK-----------------------LEQGLEMLTIF
	Aco008423.1	AcoYTHDC1A	SR------------YFIVKSCNRENLEI--SVQQGIWATQRSNEAKLNEAFE---SSEN---VILIFS--INRTRHFQ-----------------------------------GCAKMTSKIGGFIGG-GNWK-SA--------------------------HGTAH--------Y-GRNFSVKWLKLCELSFNK-THHLRNPYNDNL-PVKISRDCQELE-----------------------PFIGEQLASLL
	Aco009712.1	AcoYTHDF2A	AK------------FFIIKSYSEDDVHK--SIKYNVWTSTPNGNKKLDAAYQ---EAQAR-T---GGCP-----IFLFFS---VNTSGQFV-----------------------------------GVAEMVGLVDFNKTV-DYWQ-QD-------------------------------------KW-HGCFSVKWHVVKDVPNSI-LKHITLENNENK-PVTNSRDTQEVR-----------------------LEQGLEMLKIF
Amaranthus hypochondriacus	AHYPO_001321-RA	AhYTHDF1B	AK------------FFIIKSFSEDNIHR--SIKYGVWASTPHGNRKLDAAYQ---EAKEK--GNCP-----VFLLFS---VNASGQFC-----------------------------------GVAEMVGPVDFDKNA-DYWQ-QD-------------------------------------RW-TGQFAVRWHIIKDIPNSK-FRHILLENNDHK-PVTHSRDSQEVN-----------------------QEQGIDMLRIF
	AHYPO_001693-RA	AhYTHDF3C	AK------------FYVIKSYSEVNVYN--CIKYNVWSSTEYGNKKLDAAFR---DAEIK-ANE---TGVKCP-----IFLFFS--VNGSGQFV-----------------------------------GVAEMIGKVDFNKTM-KFWQ-LD-------------------------------------KW-SGFFPLKWHIVKDVPNSQ-LRHILLENNENK-PVTFTRDTQEIG-----------------------LKHGLEMLKIF

	AHYPO_003351-RA	AhYTH DF2B	AK------------FFIIKSFSEDNIHR--SIKYGIWASTPHGNRKLDAAYQ---EAKEK---GDYP-----VFLLFS--VNASGQFC-------------------------------------GVAEMTGPVDFEKNA-DYWQ-QD--------------------------------------RW-TGQFTVRWHLIKDIPNSK-FRHILLENNDNK-PVTHSRDAQEVK------------------------LDLGIEMLKIF
	AHYPO_010570-RA	AhYTH DF1A	AK------------FFVIKSYSEDDVHK--SIKYNVWSSTPNGNKKLDLSYK---EAQQK-S---GGCP-----VFLFFS--VNTSGQFV--------------------------------------GLAEMVGPVDFHKNV-EHWQ-QD---------------------------------------KW-YGSFPVKWHIVKDVPNSL-LKHIILENNENK-PVTNSRDTQEVK------------------------LEQGLQILRIF
	AHYPO_011235-RA	AhYTH DF2C	AK------------FYVIKSYSEDDVHK--SIKYNVWSSTANGNKKLDAAFH--DAEVK-ANE---TGVKCP-----IFLFFS--VNGSGQFV--------------------------------------GVAEMTGKVDFKKNM-GFWQ-QD---------------------------------------KW-SGFFPVNWHIVKDVPNSQ-LRHILLENNDRK-PVTYTRDTQEIE------------------------LKEGLDMLKIF
	AHYPO_012466-RA	AhYTH DC1A	TR------------YFIVKSCNRENLEL--SVQQGVWATQRSNEAKLNEAFD--SVEH---VILIFS--VNRTRHFQ--------------------------------------GCAKMTSKIGETASG-GNWK-HA--------------------------HGTAH--------Y-GRNFSVKWLKLCELSFNK-TRHLRNPYNENL-PVKISRDCQELE-------------------------PSVGEQLASLL
	AHYPO_014122-RA	AhYTH DF1C	AK------------FFVIKSYSEDDVHK--SIKYNVWSSTPNGNKKLSIAYE--DAQKL-AAG---TPGGCP-----IFLFFS--VNASGQFC--------------------------------------GVAEMVGPVDFDKDM-DFWQ-QD---------------------------------------KW-SGSFSVKWHIIKDVPNPT-FRHIILENNEHK-PVTNSRDTQEIM------------------------LKQGLDMLKLF
	AHYPO_014607-RA	AhYTH DF2A	AK------------FFVIKSYSEDDVHK--SIKYNVWASTPTGNQKLDAAYQ--EAKGK-T--DACP-----VFLLFS--VNASGQFV--------------------------------------GLAEMVGPVDFNKNM-DFWQ-QD---------------------------------------KW-TGCFPVKWHIVKDVPNSL-LKHITLENNDNK-PVTNSRDTQEVK------------------------LEQAVQVVKIF
	AHYPO_015768	AhYTH DC1B	TR------------YFIIKSLNHENIQL--SIERGIWATQVMNEPILEEAFY--NCHK---VILIFS--VNSSGFFQ--------------------------------------GYAQMMSSVGRRRD--NVWT-QG--------------------------TGANN-------PW-GRSFRVKWLRSSNLAFHK-TLHLKNPLNEFK-PVKISRDCQELP------------------------PEIGEALCQLI
	Aqcoe1G060600	AcYTH DF1A	AK------------FFIIKSYSEDDVHK--SIKYSVWASTPSGNKKLNAGYQ--ESQEK-S--GDCP-----VFLCFS--VNGSGQFV--------------------------------------GLAEMVGPVDFDKSV-EYWQ-QD---------------------------------------KW-NGCFPLKWHIVKDIPNSL-LKQITLENNDNK-PVTNSRDTQEVK------------------------LEQGLQMLKIF
	Aqcoe1G157200	AcYTH DF2C	AL------------FFVIKSYSEDDIHK--SIKYNVWASTPNGNKRLDSAYQ--DAQEK-MGQ---KGSKCP-----VFLFFS--VNASGQFC--------------------------------------GVAEMIGRVDFNKNM-DFWQ-QD---------------------------------------KW-NGFFPVKWHIIKDVPNPH-FRHIILENNDNK-PVTNSRDTQEVK------------------------FPQGLEMLNVF
Aquilegia corulea	Aqcoe1G183700.1	AcYTH DC1A	SR------------YFVVKSCNRENLEL--SAQQGVWATQRSNEAKLNEAFD--SAEN---VILIFS--VNRTRHFQ--------------------------------------GCAKMTSKIGEISGG-GNWK-YA--------------------------HGTAH--------Y-GRNFSVKWLKLCELSFHK-TRHLRNPYNDNL-PVKISRDCQELE-------------------------PSIGQQLASLL
	Aqcoe2G257600	AcYTH DF1C	GK------------FFVIKSYSEDDVHK--SIKYNVWSSTPNGNKRLDSAYE--DAQRI-ASG---NSRGCP-----VFLFFS--VNASGQFC--------------------------------------GVAEMVGRVDFNKDM-EFWQ-QD---------------------------------------KW-SGSFPVKWHIIKDVPNTN-FRHIILENNENK-PVTNSRDTQEIK------------------------YRQGTEMLKIF
	Aqcoe6G010000	AcYTH DF3C	AK------------FFIIKSYSEDDIHR--SIKYDVWSSTPHGNKKLNAAFH--DAATI-YSE--TGTRCP-----IFLFFS--VNGSGQFL--------------------------------------GFAEMIGPVDFEKDM-DFWQ-QD---------------------------------------KW-NGFFPVTWHVIKDIPNNR-LRHIILENNDSK-PVTYTRDTQEVG------------------------LKQGLEMLSIF

	Aqcoe7G241300	AcYTH DF1B	GK------------FFVIKSYSEDNVHK--SIKYGVWASTPNGNKKLDAAYH---EAKEK--------------------------------------<br>-----------H--CP-----VFLLFS---VNASAQFC--------------------------------------GVAEMVGPVDFDKSV-<br>DYWQ-QD---------------------------------------KW-SGQFPVKWHIIKDVPNSQ-FRHIILENNDSK-PVTNSRDTQEVK------------------------LEQGLEMLNIF
	Aqcoe5G460600	AcYTH DF2A	AK------------FFIIKSYSEDDVHK--SIKYSVWASTPNGNKNLNAGYQ---ESQEK-S-------------------------------------<br>----------GDCP-----VFLCFS--VNGSGQFV---------------------------------------GLAEMVGPVDFDKSV-<br>EYWQ-QD---------------------------------------KW-NGCFPVKWHIVKDIPNSL-LKQITLENNDNK-PVTNSRDTQEVK-------------------------LEQGLQMLKIF
Arabidopsis thaliana	AT1G09810	ECT11 (AtYTH DF4C)	AK------------FFVIKSYSEDDVHK--SIKYSVWSSTINGNKKLDAAFR---DAETK-TLE-------------------------------<br>----------DGKKRP-----IFLFFS--VNASRQFV--------------------------------------GLAEMVGYVDFNKDL-<br>DFWQ-VD---------------------------------------KW-SGFFPVEWHVVKDIPNWE-LRHIILDNNEDK-PVTHTRDTHEIK------------------------LKEGLQMLSIF
	AT1G27960	ECT9 (AtYTH DF2B)	AK------------FFVIKSYSEDNVHK--SIKHCVWASTKNGNKKLDAAYR---EAKKK-------------------------------------<br>-----------DVACP-----VFLLFS--VNASSQFC--------------------------------------GVAEMVGPVDFNTSV-<br>EYWQ-QD---------------------------------------RW-SGHFPVQWLIVKDVPNSL-FRHIIIESNDNK-PVTNSRDTQEVG------------------------LEKGIEMLDIF
	AT1G30460	CPSF30 (AtYTH DC1A)	NR------------YFVVKSNNRENFEL--SVQQGVWATQRSNEAKLNEAFD--SVEN---------------------------------------<br>--------------------VILIFS--VNRTRHFQ--------------------------------------GCAKMTSRIGGYIGG-GNWK-<br>HE--------------------------HGTAQ--------Y-GRNFSVKWLKLCELSFHK-TRNLRNPYNENL-PVKISRDCQELE-------------------------PSVGEQLASLL
	AT1g48110	ECT7 (AtYTH DF2C)	AK------------FFVIKSYSEDDVHK--SIKYNVWSSTLHGNKKLQSAYE--DAQRI-ATE------------------------------<br>-----------KSCECP-----IFLFFS--VNASGLFC--------------------------------------GMAEMTGPVSFDKDM-<br>DFWQ-QD---------------------------------------KW-SGSFPVKWHIIKDVPNSY-FRHIILQNNENK-PVTNSRDTQEIM------------------------LKQGLEVLKIF
	AT1G55500	ECT4 (AtYTH DF4A)	AK------------FFIIKSYSEDDVHK--SIKYNVWASTPNGNKKLDAAYQ---EAQQK-S-----------------------------------<br>------------SGCP-----VFLFFS--VNASGQFI--------------------------------------GLAEMKGPVDFNKNI-<br>EYWQ-QD---------------------------------------KW-TGSFPLKWHILKDVPNSL-LKHITLEYNENK-PVTNSRDTQEVK-------------------------LEQGLKVVKIF
	AT1G79270	ECT8 (AtYTH DF3C)	AI------------FFVIKSYSEDDIHK--SIKYNVWSSTLNGNKKLDSAYQ--ESQKK-AAD-------------------------------<br>----------KSGKCP-----VFLFFS--VNASGQFC--------------------------------------GVAEMIGRVDYEKSM-<br>EFWQ-QD---------------------------------------KW-TGYFPVKWHIIKDVPNPQ-LRHIILENNENK-PVTNSRDTQEVR------------------------LPQGNEVLNIF
	AT3G03950	ETC1 (AtYTH DF1A)	AK------------FFVIKSYSEDDVHN--CIKYGAWSSTPTGNKKLNAAYY---EAKEN-S-----------------------------------<br>------------QECP-----VYLLFS---VNASGQFV--------------------------------------GLAEMVGPVDFNKTM-<br>EYWQ-QD---------------------------------------KW-IGCFPVKWHIIKDIPNSL-LRHITLANNENK-PVTNSRDTQEVN------------------------LEHGTKIIKIF
	AT3G13060	ECT5 (AtYTH DF1B)	AK------------LFIIKSYSEDNVHK--SIKYNVWASTPNGNKKLDAAYR---EAKDE--------------------------------------<br>----------KEPCP-----LFLLFS---VNASSQFC--------------------------------------GVAEMVGPVDFEKSV-<br>DYWQ-QD---------------------------------------KW-SGQFPVKWHIIKDVPNSQ-FRHIILENNDNK-PVTNSRDTQEV-----------------------------------
	At3g13460	ECT2 (AtYTH DF2A)	AM------------FFIIKSYSEDDVHK--SIKYNVWASTPNGNKKLAAAYQ---EAQQK-A---------------------------------<br>-------------GGCP-----IFLFFS--VNASGQFV--------------------------------------GLAEMTGPVDFNTNV-<br>EYWQ-QD---------------------------------------KW-TGSFPLKWHIVKDVPNSL-LKHITLENNENK-PVTNSRDTQEVK------------------------LEQGLKIVKIF
	AT3G17330	ECT6 (AtYTH DF1C))	AR------------FFVIKSYSEDDVHK--SIKYGVWSSTLNGNKKLQSVYE--DAQRI-ATE------------------------------<br>-----------KSRECP-----IFLFFS--VNSSGLFC--------------------------------------GVAEMTGPVSFDRDM-<br>DFWQ-QD---------------------------------------KW-SGSFPVKWHIIKDVPNSY-FRHIILHNNENK-PVTNSRDTQEII------------------------LKQGLEVLKLF

	AT4G11970	ECT12 (AtYTH DC1B)	TR------------YFIIKSLNYDNIQV--SVEKGIWATQVMNEPILEGAFH--KSGR--------------------------------------<br>----------------VILIFS---VNMSGFFQ--------------------------------------GYAEMLSPVGWRRD--QIWS-QG---<br>------------------------GGKNN-------PW-GRSFKVKWLRLSELPFQK-TLHLKNPLNDYK-PVKISRDCQELP-------------------------EDIGEALCELL
	AT5G58190	ECT10 (AtYTH DF3B)	AK------------FFIVKSFSEDNVHR--SIKYNVWASTPHGNKKLDTAYR--DAEKM------------------------------------<br>----------GGKCP-----IFLFFS---VNASGQFC--------------------------------------GVSEMVGPVDFEKDA-<br>GYWQ-QD---------------------------------------RW-SGQFPVKWHIVKDIPNNR-FCHILLQNNDNK-PVTHSRDSQEVK-------------------------LRQGIEMLRIF
	AT5G61020	ECT3 (AtYTH DF3A)	AK------------FYVIKSYSEDDIHK--SIKYSVWSSTPNGNKKLDASYN--EAKQK-S----------------------------------<br>-----------DGCP-----VFLLFS---VNTSGQFV--------------------------------------GLAEMVGPVDFNKTV-<br>EYWQ-QD---------------------------------------KW-IGCFPVKWHFVKDIPNSS-LRHITLENNENK-PVTNSRDTQEVK-------------------------LEQGIKVIKIF
Capsella rubella	Carubv10003808m	CrYTH DC1B	AK------------FFVIKSYSEDDIHK--SIKYNVWSSTPNGNKKLDASYN--EAKQK-S----------------------------------<br>-----------EGCP-----VFLLFS---VNTSGQFV--------------------------------------GLAEMVGPVDFNKTV-<br>EYWQ-QD---------------------------------------KW-IGCFPVKWHFVKDIPNSS-LRHITLENNENK-PVTNSRDTQEVK-------------------------LEQGMKVIKIF
	Carubv10008560m	CrYTH DF1C	AK------------FFIVKSFSEDNVHR--SIKYNVWASTPHGNKKLDTAYR--DAEKM------------------------------------<br>----------GGKCP-----IFLFFS---VNASGQFC--------------------------------------GVSEMAGPVDFEKDA-<br>GYWQ-QD---------------------------------------RW-SGQFPVKWHIVKDVPNNR-FCHILLQNNDNK-PVTHSRDSQEVK-------------------------LRQGIEMLRIF
	Carubv10008570m	CrYTH DC1A	AM------------FFVIKSYSEDDIHK--SIKYNVWSSTLNGNKKLDSAYQ--ESQKK-IAE-------------------------------<br>----------KNVKCP-----VFLFFS---VNASGQFC--------------------------------------GVAEMVGRVDYEKSM-<br>EFWQ-QD---------------------------------------KW-TGYFPVKWHMIKDVPNPQ-LRHIILENNENK-PVTNSRDTQEVR-------------------------LPQGNEVLNIF
	Carubv10008725m	CrYTH DF3B	AK------------FFVIKSYSEDDVHN--SIKYGAWSSTPTGNKKLNAAYY--EAKEN-S---------------------------------<br>------------QECP-----VYLLFS---VNASGQFV--------------------------------------GLAEMVGPVDFNKTM-<br>EYWQ-QD---------------------------------------KW-IGCFPVKWHIIKDIPNSL-LRHITLENNENK-PVTNSRDTQEVN-------------------------LDHGTKIIKIF
	Carubv10011018m	CrYTH DF4C	AK------------FFVIKSYSEDDVHK--SIKYGVWSSTLHGNKKLQSVYE--DAQRI-VTE------------------------------<br>-----------KSCECP-----IFLFFS---VNASGLFC--------------------------------------GVAEMTGPVSFNRDM-<br>DFWQ-QD---------------------------------------KW-SGSFPVKWHIIKDVPNSY-FRHIILQNNENK-PVTNSRDTQEIM-------------------------LKQGLEVLKLF
	Carubv10013132m	CrYTH DF1B	AM------------FFIIKSYSEDDVHK--SIKYNVWASTPNGNKKLAAAYQ--EAQQK-P--------------------------------<br>------------GGCP-----IFLFFS---VNASGQFV--------------------------------------GLAEMTGPVDFNTNV-<br>EYWQ-QD---------------------------------------KW-TGSFPLKWHIVKDVPNSL-LKHITLENNENK-PVTNSRDTQEVK-------------------------LEQGLKIVKIF
	Carubv10013133m	CrYTH DF1A	AK------------LFIIKSYSEDNVHK--SIKYNVWASTPNGNKKLDAAYR--EAKDE------------------------------------<br>----------KEPCP-----LFLLFS---VNASSQFC--------------------------------------GVAEMVGPVDFEKSV-<br>DYWQ-QD---------------------------------------KW-SGQFPVKWHIIKDVPNSQ-FRHIILENNDNK-PVTNSRDTQEVK-------------------------LEQGIEMLKIF
	Carubv10013212m	CrYTH DF3C	AK------------FFVIKSYSEDDVHK--SIKYSVWSSTINGNKKLDAAFR--DAETK-TRE-------------------------------<br>----------DGKKRP-----IFLFFS---VNASRQFV--------------------------------------GLAEMAGYVDFKKDL-<br>NFWQ-VD---------------------------------------KW-SGFFPVEWHVVKDIPNWE-LRHIILANNEDK-PVTHTRDTHEVK-------------------------LKEGLQMLSIF
	Carubv10013762m	CrYTH DF2A	AK------------FFVIKSYSEDNVYK--SIKYSVWASTKNGNKKLDAAYR--EAKTK-----------------------------------<br>-----------DVACP-----VFLLFS---VNASAQFC--------------------------------------GVAEMVGAVDFNTSV-<br>EYWQ-QD---------------------------------------RW-SGHFPVRWLIVKDVPNSL-FRHIIIENNDNK-PVTNSRDTQEVG-------------------------LEQGIEMLNIF

	Carubv10021946m	CrYTH DF2C	NR------------YFVVKSCNRENFEL--SVQQGVWATQRSNEAKLNEAFD---SVEN---------------------------------------<br>--------------------VILIFS---VNRTRHFQ-------------------------------------GCAKMTSRIGGYIGG-GNWK-<br>HE--------------------------HGTAQ--------Y-GRNFSVKWLKLCELSFHK-TRNLRNPYNENL-PVKISRDCQELE------------------------PSIGEQLASLL
	Carubv10026226m	CrYTH DF2B	AK------------FFVIKSYSEDDVHK--SIKYNVWSSTLHGNKKLQSAYE---DAQKI-ASE-----------------------------<br>-----------KSCECP-----IFLFFS---VNASGLFC--------------------------------------GMAEMTGPVSFDKDM-<br>DFWQ-QD--------------------------------------KW-SGSFPVKWHIIKDVPNSY-FRHIILQNNENK-PVTNSRDTQEIM------------------------LKQGLEVLKIF
	Carubv10026294m	CrYTH DF3A	TR------------YFIIKSLNYENIQV--SVEKGIWATQVMNEPILEGAFH---KSGR---<br>----------------VILIFS--VNMSGFFQ-------------------------------------GYAEMLSPVGWRRD--HIWS-QG---<br>-----------------------GGKNN-------PW-GRSFKVKWLRLSELPFQK-TLHLKNPLNDYK-PVKISRDCQELP------------------------EDIGEALCELL
Drosophila melanogaster	sp|Q9VZQ1	DmYT HDC1	TR------------FFLIKSNNSDNVQL--SKNKSVWATLPQNDANLNQAFK--EARN---<br>-------------------VLLIFS---VNESGKFA-------------------------------------GFARMAAPSRRDIPQ-VAWV-<br>LP------------------------PSISPK-------AL-GGVIELDWICRKELSFNA-TLHLHNTWNEGK-PVKIGRDGQEIE------------------------PKIGGELCRLF
	CG6422-RA	DmYT HDF1	AR------------FFVIKSYSEDDIHR--SIKYEIWCSTDHGNKRLDDAFK--ERHEE---<br>---------GGN-----IMLFFS---VNGSGHFC--------------------------------------GMAQMMTPVDYNSTS-SVWS-<br>QD--------------------------------------KW-RGKFKVKWIYVKDVPNGT-LRHIRLENNENK-SVTNSRDTQEVP------------------------NDKGIEVLQIL
Chlamydomonas reinhardtii	Cre12.g516400.t1.2	CreYTH DC1	IR------------YFIIRSNSLQNIFI----SVRTGAWATTRTNDPKLDAAFR---SSRE---<br>------------------VRLIFS---VMGSNAFQ-------------------------------------GYATMRTSVGAFPKP--------------<br>----------------------VIWENG-------QQFGRPFGVEWRVLFELPHDD-CNHIRNRLNDNK-VVYMARDCTELP------------------------QEQGDLLTTIM
Dunaliella salina	Dusal.0106s00010.1	DsYTH DC1	VR------------YFIIRSYNQENVDI----SINVGGWATTKHNEIKLNEAYA---TCDE---------------------------------------<br>----------------------VRLVFS---VNSSNCFQ-------------------------------------GYAVMSTPVGQYRSI-----------<br>--------------------------IWSNG-------KAFGNAFGVDWRCVFNLEVWN-VKHLLNPLNENK-PVHVARDGQEVP------------------------TRVGDELVSIM
Homo sapiens	sp|Q9H6S0	HsYTH DC2	VR------------YFIMKSSNLRN--LEI--SQQKGIWSTTPSNERKLNRA---FW---ESSI--<br>--------------------VYLVFS--VQGSGHFQ-------------------------------------GFSRM---SSEIGREKSQ----------<br>------------------------DWGSA-------GL-GGVFKVEWIRKESLPFQF-AHHLLNPWNDNK-KVQISRDGQELE------------------------PLVGEQLLQLW
	sp|Q96MU7	HsYTH DC1	AR------------FFLIKSNNHEN--VSL--AKAKGVWSTLPVNEKKLNLA---FR---SARS---------------------------------------<br>-----------------------VILIFS---VRESGKFQ-------------------------------------GFARL---SSESHHGGSP-IHWV-<br>LP-------------------------AGMSAK-------ML-GGVFKIDWICRRELPFTK-SAHLTNPWNEHK-PVKIGRDGQEIE------------------------LECGTQLCLLF
	sp|Q9BYJ9	HsYTH DF1	GR------------VFIIKSYSEDD--IHR--SIKYSIWCSTEHGNKRLDSA---FR---CMSSK------------------------------------<br>--------------GP-----VYLLFS---VNGSGHFC-------------------------------------GVAEM---KSPVDYGTSA-<br>GVWS-QD-------------------------------------KW-KGKFDVQWIFVKDVPNNQ-LRHIRLENNDNK-PVTNSRDTQEVP------------------------LEKAKQVLKII
	sp|Q7Z739	HsYTH DF3	GR------------VFIIKSYSEDD--IHR--SIKYSIWCSTEHGNKRLDAA---YR---SLNGK------------------------------------<br>--------------GP-----LYLLFS---VNGSGHFC-------------------------------------GVAEM---KSVVDYNAYA-<br>GVWS-QD-------------------------------------KW-KGKFEVKWIFVKDVPNNQ-LRHIRLENNDNK-PVTNSRDTQEVP------------------------LEKAKQVLKII
	NP_057342	HsYTH DF2	GR------------VFIIKSYSEDD--IHR--SIKYNIWCSTEHGNKRLDAA---YR---SMNGK-----------------------------------<br>----------------GP-----VYLLFS---VNGSGHFC-------------------------------------GVAEM---KSAVDYNTCA-<br>GVWS-QD-------------------------------------KW-KGRFDVRWIFVKDVPNSQ-LRHIRLENNENK-PVTNSRDTQEVP------------------------LEKAKQVLKII

Eucalyptus grandis	Eucgr.B03590.1	EgYTH DF1A	AK------------FFIIKSYSEDD--VHK--SIKYSVWASTPNGNKKLDAA---YR--EAQEK-P---GSSP-----VFLFFS--VNTSGQFV-------------------------------------GLAEM---VGPVDFHKSV-EYWQ-QD-------------------------------------KW-NGCFPVKWHIVKDVPNSM-LKHITLENNENK-PVTNSRDTQEVK------------------------LVQGLKMIKIF
	Eucgr.B03591.1	EgYTH DF2A	AK------------FFIIKSYSEDD--VHK--SIKYNVWASTPNGNKKLDAA---YR--EAQEK-A--AGCP-----IFLFFS--VNTSGQFV-------------------------------------GLAEM---VGPVDFHKNV-EYWQ-QD-------------------------------------KW-MGCFPVKWHIVKDVPNNL-LKHITLENNENK-PVTNSRDTQEVK------------------------LPQGLKMIKIF
	Eucgr.D01949.1	EgYTH DC1B	IR------------YFIIKSLNHHN--LQL--SIEKGIWATQVMNEPILEEA---FH---NSGK---VILIFS--VNMSGFFQ-------------------------------------GYAQM---MSPIGWRRD--KVWS-QG-------------------------NGRNN-------PW-GRSFKVKWLRLYDLPFQK-TLHLKNLLNDNK-PVKISRDCQELP------------------------QDVGEALCELL
	Eucgr.E02496.1	EgYTH DC1A	SR------------YFIVKSCNREN--LEL--SVQQGVWATQRSNEAKLNEA---FD---SAAN---VILIFS--VNRTRHFQ-------------------------------------GCAKM---TSRIGGAASG-GNWK-YA-------------------------HGTAH--------Y-GRNFSVKWLKLCELSFQK-TRHLRNPYNENL-PVKISRDCQELE------------------------PSVGEQLASLL
	Eucgr.H04096.1	EgYTH DF1C	AK------------FFVIKSYSEDD--VHK--SIKYNVWSSTPHGNKKLQSA---YE--DAQKI-AAE---KTTNCP-----IFLFFS--VNASGQFC-------------------------------------GVAEM---VGQVDFHKNM-DFWQ-QD-------------------------------------KW-TGSFPVKWHIIKDVQNTA-FRHIILENNENK-PVTNSRDTQEIM------------------------QKQGLEMLKIF
	Eucgr.J03195.1	EgYTH DF1B	AK------------FFVIKSYSEDN--VHK--SIKYGVWASTPNGNRKLDAA---YR--EAKEK--PDTCP-----VFLFFS--VNASAHFC-------------------------------------GVAEM---VGPVDFDRSF-DYWQ-QD-------------------------------------RW-SGQFPVKWHVIKDVPNSQ-LRHIVLENNDNK-PVTNSRDTQEVK------------------------LEQGIEMLNIF
Amborella trichopoda	evm_27.model.AmTr_v1.0_scaffold00001.426	AtrYTH DC1B	AF------------FFVIKSYSEDD--VHK--SIKYNVWASTPNGNKRLDNA---YQ--DAQQK-ALE---KGSKCP-----VFLFFS--VNASGQFC-------------------------------------GVAEM---MGRVDFNQNM-DFWQ-QD-------------------------------------KW-NGFFPVKWHVIKDVPNPQ-FRHIILENNDNK-PVTNSRDTQEVK------------------------FPQGIEMLNIF
	evm_27.model.AmTr_v1.0_scaffold00004.54	AtrYTH DF1A	AK------------FFIIKSYSEDN--VHK--SIKYGVWASTANGNRKLDAA---YR--ESKEK---DGHCP-----VFLLFS--VNASAQFC-------------------------------------GVAEM---MGPVDFEKSV-DYWQ-QD-------------------------------------KW-SGQFPVKWHIVKDVPNSQ-FRHIILENNDNK-PVTNSRDTQEVK------------------------LEQGLEILKIF
	evm_27.model.AmTr_v1.0_scaffold00025.287	AtrYTH DF1B	AK------------FFIIKSYSEDD--IHK--SIKYSVWASTPNGNKKLDAA---YK--EAQEK-T--GGCP-----VFLFFS--VNASGQFL-------------------------------------GVAEM---VGPVDFNKNV-EFWQ-QD-------------------------------------KW-NGCFPVKWHVVKDVPNNQ-LKHIILENNDNK-PVTNSRDTQEVK------------------------FDQGIEILKIF
	evm_27.model.AmTr_v1.0_scaffold00068.160	AtrYTH DF2C	TR------------YFVIKSLNHQN--IQL--SIEKGIWATQVMNEQILEEA---FH---SSDR---VILIFS--VNMSGFFQ-------------------------------------GYAQM---MSPVGWRRE--NVWS-GS-------------------------SGGSN-------PW-GRTFKVKWLQLNNLAFQK-TLHLKNPLNDYK-PVKISRDCQELT------------------------QEVGEALCELL
	evm_27.model.AmTr_v1.0_scaffold00071.175	AtrYTH DF3C	AL------------FYVIKSFSEDD--VHK--SLKYNVWASTDFGNKKLDEA---FH---GAQAK-LND--NGSRCP-----VFLFFS--VNGSGQFV-------------------------------------GLAEM---

			TCKVDFKKTL-DFWQ-GD------------------------------------VW-NGFFPVKWHIVKDVPNHL-LTHIILKDNENK-AVTYSRDTQEVT-----------------------FSQGIEMLKIF
	evm_27.model.AmTr_v1.0_scaffold00096.42	AtrYTHDF1C	SR-----------YFIVKSCNREN--LEL--SVQKGIWATQRSNESKLNEA---FD--SSEN---VVLIFS--INRTRHFQ------------------------------------GCAKM---TSKIGGYVGG-GGWK-YA------------------------HGTAH-------Y-GRNFSLKWLKLCELSFHK-TRHLRNPYNENL-PVKISRDCQELE-----------------------PSIGEQLASLL
	evm_27.model.AmTr_v1.0_scaffold00155.52	AtrYTHDC1A	AK-----------FFVIKSYSEDD--VHK--SIKYHVWSSTPSGNNRLNTA---FE--DAQRR-SGG--------------------------------------KPGSCP-----VFLFFS--VNASGQFC------------------------------------GVAEM---VGSVDFQKDM-DFWQ-QD------------------------------------KW-SGCFPVKWHFVKDVPNSY-FRHIILENNENK-PVTNSRDTQEIR-----------------------FPQGIEMLAIF
Glycine max	Glyma.01G214100.1	GmYTHDC1Ba	AL-----------FFVIKSYSEDD--IHK--SIKYNVWASTPNGNKRLDGA---FQ--DAQKR-MEE--------------------------------------KGCKCP-----VFLFFS--VNASGQFC------------------------------------GVAEM---TGRVDFNKSM-DFWQ-QD------------------------------------KW-NGYFPVKWHIIKDVPNPQ-LRHIILENNDHK-PVTSSRDTQEVS-----------------------FPQGVEMLNIF
	Glyma.02G072000.1	GmYTHDF5A	AK-----------FFVIKSYSEDD--VHK--SIKYNVWSSTPHGNKKLENA---YE--DAKKI-AAE--------------------------------------KSEVCP-----IFLLFS--VNASGQFC------------------------------------GVAEM---VGTVDFSKNM-DFWQ-QD------------------------------------KW-SGSFPVKWHIIKDVPNPN-FRHIILENNENK-PVTNSRDAQEIM-----------------------YLKGLEMLKIF
	Glyma.05G166600.1	GmYTHDF2A	SR-----------YFIVKSCNREN--LEL--SVQQGVWATQRSNESKLNEA---FD--SVEN---VILVFS--VNRTRHFQ------------------------------------GCAKM---TSRIGGSVAG-GNWK-YA------------------------HGTAH-------Y-GRNFSVKWLKLCELSFHK-TRHLRNPYNENL-PVKISRDCQELE-----------------------PSIGEQLASLL
	Glyma.07G000900.1	GmYTHDF2B	TK-----------YFVIKSLNHQN--IHL--SIEKGIWATQIMNEPILQEA---YH--NSGS---VILIFS--VNMSGSFQ------------------------------------GYAQM---MTSIGRGRD--NAWS-EG------------------------TGKSN-------PW-GRSFKVKWLCLNDLPFHK-TLHLKNPLNDYK-PVKISRDCQELS-----------------------PDIGLALCKLL
	Glyma.07G233400.1	GmYTHDF2C	AK-----------FFVIKSYSEDD--VHK--SIKYNVWSSTPHGN-KLENA---YE--DAKKI-AAE--------------------------------------KSEVCP-----IFLFFS--VNASGQFC------------------------------------GVAEM---VGTVDFNKNM-DFWQ-QD------------------------------------KW-SGSFPLKWHIIKDVPNPN-FRHITLENNENK-PVTNSRDTQEIM-----------------------YWKGLEMLKIF
	Glyma.08G124600.1	GmYTHDF1A	SR-----------YFIVKSCNREN--LEL--SVQQGVWATQRSNESKLNEA---FD--SVEN---VILIFS--VNRTRHFQ------------------------------------GCAKM---TSKIGGSVAG-GNWK-YA------------------------HGTAH-------Y-GRNFSVKWLKLCELSFHK-TRHLRNPYNENL-PVKISRDCQELE-----------------------PSIGEQLASLL
	Glyma.08G162200.1	GmYTHDF6C	AK-----------FFVIKSYSEDN--VHK--SIKYGVWASTPNGNRKLDDA---YR--QAMEK---------------------------------------QDACP-----IFLFFS--VNASAQFC------------------------------------GVAEM---VGPVNFDKSV-DFWQ-QD------------------------------------KW-SGQFPLKWHIIKDVPNSQ-FRHIILENNDNK-PVTNSRDTQEVK-----------------------LGQGVEMLTIF
	Glyma.08G226100.1	GmYTHDF1B	AL-----------FFVIKSYSEDD--IHK--SIKYNVWASTPNGNKRLDGA---FQ--DAQKR-MEE--------------------------------------KGCKCP-----VFLFFS--VNASGQFC------------------------------------GVAEM---TGRVDFNKSM-DFWQ-QD------------------------------------KW-NGYFSVKWHIIKDVPNPQ-LRHIILENNDHK-PVTNSRDTQEVS-----------------------FPQGVEMLNIF

	Glyma.09G022200.1	GmYTHDC1Ab	AK------------FFVIKSYSEDD--IHK--SIKYNVWASTQNGNKKLDAA---YH--EAQQK-P--GGCP-----VFLFFS--VNTSGQFV-------------------------------------GLAEM---IGPVDFNKSV-EYWQ-QD--------------------------------------KW-NGCFPLKWHVVKDVPNNL-LRHITLDNNENK-PVTNSRDTQEVM------------------------LEPGLKLIKIF
	Glyma.09G031200.1	GmYTHDF4C	AK------------FFVIKSYSEDD--VHK--SIKYNVWSSTPHGNKKLQSA---HE--DAKRI-ASG--KFGSCP-----IFLFFS--VNASGQFC-------------------------------------GVAEM---IGPVDFNKDM-DFWQ-QD--------------------------------------KW-SGSFPVKWYIIKDVSNAN-FRHIILENNENK-PVTNSRDTQEIM------------------------YSKGLEMLKIF
	Glyma.11G027800.1	GmYTHDC1Bb	AK------------FFVIKSYSEDN--VHK--SIKYGVWASTPNGNRKLDAA---YL--QAMEK---QDACP-----IFLFFS--VNASAQFC-------------------------------------GVAEM---VGPVNFDKSV-DFWQ-QD--------------------------------------KW-SGQFPVKWHIIKDVPNSQ-FRHIVLENNDNK-PVTNSRDTQEVK------------------------LTQGVEMLTIF
	Glyma.15G128500.1	GmYTHDC1Aa	AK------------FFVIKSYSEDD--IHK--SIKYNVWASTQNGNKKLDAA---YQ--EAQQK-P--GGTP-----VFLFFS--VNTSGQFV-------------------------------------GLAEM---IGPVDFNKSV-EYWQ-QD--------------------------------------KW-NGCFPLKWHIVKDVPNNL-LRHITLDNNENK-PVTNSRDTQEVM------------------------LEPGLKLIKIF
	Glyma.15G136400.1	GmYTHDF3C	AK------------FFVIKSYSEDD--IHK--SIKYKVWASTFNGNKKLDAA---YH--ESKEK-P--GDCP-----VFLLFS--VNTSGQFV-------------------------------------GLAEM---VSPLDFGRTV-EYWQ-QD--------------------------------------RW-SGCFSVKWHIIKDIPNSV-LRPITLENNENK-PVTNSRDTQEVK------------------------FEKGIQILKIF
	Glyma.15G265400.1	GmYTHDF5C	IK------------YFIIKSLNHQN--IHL--SIEKGIWATQIMNEPILEEA---FH--NSGS--VILIFS--VNMSGSFQ-------------------------------------GYAQM---MSSIGRGRD--NVWS-EG--------------------------TGKSN-------PW-GRSFKVKWMCLNDLPFHK-TLHLKNPLNDYK-PVKISRDCQELS------------------------PDIGLALCELL
	Glyma.16G003200.1	GmYTHDF7C	AK------------FYVIKSFNEDD--VHK--SVKYNVWTSTPNGNKKLNAA---FL--DAEAK-LRQ--TGTKCP-----VFLFFS--VNASRQFV-------------------------------------GVAEM---LGPVDFKNDM-NFWK-LD---------------------------------KY-NGFFPIKWHIIKDVPNNQ-FVHIILPSNENK-PVTYTRDTQEIG-------------------------LKEGLEMLNIF
	Glyma.16G041600.1	GmYTHDF4A	AK------------FFVIKSYSEDD--IHK--SIKYSAWASTPNGNKKLDSA---YQ--EAKEK-P---GGCP-----IFLLFS--VNTSGQFV-------------------------------------GLAEM---LGPVDFGKTV-DYWQ-QD--------------------------------------RW-TGCFSVKWHVIKDIPNSV-LRHITLENNENK-PVTNSRDTQEVK------------------------FEKGVQLVKIF
	Glyma.17G038400.1	GmYTHDF1C	AK------------FFVIKSYSEDD--VHK--SIKYNVWSSTPHGNKKLQST---HE--DAKRI-ASG--NFGSCP-----IFLFFS--VNASGQFC-------------------------------------GVAEM---IGPVDFNKDM-DFWQ-QD--------------------------------------KW-SGSFPVKWHIIKDVPNAN-FRHIILENNENK-PVTNSRDTQEIM------------------------YSKGLEMLKIF
	Glyma.19G110800.1	GmYTHDF3A	AK------------FFVIKSYSEDD--IHK--SIKYSAWASTPNGNKKLDAA---YQ--EAKEK-P--GGCP-----IFLLFS--VNTSGQFV-------------------------------------GLAEM---LGPVDFGKTV-DYWQ-QD--------------------------------------RW-TGCFSVKWHVIKDIPNSV-LRHITLENNENK-PVTNSRDTQEVK------------------------FEKGVQIAKIF
Gossipium raimondii	Gorai.002G031800.1	GrYTHDF2C	AK------------FFIIKSYSEDD--VHK--SIKYNVWASTSTGNKKLDAA---FH--ETKGN-P---DICP-----VFLLFS--VNTSGQFV-------------------------------------GLAEM---VGPVDFNKTV-EYWQ-QD--------------------------------------KW-TGCFPVKWHIIKDVPNTS-LRHITLENNENK-PVTNSRDTQEVN------------------------FEQGVQILKIF

	Gorai.002G182900.1	GrYTH DF3B	AK------------FFIIKSYSEDN--VHK--SIKYGVWASTPNGNKKLDAA---YR--EAKEN--QDPCP-----VFLFFS--VNASAQFC-----------------------------------GVAEM---VGPVDFDKSV-DYWQ-QD-------------------------------------KW-SGQFPVKWHFIKDVPNSQ-FRHIILENNDNK-PVTNSRDTQEVR-----------------------LEQGIEMLNIF
	Gorai.004G020600.1	GrYTH DF1A	AK------------FFIIKSYSEDN--VHK--SIKYGVWASTPNGNKRLDAA---YR--EAMEN---QDNCP-----VFLFFS--VNASAQFC-----------------------------------GVAEM---VGPVDFDKSV-DYWQ-QD-------------------------------------KW-SGQFPVKWHIIKDVPNSQ-LRHIILENNDNK-PVTNSRDTQEVK-----------------------LEQGLEMLSIF
	Gorai.004G205800.1	GrYTH DC1B	SR------------YFIVKSCNREN--LEL--SVQQGVWATQRSNEAKLNEA---FD--SAEN---VILVFS--VNRTRHFQV-----------------------------------GCAKM---TSKIGGSVAG-GNWK-YA--------------------------HGTAH--------Y-GRNFSVKWLKLCELSFHK-TRHLRNPYNENL-PVKISRDCQELE-----------------------PSVGEQLASLL
	Gorai.004G244600.1	GrYTH DF3A	AK------------FFVIKSYSEDD--VHK--SVKYNVWASTSNGNKKLDAA---FH--EAKEE-P---DGCP-----VFLLFS--VNTSGQFV-----------------------------------GVAEM---VDQVDFNKTV-EYWQ-QD-------------------------------------KW-TGCFPVKWHIIKDVPNTL-LRHITLENNENK-PVTNSRDTQEVN-----------------------FEQGIHILKIF
	Gorai.005G070500.1	GrYTH DF1C	AM------------FFVIKSYSEDD--VHK--SIKYNVWASTSNGNKKLDAA---FR--EAKEK-P--DGCP-----VFLLFS--VNTSGQFV-----------------------------------GLAEM---VGPVDFNKTV-EYWQ-QD-------------------------------------KW-TGCFPVKWHIVKDVPNAS-LRHITLKNNENK-PVTNSRDTQEVN-----------------------FEQGIQILKIF
	Gorai.005G265300.1	GrYTH DF3C	AK------------FFVIKSYSEDD--VHK--SMKYNVWSSTPNGNRKLDAA---FH--EAETR-ESE--------------------------------------TGTKCP-----IFLLFS--VNGSGQFV-----------------------------------GLAEM---IGKVDFNKDM-DFWQ-LN-------------------------------------KW-NGFFPVKWHVIKDIPNKE-LFHIILENNENK-PVTHSRDTQEIG-----------------------LKQGLELLRIF
	Gorai.007G288900.1	GrYTH DF2A	AK------------FFVIKSYSEDD--VHK--SIKYNVWSSTPHGNKKLDSA---FE--DAEKI-AAG-------------------------------------KPSGCP-----IFLFFS--VNASGQFC-----------------------------------GVAEM---IGPVDFQKDM-DFWQ-QD-------------------------------------KW-SGSFPVKWHIIKDVPNSH-FRHIILGNNENK-PVTNSRDTQEIM-----------------------YKQGMEMLKVF
	Gorai.007G289200.1	GrYTH DF4A	AK------------FFVIKSYSEDD--VHK--SIKYNVWASTPNGNRKLDAA---FC--EAKEK-P--DGCP-----IFLLFS--VNTSGQFV-----------------------------------GLAEM---VGAVDFNRTV-EYWQ-QD-------------------------------------KW-TGCFPVKWHIVKDVPNTS-LRHITLKNNENK-PVTNSRDTQEVN-----------------------FEQGMQILKIF
	Gorai.008G046800.1	GrYTH DC1A	KR------------YFIIKSLNHQN--IQL--SIDKGIWATQVMNEPILEDA---FH--NSGC--VILIFS--VNMSGFFQ-----------------------------------GYAQM---MSSVGWRRD--NVWS-QG--------------------------SGKGN-------PW-GRSFKVKWLCLNHLPFQK-TLHLKNPLNDYK-PVKISRDCQELP-----------------------QDIGEALCELL
	Gorai.009G120700.1	GrYTH DF2B	AK------------FFVIKSYSEDD--VHK--SIKYNVWASTPNGNKKLDAA---YQ--EAQQK-S---GHCP-----IFLFFS--VNTSGQFV-----------------------------------GIAEM---VGAVDFQKNV-EFWQ-QD-------------------------------------KW-TGCFPVRWLIIKDVPNNS-LKHITLENNENK-PVTNSRDTQEIK-----------------------LDQGLKLLKIF

	Gorai.011G001700.1	GrYTH DF1B	AK------------FFVIKSYSEDN--VHK--SIKYGIWASTPIGNKKLDTA---YH---EAKAI--QGTCP-----VFLLFS--VNSSAQFC------------------------------------GVAEM---VGPVDFDKSV-DYWL-QN-------------------------------------KW-SGQFPVKWHIIKDVPNSQ-FRHILLESNDNK-PVTNSRDTQEVE-----------------------FEQGIEMINIF
	Gorai.013G123200.1	GrYTH DF5A	AK------------FFVIKSYSEDD--VHK--SIKYNVWSSTPHGNKKLDSA---FE---DAQRI-AAG--KPRGCS-----IFLFFS--VNASGQFC------------------------------------GVAEM---IGSVDFQKAM-DFWQ-QD-------------------------------------KW-SGSFPVKWHIIKDVPNSH-FRHIIVENNENK-PVTNSRDTQEIM-----------------------FKQGVEMLKVF
Zea mays	GRMZM2G004997_T02	ZmYTH DF3B	AK------------FFVIKSYTEDH--VHR--SIKYNVWASTASGNRKLDSA---YR---AAREK---EEHCP-----IFLFFS--VNGSGQFC------------------------------------GVAEM---IGPVDFDRSV-NYWQ-QD-------------------------------------KW-SGQFPVKWHIIKDVPNNL-LRHIILENNDNK-PVTNSRDTQEVK-----------------------LEQGLQMLTIF
	GRMZM2G025488_T02	ZmYTH DF1B	AR------------FFIIKSYSEDN--VHK--SVKYGVWASTTNGNKKLDSA---YR---EAKEK---GEHCP-----IFLLFS--VNASAQFC------------------------------------GVAEM---IGPVDFEKSV-DYWQ-QD-------------------------------------KW-TGQFPVKWHIVKDVPNNL-FRHIILENNDNK-PVTNSRDTQEVK-----------------------LEQGMEMLKIF
	GRMZM2G056573_T01	ZmYTH DF3C	AK------------FFVIKSIGEAD--VHK--SIKYGVWSSSSNGNIKLDSA---FR---DADRI-SRR---HSTKCP-----VFLFFS--VNGSGHFC------------------------------------GMAEM---VGPVDFHKDM-DFWC-QD-------------------------------------KW-TGCFPVRWHIVKDIPNGF-LQHITLQNNENK-PVTHSRDTQEIP-----------------------YLPGISVIKIF
	GRMZM2G076062_T02	ZmYTH DF1A	AK------------FFVIKSYSEDD--IHK--SVKYNVWASTTNGNKKLDAA---YQ---EAQSK-G---SACP-----IFLFFS--VNTSGQFV------------------------------------GVAEM---TGAVDFEKTL-GYWQ-QD-------------------------------------KW-NGSFSVKWHIVKDVPNNI-LKHIILENNENK-PVTNSRDTQEIH-----------------------LEQGLQMLKIF
	GRMZM2G098174_T01	ZmYTH DF4C	AK------------FFVIKSIGEAD--VHK--SIKYGVWSSSSSGNSKLDSA---FR---DADRI-SRR---HSTKCP-----VFLFFS--VNGSGHFC------------------------------------GMAEM---VGPVDFHKDM-DFWC-QD-------------------------------------KW-TGCFPVRWHIVKDIPNGS-LQHITLQNNENK-PVTHSRDTQEVP-----------------------YLPGMSVINIF
	GRMZM2G102069_T01	ZmYTH DF4B	AR------------FYVIKSYTEDH--IHK--SIKYNVWASTPRGNRKLNAG---YH---EAKAK---EDHCP-----IFLFFS--VNSSGHFC------------------------------------GVAEM---IGPVNFDKSV-DYWQ-NE-------------------------------------RW-NGQFPVKWHIVKDVPNNI-VRHIILENNENK-RVTNSRDTQEVK-----------------------LKQGLQMLAIF
	GRMZM2G126338_T01	ZmYTH DF3A	AK------------FFVIKSYSEDD--VHK--SIKYNVWASTPNGNKKLDAG---YR---EAQEK-S---SDCP-----VFLFFS--VNTSGQFV------------------------------------GVAEM---VGPVDFDKTV-EYWQ-QD-------------------------------------KW-NGCFPLKWHIVKDVPNNT-LKHITLDNNDNK-PVTNSRDTQEVK-----------------------LEQGLEMLKIF
	GRMZM2G127780_T01	ZmYTH DF2C	AL------------FFVIKSYSEDD--IHK--SVKYNVWASTPNGNKRLDNA---YR---VAQER-IAE--KGTKCP-----VFLFFS--VNASGQFC------------------------------------GVAEM---VGPVDFNRNM-NFWQ-QD-------------------------------------KW-NGFFSVKWHIIKDVPNPQ-FRHIILENNENK-PVTNSRDTQEVN-----------------------FSQGTEMLNIF
	GRMZM2G144726_T01	ZmYTH DF4A	AK------------FFIIKSYSEDD--VHK--SVKYNVWASTPNGNKKLDAA---YQ---EAKEK-S---SETP-----VFLLFS--VNASGQFV------------------------------------GLAEM---

			VGRVDFEKTV-EHWQ-QD--------------------------------------KW-TGCFPVKWHIVKDVPNSL-LKHIILENNENK-PVTNSRDTHEVK------------------------ LEQGLQVLKIF
	GRMZM2G169116_T01	ZmYTHDF1C	AL------------FFVIKSYSEDD--IHK--SIKYNVWASTPNGNKRLDNA---YR--LAQER-MAE--KGTKCP-----VFLFFS--VNASGQFC--------------------------------------GVAEM---VGPVDFNRNM-NFWQ-QD--------------------------------------KW-NGFFSVKWHIIKDVPNPQ-FRHIILENNENK-PVTNSRDTQEVK------------------------ FSQGTEMLNIF
	GRMZM2G303312_T01	ZmYTHDF5C	AK------------FFMIKSYSEDD--IHK--GIKYNVWASTPNGNNKLDAA---YH--EAQNL-M-D---SGERCP-----VFLFFS--VNTSGQFV--------------------------------------GLAEM---LGPVDFKKTM-DFWE-ED--------------------------------------KW-SGFFPIKWHIIKDIPNRL-FKHIILENNDNR-IVTFSRDTQEIG------------------------LPQGVQMLKIF
	GRMZM2G330019_T01	ZmYTHDF2A	AK------------FFVIKSYSEDD--IHK--SVKYNVWASTTNGNKKLDAA---YQ--EAQSK-G---SACP-----IFLFFS--VNTSGQFV--------------------------------------GVAEM---TGAVDFEKTL-EYWQ-QD--------------------------------------KW-NGSFSVKWHIVKDVPNNI-LKHIILENNENK-PVTNSRDTQEIR------------------------ LEQGLQMLKIF
	GRMZM2G340130_T01	ZmYTHDF5A	AK------------FFIIKSYSEDD--VHK--SVKYNVWASTPNGNKKLDAA---YQ--EAKEK-S---SETP-----VFLLFS--VNASGQFV--------------------------------------GLAEM---VGRVDFNKTV-DHWQ-QD--------------------------------------KW-TGCFPVKWHIVKDVPNSL-LKHIILENNENK-PVTNSRDTHEVK------------------------ LEQGLQVLKVF
	Zm00008a020283_T01	ZmYTHDC1A	AK------------FFVIKSYTEDH--AHR--SIKYKVWASTASGNRKLDSS---YH--AAKEK---EEHCP-----IFLFFS--VNGSGQFC--------------------------------------GMADM---IGSVDFDRSV-DYWQ-QN--------------------------------------KW-SGQFPVKWHIIKDVPNNL-LWHIILENNDNK-PVTNSRDTHEVS------------------------LERPLCY----
	GRMZM2G442195_T01	ZmYTHDF2B	AK------------FFVIKSYTEDH--VHR--SIKYKVWASTASGNRKLDSA---YH--AAKEK---EEHCP-----IFLFFS--VNGSGQFC--------------------------------------GVAEM---IGPVDFDRSV-DYWQ-QD--------------------------------------KW-SGQFPVKWHIIKDVPNNL-LRHIILENNDSK-PVTNSRDTQEVK------------------------LEQGLQMLTIF
	GRMZM2G363789	ZmYTHDF5B	AK------------FFVIKSYTEDH--AHR--SIKYKVWASTASGNRKLDSS---YH--AAKEK---EEHCP-----IFLFFS--VNGSGQFC--------------------------------------GMADM---IGSVDFDRSV-DYWQ-QN----------------------------------KW-SGQFPVKWHIIKDVPNNL-LWHIILENNDNK-PVTNSRDTHEVS------------------------LERPLCY
Musa acuminata	GSMUA_Achr1T10560_001	MaYTHDF5B	GK------------IFIIKSYSEAN--VHK--SIKYGVWSSTSAGNKKLNSA---YI--EAQKK--GHSSP-----IFLFFS--VNASGRFC--------------------------------------GVAEM---IGPVDFEKSV-DYWN-KD--------------------------------------KW-TGQFPVKWHIVKDVPSFR-FRHIILENNDNK-PVTNSRDTQEVK------------------------LEQGLEMLSIF
	GSMUA_Achr1T10590_001	MaYTHDF4B	AR------------FFIIKSYSEDN--VHK--SIKYGVWASTSNGNRKLNSA---YI--EMREQ--EHSYP-----IFLFFS--VNASGHFC--------------------------------------GVAEM---IGPVDFEKSV-DYWN-ED--------------------------------------KW-TGQCPVKWHIVKDVPNTM-FRHIILEKNDNK-PVTNSRDTQEVK------------------------LEQGLEMLGIF
	GSMUA_Achr1T24980_001	MaYTHDF3C	AR------------FFVIKSYSEDD--VHK--SIKYNVWSSTHNGNRRLDGA---YE--DAQSK-SVG---RPRKCP-----VFLFFS--VNASGQFC--------------------------------------GLAEM---VGPVDFDKDM-NFWQ-QD--------------------------------------KW-SGSFPVKWHIIKDVPNSS-LRHIILENNENK-PVTNSRDTQEIP------------------------ YSAGIDMLKIF

	GSMUA_Achr4T04600_001	MaYTHDF1B	AK------------FFIIKSYSEDN--VHK--SIKYGVWASTSNGNKKLDSA---YH--EAKKK---EDPCP-----VFLFFS---VNASAHFC-------------------------------------GVAEM---IGPVDFERSV-DYWQ-QD-------------------------------------KW-SGQFPLKWHMVKDVPNNL-FRHIILENNENK-PVTNSRDTQEVK-----------------------LEQGLEMLSIF
	GSMUA_Achr4T11560_001	MaYTHDF2B	AK------------FFVIKSYSEDN--VHK--SIKYGVWASTSNGNKKLDSA---YH--EANKK---EDPCP-----VFLFFS---VNASAHFC-------------------------------------GVAEM---IGPVDFEKSV-DYWQ-QD-------------------------------------KW-SGQFPVKWQIVKDVPNNL-FRHIILENNDNK-PVTNSRDTQEVK------------------------LEQGLEMLSIF
	GSMUA_Achr4T12480_001	MaYTHDF3B	AM------------FFIIKSYSEDN--VHK--SIKYGVWASTSSGNRKLDSA---YH--EAKQK---EDPCP-----VFLFFS---VNASAHFC-------------------------------------GVSEM---IGPVDFEKSV-DYWQ-QD-------------------------------------RW-SGQFPVKWHMVKDVPNNL-FRHIILENNENK-PVTNSRDTQEVK-----------------------LEKGLEMLGIF
	GSMUA_Achr4G14820_001	MaYTHDF7A	AK------------LFVIKAYSEDD--IHK--SIKYNVWASTGYGNKKLDAG---YQ--EAQGK---AGGCP-----VLLFFS---VNTSGQFV-------------------------------------GVAEM---DVPNSSILKHITLENNDNK-PVTNSRDAQEVM-----------------------LEQGLRMLKIF
	GSMUA_Achr4T21380_001	MaYTHDF1A	AK------------FFIIKSYSEDD--IHK--SIKYNVWASTPHGNKKLDAA---YQ--ESKEK-T--SGCP-----VFLFFS---VNTSGQFV-------------------------------------GVAEM---VGPVDFNKTL-DYWQ-QD-------------------------------------KW-VGYFPVKWHIVKDVPNNI-LKHITLENNDNK-PVTNSRDTQEVK-----------------------LDQGLQLLKLF
	GSMUA_Achr4T22630_001	MaYTHDF2A	AK------------FFIIKSYSEDD--IHK--SIKYNVWASTPHGNKKLDAA---YQ--ESKEK-T--NGCP-----VFLFFS---VNTSGQFV-------------------------------------GVAEM---VGRVDFNKTL-DYWQ-QD-------------------------------------KW-IGCFPVKWHIVKDVPNNI-LKHITLENNENK-PVTNSRDTQEVK-----------------------LEQGLQLLKLF
	GSMUA_Achr4T28430_001	MaYTHDF4A	AK------------FFIIKSYSEDD--IHK--SIKYNIWSSTPHGNKKLDAA---YQ--ESKEQ-T---SGCP-----IFLFFS---VNTSGQFV-------------------------------------GVAEM---IGRVNFNRTF-GYWQ-QD-------------------------------------KW-IGCFPVKWQIVKDVPNNI-LKHIILEDNENK-PVTNSRDTQEVK-----------------------LDQGLQLLRLF
	GSMUA_Achr5T02830_001	MaYTHDF3A	AK------------FFIIKSYSEDD--IHK--SIKYSIWASTPHGNKKLDAG---YQ--ESKQK-T---SGCP-----VFLFFS---VNTSGQFV-------------------------------------GVAEM---VGQVNFNKTL-DYWQ-QD-------------------------------------KW-VGCFPVKWHIVKDVPNSV-LKHITLENNDNK-PVTNSRDTQEVK-----------------------LDQGLQLLKLF
	GSMUA_Achr6T12810_001	MaYTHDF5C	AK------------FFMIKSYNEDD--VHK--SVKYNVWASTPNGNKKLDAV---FW--DAERL-----M-------------------------------------KKGSKCP-----IFLFFS---VNASGQFV-------------------------------------GLAEM---IGPVDFNKNL-DFWQ-KE-------------------------------------TW-NGFFPVKWHIIKDIPNRL-FQSIRLENNDNK-AVTFSKDTQEVSFSLSCIERFPIMQVLLGQQFTVIGLPQGLQMLHIF
	GSMUA_Achr6T19820_001	MaYTHDC1Ab	SR------------YFIVKSCNREN--LEI--SVQQGVWATQRSNEPKLNEA---FE--STEN---VILIFS---INKTRHFQ-------------------------------------GCAKM---TSRIGGLVGG-GNWK-YS--------------------------HGTAH--------Y-GRNFSVKWLKLCELSFNK-THHLRNPYNDNL-PVKISRDCQELE-----------------------PFIGEQLASLL
	GSMUA_Achr6T26690_001	MaYTHDF2C	AL------------FFVIKSYSEDD--IHK--SIKYSVWASTPNGNKRVDNA---FQ--VAQEK-MTE--KGGNCP-----VFLFFS---VNASGQFC-------------------------------------GVAEM---TGRVDFSKNM-DFWQ-QD-------------------------------------KW-NGFFPVKWHIIKDVPNLQ-FRHIILENNDNK-PVTNSRDTQEVK-----------------------FSQGIEMLSIF

	GSMUA_Achr7T01490_001	MaYTHDF5A	AK------------FFIIKSYSEDD--IHK--SIKYNVWASTLHGNKKLDAA---YL--ESKEK-T--SGCP-----IFLFFS---VNTSGQFV-----------------------------------GVAEM---VGPVDFNKTL-DFWQ-QD-------------------------------------KW-IGCFPVKWHIVKDVPNSI-LKHIKVENNDNK-PVTNSRDTQEVK-----------------------LEQGLQLLKLF
	GSMUA_Achr7T27220_001	MaYTHDF6A	AR------------FFVIKSYSEDD--IHK--SIKYNVWTSTPNGNKKLDGG---YQ--EAQEK-D---GGCP-----VFLFYS---VNASGQFV-----------------------------------GVAEM---VGPVDFNKTV-DYWQ-QD-------------------------------------KW-IGCFPVKWHIIKDVPNSL-LKHITLENNDNK-PVTNSRDTQEVR-----------------------LEQGLQMLKIF
	GSMUA_Achr8T29890_001	MaYTHDF4C	AK------------FFVIKSYSEDD--VHK--SIKYNVWSSTPNGNKRLNSA---YE--DAQRK-SAG---------------------------------------KSRKCP-----VFLFYS---VNASGQFC-----------------------------------GLAEM---VGPVDFDQDM-DFWQ-QD-------------------------------------KW-SGSFPVKWHVIKDVSNAS-LRHIILENNENK-PVTNSRDTQEIP-----------------------YSTGINMLSIF
	GSMUA_Achr9T06180_001	MaYTHDF1C	AL------------FFVIKSYSEDD--IHK--SIKYNVWASTPNGNKRLDNA---FQ--VAQEK-MAE---------------------------------------KNSKCP-----VFLFFS---VNASGQFC-----------------------------------GVAEM---TGRVDFSKDM-DFWQ-QD-------------------------------------KW-NGFFPVKWHIIKDVPNPQ-FRHIILENNDNK-PVTNSRDTQEVK-----------------------FSQGTEMLSIF
	GSMUA_Achr9T30440_001	MaYTHDC1Aa	SRCLSCMNAVLSIMYFIVKSCNREN--LEI--SVQQGMWATQRSNEAKLNEA---FE--STEN--VILIFS---INKTRHFQ-----------------------------------GCGKM---TSRIGGFVGG-GNWK-YS-------------------------HGTAH--------Y-GRNFSVKWLKLCELSFNK-THHLRNPYNDNL-PVKISRDCQELE-----------------------PFIGEQLASLL
Vitis vinifera	GSVIVT01010494001	VvYTHDF2B	AF------------FFVIKSYSEDD--IHK--SIKYNVWASTPNGNKRLDGA---YQ--EAKER-MGD---------------------------------------RGSKCP-----VFLFFS---VNASGQFC-----------------------------------GVAEM---IGRVDFNKNM-DFWQ-QD-------------------------------------KW-NGFFPVKWHIIKDVPNPQ-LRHIILENNDNK-PVTNSRDTQEVR-----------------------FPQGIEILNIF
	GSVIVT01017649001	VvYTHDF1C	AK------------FFIIKSYSEDD--VHK--SIKYNMWASTANGNKKLDAA---YQ--EAQGK-S---GSCP-----IFLLFS---VNASGQFV-----------------------------------GVAEM---VGSVDFNRSL-EYWQ-QD-------------------------------------KW-TGCFPVKWHVIKDIPNSL-LKHITLENNENK-PVTNSRDTQEVK-----------------------FEQGIQVLKIF
	GSVIVT01019895001	VvYTHDC1B	AK------------FFIIKSYSEDN--VHR--SIKYSVWASTALGNRKLDAA---YQ--KAKEI---ETNCP-----IFLCFS---VNASSQFC-----------------------------------GVAEM---VGPVNFEKDA-EYWQ-QD-------------------------------------RW-SGLFPVKWHIIKDVPNSL-FRHILLENNENK-PVTHSRDTQEVN-----------------------LEQGIEMLKIF
	GSVIVG01021617001	VvYTHDC1A	AK------------FFIIKSYSEDN--VHK--SIKYGVWASTPNGNRKLDAA---YR--EAKEK---QAACP-----VFLLFS---VNASAQFC-----------------------------------GVAEM---VGPVDFDKSV-DYWQ-QD-------------------------------------KW-SGQFPVKWHIIKDVPNSQ-FRHIVLENNDNK-PVTNSRDTQEVK-----------------------LEQGIEILNIF
	GSVIVT01023248001	VvYTHDF3C	AK------------FFIIKSYSEDD--VHK--SIKYNVWASTPNGNKKLDAA---YR--EAQEK-S--AGCP-----VFLFFS---VNTSGQFV-----------------------------------GLAEM---VGHVDFHKNV-EYWQ-QD-------------------------------------KW-NGCFSVKWHVVKDVPNSL-LKHITLENNENK-PVTNSRDTQEVK-----------------------LEQGLQMLKIF

	GSVIVT01025494001	VvYTH DF1A	AK------------FYVIKSFSEDD--IHK--CIKYDVWASTPNGNKKLDAA---FH--DAEAK-ANE--------------------------------------TGTKFP-----IFLFFS--VNGSGQFV------------------------------------GVAEM---VGQVDFNKDM-DFWQ-LD-------------------------------------KW-NGFFPVKWHIVKDIPNSQ-LRHITLESNENR-SVTYTRDTQEIG-----------------------LKQGVEMLKIF
	GSVIVT01029460001	VvYTH DF1B	SR------------YFIVKSCNREN--LEL--SVQQGVWATQRSNEAKLNEA---FD--SVEN--VILIFS--VNRTRHFQ------------------------------------GCAKM---TSKIGGFVGG-GNWK-YA-------------------------HGTAH--------Y-GRNFSVKWLKLCELSFHK-TRHLRNPYNENL-PVKISRDCQELE-----------------------PSIGEQLASLL
	GSVIVT01032236001	VvYTH DF3B	TR------------YFIIKSLNHHN--IQL--SMEKGIWATQVMNEPILEEA---FH--NSGK--VILIFS--VNMSGFFQ------------------------------------GYAQM---MSSVGWRRD--NVWS-QG-------------------------SGGNN-------PW-GRSFKVKWLRLHDLPFQK-TLHLKNPLNDYK-PVKISRDCQELS-----------------------QEIGEALCELL
	GSVIVT01033395001	VvYTH DF2A	AK------------FFVIKSYSEDD--VHK--SIKYNVWSSTPNGNKKLENA---YE--DAQRI-ALG---------------------------------------KRRGCP-----IFLFFS--VNASGQFC------------------------------------GVAEM---IGPVDFHKDM-DFWQ-QD-------------------------------------KW-SGSFPVKWHIIKDVPNTN-FRHIILENNENK-PVTNSRDTQEIR-----------------------FKQGLEMLKIF
	GSVIVT01036696001	VvYTH DF2C	AK------------FFVIKSYSEDN--VHK--SIKYGVWVSTPNGNKRLNSA---FH--EAKEK---HGNCP-----IFLLFS--VNASAQFC------------------------------------GVAEM---VGPVDYDKSV-DYWK-QD-------------------------------------KW-TGQFPVKWHIIKDVPNSQ-FRHIILENNDNK-PVTNSRDTQEVE-----------------------LEQGNEMLNIF
Manihot esculenta	Manes.02G029400.1	MeYTH DC1B	AF------------FFVIKSYSEDD--IHK--SIKYNVWASTPNGNKRLDSA---YQ--DAQKK-IAE---------------------------------------KGSNCP-----VFLFFS--VNASGQFC------------------------------------GVAEM---IGQVDFNKNM-DFWQ-QD-------------------------------------KW-NGYFPVKWHIIKDVPNPQ-LRHIILENNENK-PVTNSRDTQEVK-----------------------FPQGMEMLNVF
	Manes.06G172700.1	MeYTH DF3A	AK------------FFVIKSYSEDD--VHK--SIKYNVWSSTPNGNKKLQSA---YD--DAQKI-AAG--------------------------------------DAKACP-----IFLFFS--VNASGQFC------------------------------------GVAEM---IGPVDFAKDM-DFWQ-QD-------------------------------------KW-SGSFPVKWHIIKDVPNSS-FRHIILENNENK-PVTNSRDTQEIM-----------------------YKQGLEMLKIF
	Manes.07G024100.1	MeYTH DF3C	SR------------YFIVKSCNREN--LEL--SVQQGVWATQRSNEAKLNEA---FD--SAEN--VILIFS--VNRTRHFQ------------------------------------GCAKM---TSKIGASAVG-GNWK-YA-------------------------HGTAH--------Y-GRNFSVKWLKLCELSFHK-TRHLRNPYNENL-PVKISRDCQELE-----------------------PSIGEQLASLL
	Manes.07G081200.1	MeYTH DF2A	AK------------FFVIKSYSEDD--VHK--SIKYNVWASTPNGNKKLDAG---YQ--EAQQK-S---GGCP-----VFLFFS--VNTSGQFV------------------------------------GLAEM---IGPVDFHKNV-EYWQ-QD-------------------------------------KW-TGCFPLKWHIVKDVPNSL-LKHITLENNENK-PVTNSRDTQEVK-----------------------MEQGLKMIKIF
	Manes.09G141700.1	MeYTH DF5A	AK------------FFIIKSYSEDN--VHK--SIKYGVWASTPNGNRRLDAA---YS--EAKEK---QVPCP-----VFLFFS--VNASAQFC------------------------------------GVAEM---VGPVDFDKSV-DYWQ-QD-------------------------------------KW-SGQFPVKWHIAKDVPNSQ-FRHIVLENNDNK-PVTNSRDTQEVK-----------------------LEQGIEMLNIF

	Manes.10G067800.1	MeYTHDF4A	AK------------FFVIKSYSEDN--VHK--SIKYGVWASTPNGNKKLDAA---YH--EAKEK---TGTSP-----IFLLFS---VNASAQFC---------------------------------------GVAEM---VGAVDFDKSV-DYWQ-QD--KW-SGQFPVKWHIIKDVPNSQ-FRHIVLENNDNK-PVTNSRDTQEVE-------------------------LEHGVEMLKIF
	Manes.12G010500.1	MeYTHDF3B	AK------------FFVIKSYSEDN--VHK--SIKYGVWASTLNGNKKLDGA---YH--EAKER---HGTSP-----VFLLFS---VNASAQFC---------------------------------------GVAEM---VGPVDFDKSV-DYWQ-QD--KW-SGQFPVKWHIIKDVPNSQ-FRHIVLENNDNK-PVTNSRDTQEVE-------------------------LEHGVEMLKIL
	Manes.13G011300.1	MeYTHDF2B	AK------------IFVIKSYSEDD--VHK--SIKYNVWASTPNGNKKLDAA---YQ--EAQQK-S--SGCP-----VFLFFS---VNTSGQFV---------------------------------------GLAEM---VGPVDFHKNV-EYWQ-QD--KW-TGCFPLKWHIVKDIPNSL-LKHITLENNENK-PVTNSRDTQEVK-------------------------LEQGLKMFKIF
	Manes.14G000200.1	MeYTHDF1B	AK------------FFIIKSYSEDD--VHK--CIKYGVWASTPNGNKKLDAA---YH--EAKET-P---GSCP-----VFLLFS---VNASGQFV---------------------------------------GLAEM---IGAVDFNKTV-GYWQ-QE--KW-IGCFPVKWHFIKDVPNSS-LRHITLENNENK-PVTNSRDTQEVM-------------------------LDKGIQILKIF
	Manes.14G012900.1	MeYTHDF1A	AK------------FFVIKSYSEDD--VHK--SIKYNVWASTPNGNKKLDAA---YQ--EAQQK-S---CGCP-----MFLFFS---VNTSGQFV---------------------------------------GLAEM---VGPVDFHKNV-EYWQ-QD--KW-TGCFPLKWHIVKDIPNSL-LKHITLENNENK-PVTNSRDTQEVK-------------------------LEQGLKMVKIF
	Manes.14G122500.1	MeYTHDC1A	AK------------FYVIKSYNEDD--IHK--SIKYDVWASTPNGNKKLDAA---FR--DAEQR-SDE--TGSKCP-----IFLFFS---VNGSGQFV---------------------------------------GIAEM---VGQVDFDKDM-DFWQ-LD--KW-NGFFPVKWHVIKDIPNSH-LRHIILENNERR-PVTFSRDTQEIG-------------------------LKQGLEMLNIF
	Manes.15G003300.1	MeYTHDF1C	AK------------FFVIKSYSEDD--VHK--SIKYNVWASTPNGNKKLDAA---YQ--EAQQK-P---GGCP-----VYLFFS---VNTSGQFV---------------------------------------GLAEM---VGPVDFHKNV-EYWQ-QD--KW-TGCFPLKWHIVKDIPNSL-LKHITLENNENK-PVTNSRDTQEVK-------------------------LEQGLKMIKIF
	Manes.16G048600.1	MeYTHDF2C	TR------------YFIIKSLNHHN--IQL--SVEKGVWATQVMNEPILEEA---FH--NSGK--VILIFS---VNMSGFFQ---------------------------------------GYAQM---ISSVGWRRD--NIWS-QG--------------------------CGKSN-------PW-GRSFKVKWLRLNDLPFQK-TLHLKNPLNDYK-PVKISRDCQELP-------------------------EDVGEALCDLI
	OAY33214	MeYTHDF4B	AR------------FFIIKSFSEDN--VHK--SIKYSVWASTPHGNKKLDVA---YR--EAKES--GSNCP-----VFLLFS---VNASGQFC---------------------------------------GVAQM---VGPVNFEKDA-DYWQ-QD------------------------------------RW-SGQFPVQWHIIKDVPNSR-FRHILLKNNDNK-PVTHSRDAQEVK--------------------------LEQGIEMLKIF
Marchantia polymorpha	Mapoly0007s0039.1	MpoYTHDC1B	AK------------FFVIKSYSEDD--VHK--SIKYNVWASTPNGNKRLEGA---YQ--DAQTR---Q-GPCP-----VFFFFS---VNASGQFC---------------------------------------GVAEM---TGPVDFNKSV-DYWQ-QD--KW-NGRFPVKWHIIKDIPNCQ-FRHIILENNDNK-PVTNSRDTQEVK-------------------------FEQGVEMLNIF

	Mapoly0056s0030.1	MpoYTHDC1A	CR------------FFIVKSSNREN--LEL--SVERGLWATHRNNESKLNEA---YD---SCDH---VILIFS---VNETRHFQ------------------------------------GCARM---MSKIGGVAGG-GGWK-YA-------------------------HGTAH--------Y-GRNFRLKWLKLCELSFNK-TRHLRNPFNENL-PVKISRDCQELE-----------------------PGVGEQLAQLL
	Mapoly0103s0072.1	MpoYTHDF1	TR------------YFIIKSLNHHN--IAK--SIENGIWATQAMNEPVLNEA---YE---SSER--VLLVFS---VNMSGHFQ------------------------------------GYAQM---ASPIGRRRA--NVWT-EG-------------------------NEGTN-------PW-GGSFRVEWLRLHDLPFQK-TVHLKNPLNFHK-PVKISRDCQELT-----------------------LEIGDALCALI
Medicago truncatula	Medtr2g005840.1	MtYTHDF4C	DEFAFTVCRDQYNLPDFQTKYETAKFYMIKSFNEDDIHKGIKYDVWTSTPHGNKKLNAAFQNAEAKLSQTGTQCPIFLFFSVNASGQFVGVAEMLGPVDFKKD MKFWKLDKYNGFFPIKWHIIKDVPNRQFAHITLQINENKCVTFSRDTQEITLKEGLEMLKIFKSYPAKTSLLDDFDFYENREKLCRSQRTEHTGW
	Medtr2g029540	MtYTHDC1A	AK------------FFVIKSYSEDD--IHK--SIKYNVWASTQNGNKKLDAA---YQ---EAQQK-S---GGCP-----IFLLFS---VNTSGQFV------------------------------------GLAEM---TGPVDFNKSL-EYWQ-QD----------------------------------KW-MGCFPLKWHIVKDVPNNV-LRHITLENNENK-PVTNSRDTQEIM-------------------------LEPGLKLLKIF
	Medtr2g032660	MtYTHDF3C	AK------------FFIIKSYSEDD--VHK--SIKYSVWASTPNGNKKLDAA---YQ---EA---GGCP-----IFLLFS---VNTSGQFV------------------------------------GLAEM---TGPVDFDKTV-EYWQ-QD----------------------------------RW-TGCFNVKWHIIKDIPNGV-LRHITLENNENK-PVTNSRDTQEVK------------------------FEKGVQIVKIF
	Medtr2g063460	MtYTHDF2C	AK------------FFVIKSYSEDD--IHK--SMKYNVWTSTPNGNKKLDAA---YL---EAKEK-S---ADCP-----IFLLFS---VNTSGQFV------------------------------------GLAEM---VSPVDFDRTV-EYWQ-QD----------------------------------RW-TGCFSVKWRIIKDIPNNV-LRHITLENNENK-PVTNSRDTQEVK------------------------FEKGVEILKIF
	Medtr4g005110	MtYTHDF1B	AK------------FFVIKSYSEDD--VHK--SIKYNVWSSTPHGNKKLQSA---YE---DARRK-ATG--KSGGCP-----IFLFFS---VNASGQFC------------------------------------GVAEM---VGPVDFNKDM-DFWQ-QD----------------------------------KW-SGSFPVKWHIIKDVPNGN-FRHIILENNEFK-PVTNSRDTQEIM------------------------YRKGLEMLKIF
	Medtr4g123630	MtYTHDF1C	AK------------FFVIKSYSEDN--VHK--SIKYGVWASTPNGNRKLDAA---YC---QAKEK--QDACR-----IFLFFS---VNASAQFC------------------------------------GVAEM---VGPVNFDKSV-DFWQ-QD----------------------------------KW-SGQFPVKWHIIKDVPNSQ-FRHIVLENNDNK-PVTNSRDTQEVK------------------------LQQGIEMLTIF
	Medtr6g032970	MtYTHDF3A	AL------------FFVIKSYSEDD--VHK--SIKYDVWASTPNGNKRLDNA---FQ---DAQNR-MEE--KGSKCP-----VFLFFS---VNASGQFC------------------------------------GVAEM---IGRVDFNKSM-DFWQ-QD----------------------------------KW-NGYFPVKWHIIKDIPNPQ-LRHIILENNDHK-PVTNSRDTQEIH------------------------FPQGIEMLNIF
	Medtr7g085500	MtYTHDF2A	AK------------FFVIKSYSEDD--VHK--SIKYNVWSSTVHGNRKLGNA---YE---DTKKV-SAE--KSGVCP-----IFLFFS---VNASGQFC------------------------------------GVAEM---IGSVDFNKDM-DFWQ-QD----------------------------------KW-SGSFPVKWHIIKDVPNPN-FRHIILDNNENK-PVTNSRDTQEIM------------------------YSKGLEMLKIF
	Medtr8g088390	MtYTHDF1A	NRTSTPLPQGIS-RYFIVKSCNREN--LEL--SVQQGVWATQRSNESKLNEA---FD---SVEN--VILIFS---VNRTRHFQ------------------------------------GCAKM---TSRIGGSVAG-GNWK-YA-----------------------HGTAH--------Y-GRNFSVKWLKLCELSFHK-TRHLRNPYNEN-LPVKISRDCQELE------------------------PSIGEQLASLL
Mimulus guttatus	Migut.A00893.1	MgYTHDC1B	AK------------FFVIKSYSEDD--VHK--SIKYNVWSSTPNGNKKLTTA---YQ---DAHRT-TLG--DLRGCP-----IFLFFS---VNASGQFC------------------------------------GVAEM---TGPVDFHRDM-DFWQ-QD----------------------------------KW-SGSFPVKWHIIKDLPNSN-FRHIILENNENK-PVTNSRDTQEIC------------------------YEKGLEMLRIY

	Migut.B00769.1	MgYTHDF4C	AK------------FYVIKSYSEDD--VHK--CIKYDVWSSTPNGNQKLDAA---FS---EAGAK-TRE-------------------------------------TGVNCP-----VFLFFS--VNGSGQFV-------------------------------------GVAEM---IGQVDFSKNM-DFWQ-LD-----------------------------------KW-NGFFPIKWHMIKDVPNTL-LRHIILENNENR-PVTYSRDTQEIG-------------------------LKQGLEMLRIF
	Migut.D02575.1	MgYTHDF2A	AR------------FFIIKSYSEDD--VHK--SIKYNVWASTPNGNKKLDAA---YQ---DAQQR-P--GGCP-----VFLFFS--VNTSGQFV-------------------------------------GVAEM---TGPVDFNKNV-EYWQ-QD-----------------------------------KW-VGCFPVKWHIVKDLPNSL-LKHITLENNENK-PVTNSRDTQEVK-------------------------LDQGLQVLKIF
	Migut.G00015.1	MgYTHDF2C	SR------------YFVVKSCNNEN--LEL--SVQQGVWATQRSNEAKLNEA---FE---SVDN--IILIFS--VNKTRHFQ-------------------------------------GCAKM---TSRIGGSISG-GNWK-NA-----------------------HGTAH--------Y-GQNFSVKWLKLGELSFNK-TRHLRNPFNENL-PVKISRDCQELE-------------------------PSIGEQLASLL
	Migut.H01337.1	MgYTHDC1Aa	AK------------FFVIKSFSEDN--IHK--SIKYSVWASTPLGNRKLDAA---YC---ETKEM---EGICP-----VFLLFS--VNASGQFC-------------------------------------GVAEM---IGPVDFENDA-DYWQ-QD-----------------------------------RW-SGQFPVKWHIIKDVPNSR-FRHILLENNENK-PVTHSRDSQEVK-------------------------LEQGIEMLQIF
	Migut.J00855.1	MgYTHDF1B	SR------------YFVVKSCNREN--LEL--SVQQGVWATQRSNEAKLNEA---FE---SVEN--IILIFS--VNKTRHFQ-------------------------------------GCAKM---TSRIGGSVGG-GNWK-HA-----------------------HGTAH--------Y-GRNFALKWLKLCELTFDK-TRHLRNPYNENL-PVKISRDCQELE-------------------------PSIGEQLASLL
	Migut.M01886.1	MgYTHDC1Ab	AK------------FFVIKSYSEDD--VHK--SIKYNVWSSTPNGNKKLTTA---YQ---DAHRT-GLG--DLRGCP-----IFLFFS--VNASGQFC-------------------------------------GVAEM---TGPVDFHRDM-DFWQ-QD-----------------------------------KW-SGSFPVKWHIIKDLPNSN-FRHIILENNENK-PVTNSRDTQEIC-------------------------YKKGLEMLRIY
	Migut.N00538.1	MgYTHDF1A	AK------------FFIIKSYSEDD--VHK--SIKYNIWASTSNGNKKLEAA---YQ---DAQQK-P---GGCP-----VFLFFS--VNTSGQFV-------------------------------------GVAEM---VGPVDFNKNV-EYWQ-QD-----------------------------------KW-IGCFPLKWHIVKDLPNSS-LKHITLENNENK-PVTNSRDTQEVK-------------------------LEPGLAVLKIF
	Migut.N00839.1	MgYTHDF3C	AK------------FYVIKSYSEDD--VHK--CIKYDVWSSTPNGNQKLDAA---FS---EAGAK-TRE-------------------------------------TGVNCP-----VFLFFS--VNGSGQFV-------------------------------------GVAEM---IGQVDFSKNM-DFWQ-LD-----------------------------------KW-NGFFPIKWHMIKDVPNTL-LRHIILENNENR-PVTYSRDTQEIG-------------------------LKQGLEMLRIF
	Migut.O00389.1	MgYTHDF1C	TR------------YFIIKSLNHEN--IQL--SVKKEIWATQVMNEPILEEA---FQ---NSGK--VILIFS--VNMSGFFQ-------------------------------------GYAQM---MSSVGWRRD--TIWS-QA-----------------------SGKSN-------PW-GRSFKVKWLRLNDLAFQK-TLHLKNPWNDFK-PVKISRDCQELP-------------------------GDIGRALCELL
Oryza sativa	Os01g22630	OsYTHDF3C	AK------------FFVIKSIGEDD--VHK--SIKYGVWSSSSSGNSKLDIA---FK---DANRI-AKR---------------------------------------NSTKCP-----VFLFFS--VNGSGLFC-------------------------------------GMAEM---VGPVDFHKDM-DFWC-QD-----------------------------------KW-TGSFPVRWHIVKDVPNYT-LQHILLQNNENK-PVTHSRDTQEIP-------------------------YVPGISMLKIL
	Os01g48790	OsYTHDF1C	AL------------FFVIKSYSEDD--IHK--SIKYNVWASTPNGNKRLDNA---FK---LAQER-VAE--KGTKCP-----MFLFFS--VNASGQFC-------------------------------------GVAEM---VGPVDFNRNM-NFWQ-QD-----------------------------------KW-NGFFPVKWHIIKDVPNPQ-FRHIILENNENK-PVTNSRDTQEVK-------------------------FPQGSEMLNIF

	Os03g06240	OsYTH DF1A	AK------------FFVIKSYSEDD--IHK--SIKYNVWASTTNGNKKLDAA---YQ--EAQAK-S----------------------------- -----------------SKCP-----IFLFFS--VNTSGQFV---------------------------------------GVAEM---TGAVDFEKTL-EYWQ-QD-------------------------------------KW-NGSLSLKWHIVKDVPNNI-LKHIILENNENK-PVTNSRDTQEVN--------------------------LDQGIQMLKIF
	Os03g20180	OsYTH DF2C	AK------------FFVIKSYSEDD--VHK--SIKYNVWSSTPNGNKRLDAA---YS--DVQGR-ALG--------------------------- -----------------K---CP-----IFLFFS--VNASGQFC---------------------------------------GVAEM---VGPVDFHKDM-DFWQ-QD-------------------------------------KW-SGSFPVKWHLVKDVPNST-FRHIILENNENK-PVTNSRDTQEIP--------------------------FKSGTNMLKLF
	Os03g53670	OsYTH DF2A	AK------------FFVIKSYSEDD--VHK--SIKYNVWASTPNGNKKLDAG---YR--EAQEK-S----------------------------- -----------------SECP-----VFLFFS--VNTSGQFV---------------------------------------GVAEM---VGPVDFEKTV-DYWQ-QD-------------------------------------KW-NGCFPIKWHVVKDVPNNI-LKHITLDNNDNK-PVTNSRDTQEVK--------------------------LEQGLEMLKIF
	Os04g04000	OsYTH DF5C	AK------------FFMIKSYSEDD--IHK--GIKYNVWASTPHGNNKLDAA---FR--EAQIL-IKE--------------------------- ---------------KGKKCP-----VFLFFS--VNSSGQFV---------------------------------------GLAEI---LGPVDFKKTM-DFWK-LD-------------------------------------RW-NGFFPVTWHIIKDIPNRL-FKHITLENNDNR-IVTFSRDTQEIG--------------------------LLQGLKMLKIF
	Os04g51940	OsYTH DF3B	AK------------FFVIKSYTEDH--VHK--SIKYGVWASTASGNRKLDAA---YR--EAKEK------------------------------- ----------------EATCP-----IFLFFS--VNGSGQFC---------------------------------------GVAEM---IGPVDFDKSV-DYWQ-QD-------------------------------------KW-SGQFPVKWHIIKDVPNSL-LRHIILENNENK-PVTNSRDTQEVR--------------------------LDHGLQMLTIF
	Os05g01520	OsYTH DF4C	AK------------FFMIKSYSEDD--VHK--GIKYNVWASTPNGNNKLDAA---FH--EAQIL-MKE------------------------- ------------------QGKRCP-----IFLFFS--VNTSGQFV---------------------------------------GLAEM---LGPVDFKKTM-DFWQ-QD-------------------------------------KW-NGFFPVMWHIIKDIPNRF-FKHITLENNEGK-VVTFSRDTQEIG--------------------------LPQGLEMLKIF
	Os06g46400	OsYTH DC1A	SR------------YFIVKSCNREN--LEI--SVQQGIWATQRSNEAKLNEA---FE--SIEN-- --------------------VILIFS--INRTRNFQ---------------------------------------GCAKM---TSRIGGYIGG-GNWK-SA------------------------HGTAH--------Y-GRNFSIQWLKLCELSFQK-THHLRNPYNDNL-PVKISRDCQELE--------------------------PFIGEQLASLL
	Os07g07490	OsYTH DF3A	AK------------FFIIKSYSEDD--VHK--SIKYNVWASTSNGNKKLDAA---YQ--EAKEK-S----------------------------- ----------------SDSS-----VFLLFS--VNASGQFV---------------------------------------GLAEM---VGRVDFNKTL-EHWQ-QD-------------------------------------KW-TGCFPVKWHIVKDVPNSL-LKHIILENNENK-PVTNCRDTHEVK--------------------------LEPGLQVLKIF
	Os08g12760	OsYTH DF2B	AK------------FFVIKSYTEDH--VHR--SIKYNVWASTASGNRKLDSA---YR--LAKEK------------------------------- ---------------EDYCP-----IFLFFS--VNGSGQFC---------------------------------------GVAEM---IGPVDFDKSV-DYWQ-QD-------------------------------------KW-SGQFPVKWHIIKDVPNNL-LRHIILENNDNK-PVTNSRDTQEVK--------------------------LEHGLQMLTIF
	Os08g44200	OsYTH DF1B	AR------------FFIIKSYSEDN--VHK--SIKYGVWASTTNGNKKLDSA---YR--EAKEK-------------------------------- --------------EEHCP-----IFLLFS--VNASAQFC---------------------------------------GVAEM---IGPVDFEKSV-DYWQ-QD-------------------------------------KW-TGQFPVKWHIVKDVPNNL-FRHIILENNDNK-PVTNSRDTQEVK--------------------------LEQGMEMLKIF
Panicum hallii	Pahal.B00424	PhYTH DF2A	AK------------FFVIKSYSEDD--VHK--SIKYNVWASTPNGNKKLDAG---YR--EAQEK-S----------------------------- -----------------SDCP-----VFLFFS--VNTSGQFV---------------------------------------GVAEM---VGPVDFDKTV-EYWQ-QD-------------------------------------KW-NGCFPLKWHIVKDVPNNI-LKHITLDNNDNK-PVTNSRDTQEVK--------------------------LEQGLEMLKIF

	Pahal.C00997	PhYTH DF4C	AK------------FFIIKSYSEDD--VHK--SIKYNVWASTPSGNKKLDAA---YL--EAKEK-S--SSSP-----VFLLFS--VNTSGQFV-------------------------------------GLAEM---VGRVDFSKTV-EYWQ-QD-----------------------------------KW-TGCFAVKWHIVKDIPNSL-LKHIILEYNENK-PVTNSRDTQEVK-------------------------LDQGLQMLKIF
	Pahal.C01447	PhYTH DF3C	AK------------FFVIKSYSEDD--IHK--SIKYNVWASTTNGNKKLDAA---YQ---EAQSK-G---SACP-----IFLFFS--VNTSGQFV-------------------------------------GVAEM---TGAVDFEKTL-EYWQ-QD-----------------------------------KW-NGSFSVKWHIVKDVPNNM-LKHIILENNENK-PVTNSRDTQEIH-------------------------LEQGLQMLKIF
	Pahal.D00506	PhYTH DC1A	AK------------FFVIKSIGEAD--VHK--SIKYGVWSSSSNGNSKLDSA---FR---DADRI-SRR--NSTKCP-----VFLFFS--VNGSGHFC-------------------------------------GMAEM---VGPVDFHKDM-DFWC-QD-----------------------------------KW-TGCFPVRWHIVKDIPNYF-LQHITLQNNENK-PVTHSRDTQEIP-------------------------YIPGMSVLKIF
	Pahal.E02120	PhYTH DF1C	AK------------FFVIKSYTEDH--VHK--SIKYNVWASTASGNRKLNTA---YR--EAKDK--EEYCP-----IFLFFS--VNGSGQFC-------------------------------------GVAEM---IGPVDFDKNV-DYWQ-ND-----------------------------------RW-SGQFPVKWHIVKDVPNNI-VRHIILENNEDK-RVTNSRDTQEVK-------------------------LELGVQMLAIF
	Pahal.F00398	PhYTH DF1B	AK------------FFVIKSYTEDH--VHR--SIKYSVWASTASGNRKLDSA---YR---AAKEK---EEHCP-----IFLFFS--VNGSGQFC-------------------------------------GVAEM---IGPVDFDRSV-DYWQ-QD-----------------------------------KW-SGQFPVKWHIIKDVPNNL-LRHIILENNDNK-PVTNSRDTQEVK-------------------------LEQGLQMLTIF
	Pahal.F01360	PhYTH DF2B	AR------------FFIIKSYSEDN--VHK--SVKYGVWASTTNGNKKLDSA---YR---EAKEK--EEHCP-----IFLLFS--VNASAQFC-------------------------------------GVAEM---IGPVDFEKSV-DYWQ-QD-----------------------------------KW-TGQFPVKWHIVKDVPNNL-FRHIILENNDNK-PVTNSRDTQEVK-------------------------LEQGLEMLKIF
	Pahal.G02347	PhYTH DF3B	AL------------FFVIKSYSEDD--IHK--SIKYNVWASTPNGNKRLDNA---YR---LAQER-MAE---KGTKCP-----VFLFFS--VNASGQFC-------------------------------------GVAEM---VGPVDFNRNM-NFWQ-QD-----------------------------------KW-NGFFSVKWHIIKDVPNPQ-FRHIILENNENK-PVTNSRDTQEVK-------------------------FLQGTEMLNIF
	Pahal.H01140	PhYTH DF2C	SR------------YFIVKSCNREN--LEI--SVQQGIWATQRSNEAKLNEA---FE---STEN---VILIFS--INRTRHFQ-------------------------------------GCAKM---TSRIGGYIGG-GNWK-SA-----------------------HGTAH--------Y-GRNFSMQWLKLCELSFQK-THHLRNPYNDNL-PVKISRDCQELE-------------------------PFIGEQLASLL
	Pahal.I00478	PhYTH DF1A	AK------------FFVIKSYDESD--VHK--SIKYGVWSTSSAGNQKLDTA---FR---EAQAI-ASS---TSTLCP-----VLLFFS--VNGSSNFC-------------------------------------GVAEM---VGPVDYQNDM-DFWC-KD-----------------------------------KW-TGSFPVKWHIIKNVRNYT-FRSILLQNNEYK-PVTSSRDTQEIH-------------------------YTPGTTMLELF
	Pahal.I01354	PhYTH DF4A	AK------------FFMIKSYSEDD--IHK--GIKYNVWASTANGNDKLDAA---YH---EAQIL-MKE--NGENCP-----VFLFFS--VNTSGQFV-------------------------------------GLAEM---LGPVDFKKTM-DFWK-ED-----------------------------------KW-NGFFPIIWHIIKDIPNRL-FRHIILEHNDNR-PVTFSRDTQEIG-------------------------LAQGMQMLKIF
	Pahal.I01743	PhYTH DF3A	AK------------FFIIKSYSEDD--VHK--SIKYNVWASTPNGNKKLDAA---YQ---EAKEK-S---SESP-----VFLLFS--VNASGQFV-------------------------------------GLAEM---VGRVDFNKTV-EHWQ-QD-----------------------------------KW-TGCFPVKWHIVKDVPNSL-LKHIILENNENK-PVTNSRDTHEVK-------------------------LEQGLQVLKIF

	Potri.001G002000.1	PtYTH DF2A	AK------------FFVIKSFSEDD--VHK--SIKYSVWTSTPNGNKKLDAA---YK--QGKEN-P--GDCP-----VFLLFS---VNTSGQFV-------------------------------------GLAEM---VGPVDFNKTV-EYWQ-QD-----------------------------------KW-TGCFPLKWHIIKDVPNGC-LRHITLENNENK-PVTNSRDTQEVI-------------------------FEKGVQILKIF
	Potri.001G056100.1	PtYTH DF5B	AK------------FFIIKSFSEDN--VHK--SIKYSIWASTPHGNKKIDAA---YR--EAKEK---EGNCP-----VFLLFS---VNASGQFC-------------------------------------GVAEM---VGPVDFEKDA-DYWQ-QD-----------------------------------RW-NGQFPVQWHIIKDVPNSR-FRHILLENNDHK-PVTHSRDSQEVK-------------------------LEQGIEMLKIF
	Potri.001G113500.1	PtYTH DC1B	AK------------FFIIKSYSEDN--VHK--SIKYGVWASTPNGNRKLDTA---YH--EAKDK---QDPCP-----VFLLFS---VNASAQFC-------------------------------------GVAEM---VGPVDFDKSV-DYWQ-QD-----------------------------------KW-SGQFPVKWHIIKDVPNSQ-FRHIVLENNDNK-PVTNSRDTQEVK-------------------------LEQGVEMLNIF
	Potri.001G357800.1	PtYTH DC1Aa	KL------------YFIVKSCNLEN--LEL--SVQQGVWATQRSNEPKLNEA---FD--SAEN--VILIFS---VNRTRHFQ-------------------------------------GCAKM---TSKIGASVGG-GNWK-YA-----------------------HGTAH--------Y-GRNFSVKWLKLCELSFHK-TRHLRNPFNENL-PVKISRDCQELE-------------------------PSIGEQLASLL
	Potri.003G008400.1	PtYTH DF6C	AF------------FFVIKSYSEDD--IHK--SIKYNVWASTPNGNKRLNSA---YL--DSQQK-IAQ--IGCSCP-----VFLFFS---VNASGQFC-------------------------------------GVAEM---TGRVDFNKNM-DFWQ-QE-----------------------------------KW-NGYFPVKWHIIKDIPNPQ-LRHIILENNENK-PVTNSRDTQEVK-------------------------FPQGIEILNIF
Populus trichocarpa	Potri.003G222700.1	PtYTH DF1A	AK------------FFVIKSYSEDD--VHK--SIKYNVWSSTPHGNKKLQTA---FE--DAQKL-AVG--RPRGCP-----IFLFFS---VNASGQFC-------------------------------------GVAEM---IGPVDLHRDM-DFWQ-QD-----------------------------------KW-SGSFLVKWHIIKDIPNSS-FRHIILENNENK-PVTNSRDTQEIM-------------------------YKQGLEMLKTF
	Potri.004G223800.1	PtYTH DF5C	AK------------FFVIKSYSEDD--VHK--SIKYNVWSSTPHGNKKLHSA---FE--YAQKL-DLG--RPRGCP-----IFLFFS---VNASGQFC-------------------------------------GVAEM---VGPVDFNRDM-DFWQ-QD-----------------------------------KW-SGSFLVKWHIIKDIPNSS-FRHIILENNENK-PVTNSRDTQEIM-------------------------YKQGLEMLKMF
	Potri.006G079900.1	PtYTH DF3B	AF------------FFVIKSYSEDD--IHK--SIKYNVWASTPNGNKRLDSA---YQ--DAQQK-IAE--KGNSCP-----VFLFFS---VNASGQFC-------------------------------------GVAEM---VGRVDFNKNM-DFWQ-QD-----------------------------------KW-NGYFPVKWHIIKDVPNPQ-LRHIILENNENK-PVINSRDTQEVK-------------------------FPQGIEILNIF
	Potri.007G002800.1	PtYTH DF2B	AK------------FFIIKSYSEDN--VHK--SIKYGVWASTPNGNRKLDTT---YR--EAKEK---QDPCP-----VFLLFS---VNASAQFC-------------------------------------GVAEM---TGPVDFDKSV-DYWQ-QD-----------------------------------KW-SGQFPVKWHIIKDVPNSQ-FRHIVLENNDNK-PVTNSRDTQEVK-------------------------LEQGIEMLNIF
	Potri.008G080800.1	PtYTH DF4C	AK------------FFIIKSFSEDN--VHK--SIKYSVWASTPHGNKKIDAA---YR--EAKEK---EGNCP-----VFLLFS---VNASGQFC-------------------------------------GVAEM---VGPVDFEKDA-EYWQ-QD-----------------------------------RW-NGQFPVQWHIVKDVPNSR-FRHILLENNDNK-PATHSRDSQEVK-------------------------LEQGIEMLKIF
	Potri.008G100200.1	PtYTH DF2C	AK------------FFVIKSYNEDD--IHK--SIKYDVWASTPNGNKKLDAA---FH--NAEEV-SSE--TGTKCP-----IFLFFS---VNGSGQFV-------------------------------------GLAEM---VGQVDFNKDM-DFWQ-ID-----------------------------------KW-NGFFPVKWHVIKDIPNGQ-LRHIVLENNDGH-SVTFSRDTQEIG-------------------------LEKGLEMLNIF

	Potri.010G152300.1	PtYTHDF1C	AK------------FFIIKSYSEDD--VHK--CIKYNVWASTPNGNKKLDAA---YQ--EAEQK-S---GGCP-----VFLLFS--VNTSGQFV-------------------------------------GLAEM---TGRVDFDKSV-EYWQ-QD-----------------------------------KW-TGYFPVKWHIVKDVPNSF-LKHITLENNENK-PVTNSRDTQEVK-------------------------LEQGLKLIKIF
	Potri.010G175500.1	PtYTHDF3C	AK------------FFVIKSYSEDD--IHK--SIKYDVWASTPNGNKKLDAA---FH--NAEEV-SSD--TGYKCP-----IFLFFS--VNGSGQFV-------------------------------------GFAEM---VGQVDFNKDM-DFWQ-ID-----------------------------------KW-NGFFPVKWHVVKDIPNGH-LRHIVLENNDGH-SVTFSRDTQEIV-------------------------LKQGLEMLNIF
	Potri.011G089800.1	PtYTHDC1Ab	SQF----------VYFIVKSCNREN--LEL--SVQQGVWATQRSNEIKLNEA---LD--SADN--VILIFS--VNRTRHFQ-------------------------------------GCAKM---ASKIGASVGG-GNWK-YA-----------------------HGTAH--------Y-GRNFSVKWLKLCELSFHK-TRHLRNPFNENL-PVKISRDCQELE-------------------------PSIGEQLASLL
	Potri.014G001000.1	PtYTHDF1B	TR------------YFIIKSLNQHN--IQL--SIENGIWATQVRNEPILEEA---FH--NSGR--VILIYS--VNMSGFFQ-------------------------------------GYAQM---ISSVGWRHD--NLWS-EG-----------------------SGKSN-------PW-GRSFKVKWLRLNDLPFQK-TLHLKNPLNDYK-PVKISRDCQELP-------------------------EDIGEALCELI
	Potri.018G149800.1	PtYTHDF4B	AK------------FFVIKSYSEDN--VHK--SIKYGVWASTPNGNKKLDAA---YR--EAKEN---HGTCP-----IFLLFS--VNASAQFC-------------------------------------GVAEM---VGPVDFDKNV-DFWQ-QD-----------------------------------KW-SGQFPVKWHIIKDVPNSQ-FRHIVLENNDNK-PVTNSRDTQEVE-------------------------LEQGAEMLGIF
	Potri.019G034300.1	PtYTHDF3A	AK------------FFIIKSYSEDD--VHK--CIKYNVWASTPNGNKKLDAA---YQ--EAGQK-S--GGCP-----VFLLFS--VNTSGQFV-------------------------------------GLAEM---TGRVDFDKSV-EYWQ-QD-----------------------------------KW-TGYFPVKWHFVKDVPNSL-LKHITLENNENK-PVTNSRDTQEVK-------------------------LEQGLEMIKIF
Physcomitrella patens	Pp3c10_17810V3.1	PpaYTHDF2	TR------------YFIIKSLNHHN--IEK--SIEKGIWATQAMNETVLNEA---FE--TSEK--VVLVFS--VNMSSHFQ-------------------------------------GYALM---SSPIGQRRA--NIWS-EA-----------------------NEGAN-------PW-GGTFHVEWLRLYDLPFQK-TVHLKNPLNAFK-PVKISRDCQELT-------------------------QAIGKALCALI
	Pp3c20_9290V3.1	PpaYTHDC1A	AK------------FFVIKSYSEDD--VHK--SIKYNVWASTPVGNKRLDAA---YQ--EALTK-CNG--DTKSYP-----VFLFFS--VNASGQFC-------------------------------------GMAQM---TGRVDFSKSV-DYWQ-QD-----------------------------------KW-NGRFPVTWHIIKDIPNCQ-FRHIILENNDNK-PVTNSRDTQEVK-------------------------FEQGMAMLNIF
	Pp3c3_33560V3.1	PpaYTHDF1	CR------------YFIVKSSNREN--LEL--SVERGLWATHRNNEAKLNDA---FD--SCEH--VIFIFS--VNETRHFQ-------------------------------------GCARM---MSKIGGVAGG-GAWK-YA-----------------------HGTAN--------Y-GRNFRLKWLKLCELSFYK-TRHLRNSYNENM-PVKISRDCQELE-------------------------PSVGEQLALLL
	Pp3c8_11880V3.1	PpaYTHDC1B	AK------------FFVIKSYSEDD--VHK--SIKYNVWASTPVGNKRLDAA---YL--EALAK-SNG--DTKSFP-----VFLFFS--VNASGQFC-------------------------------------GVAQM---TGPVDFSKSV-DFWQ-QD-----------------------------------KW-NGRFPVVWHIIKDIPNCQ-FRHIILENNDNK-PVTNSRDTQEVK-------------------------FEQGFGMLNIF
Saccharomyces cerevisiae	Sacce1/1410	PHO92 (ScYTHDF1)	SR------------FFVIKSSSLKH--VKR--SFYNGIWSSTHFGNKRLSEA---YK--KLNSG---AK-----VFLFFS--INTSGRFC-------------------------------------GVAEM---VSDLKMDLDT-SIWEDEQ-----------------------------------KY-GKAFKVRWVIVRDINNRS-LKRFLIPSNEMK-PITHSRDTQEIP-------------------------YSIGISIINLF

Schizosaccharomyces pombe	Schpo1/4324	MMI1 (SpYTHDC1)	SR------------YFIMLCDNETA--IAH--AKKTSIWAVKKDSSKRISDA---YK--KAS---VYFIFV--AQQTYNAL--------------------------------------GYAQV---VSDLNSTELP--FWSDSS---------------------------------H------AGGVRIKWIKTCNLFSAE-ISEIVSHMDHGS----EARDGMEMM--------------------------YDEGSRLCTLI
Setaria italica	Seita.2G046200.1	SiYTHDF3A	AK------------FFIIKSYSEDD--VHK--SIKYNVWASTPNGNKKLDAA---YQ---EAKEK-S--SESP-----VFLLFS--VNASGQFV--------------------------------------GLAEM---VGRVDFNKTV-EHWQ-QD------------------------------------KW-TGCFPVKWHIVKDVPNSL-LKHIILENNENK-PVTNSRDTHEVK--------------------------LEPGLQVLKIF
	Seita.2G149700	SiYTHDF5C	AK------------FFVIKSYNEAD--IHK--SIKYGVWSTSSVGNLKLDTA---FR---DAQVI-AAS--SSTLCP-----VLLFFS--VNGSSHFC--------------------------------------GVAEM---VGPVDYQNDM-DFWCRKN------------------------------------KW-IGSFPVKWHIIKNVHNST-FRSISLQNNEDK-PVTSSRDTQEIH--------------------------YTPGTTMLELF
	Seita.3G070000.1	SiYTHDF3C	AK------------FFMIKSYSEDD--IHK--GIKYNVWASTPNGNNKLDAA---YH---DAQIL-MKE--NGEKCP-----VFLFFS--VNTSGQFV--------------------------------------GLAEM---LGPVDFKKTM-DFWK-DD------------------------------------RW-SGFFPIIWHIIKDIPNRL-FRHIILEYNDNR-PVTFSRDTQEIG--------------------------LLQGVQMLKIF
	Seita.4G262400.1	SiYTHDC1A	SR------------YFIVKSCNREN--LEI--SVQQGIWATQRSNEAKLNEA---FE---STEN---VILIFS--INRTRHFQ---------------------------------------GCAKM---TSRIGGYIGG-GNWK-SA------------------------HGTAH--------Y-GRNFSMQWLKLCELSFQK-THHLRNPYNDNL-PVKISRDCQELE--------------------------PFIGEQLASLL
	Seita.5G156800.1	SiYTHDF2C	AK------------FFVIKSIGEAD--VHK--SIKYGVWSSSSNGNSKLDSA---FR---DADRI-SRR--NSTKCP-----VFLFFS--VNGSGHFC--------------------------------------GMAEM---VGPVDFHKDM-DFWC-QD------------------------------------KW-TGCFPVRWHIVKDIPNYS-LQHITLQNNENK-PVTHSRDTQEIP--------------------------YIPGMSVLKIF
	Seita.5G271100.1	SiYTHDF1C	AL------------FFVIKSYSEDD--IHK--SIKYSVWASTPNGNKRLDNA---YR---LAQER-MAE--KGTKCP-----VFLFFS--VNASGQFC--------------------------------------GVAEM---VGPVDFNRNM-NFWQ-QD------------------------------------KW-NGFFSVKWHIIKDVPNPQ-FRHIILENNENK-PVTNSRDTQEVK--------------------------FPQGTEMLNIF
	Seita.6G022500.1	SiYTHDF1B	AK------------FFVIKSYTEDH--VHR--SIKYSVWASTASGNRKLDSA---YR---AAKEK--EDHCP-----IFLFFS--VNGSGQFC--------------------------------------GVAEM---IGPVDFDRSV-DYWQ-QD------------------------------------KW-SGQFPVKWHIIKDVPNNL-LRHIILENNDNK-PVTNSRDTQEVK--------------------------LEQGLQMLTIF
	Seita.6G246600.1	SiYTHDF2B	AR------------FFIIKSYSEDN--VHK--SVKYGVWASTTNGNKKLDSA---YR---EAKEK--EEQCP-----IFLLFS--VNASAQFC--------------------------------------GVAEM---IGPVDFEKSV-DYWQ-QD------------------------------------KW-TGQFPVKWHIVKDVPNNL-FRHIILENNDNK-PVTNSRDTQEVK--------------------------LEQGLEMLKIF
	Seita.7G231300.1	SiYTHDF3B	AK------------FFVIKSYTEDH--IHK--SIKYNVWASTASGNRKLNAA---YR---EAKER---EDYCP-----IFLFFS--VNGSGQFC--------------------------------------GVAEM---IGPVDFDKSV-DYWQ-ND------------------------------------RW-SGQFPVKWHTVKDVPNNL-VRHIILENNENK-RVTNSRDTQEVK--------------------------LEQGVQMLAIF
	Seita.7G270400.1	SiYTHDF4C	AK------------FFVIKSYNEAD--IHK--SIKYGVWSTSSVGNLKLDTA---FR---DAQVI-AAS--SSTLCP-----VLLFFS--VNGSSHFC--------------------------------------GVAEM---VGPVDYQNDM-DFWCRKN------------------------------------KW-IGSFPVKWHIIKNVHNST-FRSILLQNNEDK-PVTSSRDTQEIH--------------------------YTPGTTMLELF

	Seita.9G059600.1	SiYTH DF4A	AK------------FFIIKSYSEDD--VHK--SIKYNVWASTPSGNKKLDAA---YV--EAKEK-S--SSSP-----VFLLFS--VNTSGQFV-------------------------------------GLAEM---VGRVDFNKTV-EYWQ-QD-----------------------------------KW-TGCFPVKWHIVKDIPNGL-LKHIILEYNENK-PVTNSRDTQEVK-------------------------LDQGLQVLKIF
	Seita.9G092900.1	SiYTH DF2A	AK------------FFVIKSYSEDD--VHK--SIKYNVWASTPNGNKKLDAG---YR--EAQEK-S--SDCP-----VFLFFS--VNTSGQFV-------------------------------------GVAEM---VGPVDFDKTV-EYWQ-QD-----------------------------------KW-NGCFPLKWHIVKDVPNNI-LKHITLDNNDNK-PVTNSRDTQEVK-------------------------LEQGLEMLKIF
	Seita.9G534000.1	SiYTH DF1A	AK------------FFVIKSYSEDD--IHK--SIKYNVWASTTNGNKKLDAA---YQ--EAQSK-G--SACP-----IFLFFS--VNTSGQFV-------------------------------------GVAEM---TGAVDFEKTL-EYWQ-QD-----------------------------------KW-NGSFSLKWHIVKDVPNNM-LKHIILENNENK-PVTNSRDTQEIH-------------------------LEQGLQMLKIF
Solanum lycopersicum	Solyc01g028860.2.1	SlYTH DF1A	AK------------FFIIKSYSEDD--VHK--SIKYNVWASTPNGNKKLDAA---YQ--EAQQN-S--GGCP-----VFLFFS--VNTSGQFV-------------------------------------GVAEM---VGPVDFNKNV-EYWQ-QD-----------------------------------KW-VGCFPVKWHIVKDVPNSL-LKHITLENNENK-PVTNSRDTQEVK-------------------------VEQGLQVLKIF
	Solyc01g103540.2.1	SlYTH DF2C	AK------------FYIIKSYSEDD--IHK--CVKYDVWSSTPNGNKKLDTA---FV--ESEAK-ASG--TGSSCP-----VFLFFS--VNGSGQFL-------------------------------------GVAEM---VGQVDFNRNM-DFWQ-LD-----------------------------------KW-SGFFPLKWHIVKDVPNTQ-FRHIILENNDNR-PVTYSRDTQEIG-------------------------LKEGLEMLNIL
	Solyc02g021760.2.1	SlYTH DC1A	SR------------YFIVKSCNREN--LEL--SVQQGVWATQRSNEAKLNEA---FD--SVEN--VILIFS--VNRTRHFQ-------------------------------------GCGKM---TSRIGGAANG-GNWK-HE-----------------------HGTAH--------Y-GRNFSLKWLKLCELSFQK-THHLRNPYNENL-PVKISRDCQELE-------------------------PSVGEQLASLL
	Solyc02g070240.2.1	SlYTH DC1Ab	SR------------YFIVKSCNREN--LEL--SVQQGVWATQRSNEAKLNEA---FD--SVEN--VILIFS--INRTRHFQ-------------------------------------GLAKM---TSRIGGAAKG-GNWK-HE-----------------------HGTAH--------Y-GRNFSVKWLKLCELSFQK-TRHLRNPYNENL-PVKISRDCQELE-------------------------ISVGEQLASLL
	Solyc05g032850.2.1	SlYTH DF1B	AK------------FFVIKSFSEDN--VHK--SIKYGVWASTPQGNRKLDAA---YA--EAKEM---NANCP-----VFLFFS--VNASGQFC-------------------------------------GVAEM---VGPIDFENSA-EYWQ-QD-----------------------------------RW-SGKFPVKWHVIKDVPNSQ-FRHILLEHNDNK-PVTHSRDSQEVK-------------------------LPEGLEMLKIF
	Solyc08g007730.2.1	SlYTH DC1B	IR------------YFVIKSLNHEN--IQL--SVNRGIWATQAMNEAILDEA---FH--NSSK--VILIFS--VNTSGYFQ-------------------------------------GYAQM---ISSV-GLRRD-QVWS-QG-----------------------NGGRN-------SW-GRSFEVNWLRLYDLPFQR-TLHLKNPWNQNK-PVKISRDCQELP-------------------------PDIGEALCELL
	Solyc12g099090.1.1	SlYTH DF1C	AK------------FFVIKSYSEDD--VHK--SIKYNVWSSTPNGNKKLNSA---YE--DAQRI-TPG--NPRGCP-----IFLFFS--VNASGQFC-------------------------------------GVAEM---TGPVDFYKDM-DFWQ-QD-----------------------------------KW-SGSFPVKWHFIKDVPNPN-FRHIILENNENK-PVTNSRDTQEIR-------------------------YKKGIEMLKVF
Sphagnum fallax	Sphfalx0029s0025.1	SfYTH DF1	AK------------FFVIKSYSEDD--VHK--SIKYNVWASTPVGNKRLDAA---FK--DASGK--AVGGCP-----VFLFFS--VNASGQFC-------------------------------------GVAEM---TGPVDFTQSV-EYWQ-QD-----------------------------------KW-NGRFTVKWHIIKDIPNCQ-FRHIILENNDNK-PVTNSRDTQEVR-------------------------FEQGIEMLSIF

	Sphfalx0037s0069.1	SfYTH DF2	AK------------FFVIKSYSEDD--VHK--SIKYNVWASTPAGNKRLDAA---FL--ESQSK-AGG-----------------------<br>-----------------KQGSCP-----VFLFFS--VNASGQFC-------------------------------------GVAEM---<br>VGPVDFGKSV-EYWQ-QD-----------------------------------KW-NGRFTVKWHIIKDIPNCQ-FRHIILENNDNK-PVTNSRDTQEVK-------------------------FEQAISMLNIF
	Sphfalx0040s0172.1	SfYTH DF3	AK------------FFVIKSYSEDD--VHK--SIKYNVWASTPAGNKRLDAA---FL--EAQNKVASG----------------------<br>------------------KPGSCP-----VFLFFS--VNASGQFC-------------------------------------GVAEM---<br>VGPVDFGKSV-EYWQ-QD-----------------------------------KW-NGCFAVKWHIIKDIPNCH-FRHIILENNDNK-PVTNSRDTQEVK-------------------------FEQAIGMLNIF
	Sphfalx0156s0007.1	SfYTH DC1B	TK------------YFIIKSLNHHN--LAK--SIEKGIWATQAMNEPVLNEA---FE---TCER-----------------------------------<br>---------------------VVLVFS--VNMSSHFQ-------------------------------------GYARM---ASPIGRRRA--<br>NIWS-EA-----------------------NEGAN-------PW-GGTFRVEWLCLYDLPFQK-TVHLKNPLNSFK-PVKISRDCQELT-------------------------KEIGEALCALI
	Sphfalx0235s0015.1	SfYTH DC1A	CR------------YFIVKSNNREN--LEL--SVERGLWATHRNNEAKLNEA---FD--TCDH--------------------------------<br>------------------------VILVFS--VNETRHFQ-------------------------------------GCARM---MSKIGGVIGG-<br>GGWK-YA-----------------------HGTAH--------Y-GRNFNLRWLKLCELSFNK-TRHLRNAYNENL-PVKISRDCQELE-------------------------PSVGEQLASLL
Zostera marina	Zosma103g00140.1	ZmaYT HDF3C	AR------------FFVIKSYSEDN--VHK--SIKYGLWASTPNGNRKLDAA---YR--EAKEK------------------------------<br>---------------GGACP-----VFLFFS--VNSSAQFC-------------------------------------GVAEM---<br>IGHVDFNKSV-DYWQ-QD-----------------------------------KW-SGNFPLKWHIVKDVPNSM-FRHIILENNDKK-PVTNSRDTQEIK-------------------------<br>LKQGLEMLSIF
	Zosma105g00190.1	ZmaYT HDF2C	AR------------FFVIKSYSEDD--VHK--SIKYNMWSSTPSGNKRLDVA---YD--GAKKI-SES------------------------<br>----------------KKMLCP-----VFLFFS--VNASGQFC-------------------------------------GVSEM---<br>KGPVDFHKDM-DFWQ-QD-----------------------------------KW-SGSFPVKWHIVKDVANSN-FRHIILENNENK-SVTNSRDTQEVP-------------------------<br>YKAGVSMLNIF
	Zosma105g00770.1	ZmaYT HDF5C	AK------------FYIIKSYSEDD--IHK--SIKYNVWASTPNGNKKLDAG---YQ--EAQEK-S----------------------------<br>----------------GGLP-----LFLFFS--VNTSGQFV-------------------------------------GVAEM---<br>VGNVDFNKTV-DYWQ-QD-----------------------------------KW-NGCFPVKWHIVKDVPNSI-LKHITLENNDNK-PVTNSRDTQEVK-------------------------<br>FEQGIQLLKLF
	Zosma14g00600.1	ZmaYT HDF4C	SR------------YFIVKSCNREN--LEL--SVQQGAWATQRSNEAKLNGA---FE--TTDN--------------------------------<br>------------------------VILLFS--INRTRHFQ-------------------------------------GCAKM---TSKIGGYVGG-<br>GNWK-YA-----------------------HGTPH--------Y-GRNFSIKWLKLGELSFHK-TRHLRNPYNDNL-PVKISRDCQELD-------------------------PFVGPQLATLL
	Zosma158g00160.1	ZmaYT HDC1A	AL------------FFIIKSYSEDD--IHK--SIKYNVWTSTSNGNKKLDNA---FH--KIQEN-IGERG-----------------------<br>--------------LSSSKCP-----VFLFFS--VNASGQFC-------------------------------------GVAEM---<br>IGGVDFNKHW-NFWQ-QD-----------------------------------KW-NGSFPVKWHIIKDVPNSH-FRHIILANNDSK-PVTNSRDTQEIM-------------------------FQQGSDMLSIF
	Zosma39g00650.1	ZmaYT HDF1A	AK------------FFMIKSYNEDD--VHK--SIKYNVWASTSSGNKKLNDA---FR--VVENI-FKE------------------------<br>----------------SSIKCP-----IFLFFS--VNTSGKFL-------------------------------------GIAEM---<br>VGQVDFKKSM-DFWQ-EE-----------------------------------RW-SGFFPLQWHIVKDVPNIN-FKNITLPNNDNR-LVYYSRDTQEIP-------------------------LLQGLEMLKIF
	Zosma467g00180.1	ZmaYT HDF1C	AL------------FFIIKSYSEDD--IHK--SIKYNVWASTPNGNKRLDAA---FQ--SAQNQ-MKE-------------------------<br>---------------KGCACP-----VFLFFS--VNASGQFC-------------------------------------GVAEM---<br>TGRVDFNKNM-DFWQ-QD-----------------------------------KW-NGYIPVKWHIIKDVPNPH-FRHIILENNDNK-PVTNSRDTQEVL-------------------------<br>FLRGSDMLKIF

	Zosma91g00280.1	ZmaYTHDF1B	AS------------FFVIKSYSEDD--IHK--SIKYNVWASTLNGNKILDSA---FQ--TAEEKMMKEE---------------------------------------GNNHHP-----IFLFFS--VNASGQFC-------------------------------------GVAEM---IGRVDFNKNM-GFWQ-QD-----------------------------------KW-NGFFPVKWHIIKDVPNPQ-LRHIKLENNDYK-PVTNSRDTQEVK-------------------------LTQGSEMLNIF
	tr|E1BAQ2|E1BAQ2	BtYTHDF1	GR------------VFIIKSYSEDD--VHR--SIKYSLWCSTEHGNRRLDSA---FR--ALGSK--GP-----VYLLFS--VNGSGHFC-------------------------------------GVAEM---KSPVDYGTSA-GVWS-QD-----------------------------------KW-KGKFDVKWIFVKDVPNSQ-LRHIRLENNDNK-PVTNSRDTQEVP-------------------------LEKARQVLRII
	tr|E1BH80|E1BH80	BtYTHDF3	GR------------VFIIKSYSEDD--IHR--SIKYSIWCSTEHGNKRLDAA---YR--SLNGK--GP-----LYLLFS--VNGSGHFC-------------------------------------GVAEM---KSVVDYNAYA-GVWS-QD-----------------------------------KW-KGKFEVKWIFVKDVPNNQ-LRHIRLENNDNK-PVTNSRDTQEVP-------------------------LEKAKQVLKII
Bos taurus	sp|Q0VCZ3|YTHD2	BtYTHDF2	GR------------VFIIKSYSEDD--IHR--SIKYNIWCSTEHGNKRLDAA---YR--SMNGK--GP-----VYLLFS--VNGSGHFC-------------------------------------GVAEM---KSAVDYNTCA-GVWS-QD-----------------------------------KW-KGRFDVRWIFVKDVPNSQ-LRHIRLENNENK-PVTNSRDTQEVP-------------------------LEKAKQVLKII
	XP_005208101	BtYTHDC1	AR------------FFLIKSNNHEN--VSL--AKAKGVWSTLPVNEKKLNVA---FR--SARS--VILIFS--VRESGKFQ-------------------------------------GFARL---SSESHHGGSP-IHWV-LP----------------------AGMSAK-------ML-GGVFKIDWICRRELPFTK-SAHLTNPWNEHK-PVKIGRDGQEIE-------------------------LECGTQLCLLF
	NP_001179740	BtYTHDC2	IR------------YFIMKSSNLRN--LEI--SQQKGIWSTTPSNERKLNRA---FW--ESSM--VYLVFS--VQGSGHFQ-------------------------------------GFSRM---SSEIGREKSQ-------------------------------DWGSA-------GL-GGVFKVEWIRKESLPFQF-AHHLLNPWNDNK-KVQISRDGQELE-------------------------PQVGEQLLQLW
	tr|A0A1S3GAX5|A0A1S3GAX5	DoYTHDF1	GR------------VFIIKSYSEDD--IHR--SIKYSIWCSTEHGNKRLDSA---FR--AVASK--GP-----VYLLFS--VNGSGHFC-------------------------------------GVAEM---KSAVDYGTSA-GVWS-QD-----------------------------------KW-KGKFDVKWIFVKDVPNSQ-LRHIRLENNDHK-PVTNSRDTQEVP-------------------------LEKAKQVLKII
	tr|A0A1S3EK84|A0A1S3EK84	DoYTHDF3	GR------------VFIIKSYSEDD--IHR--SIKYSIWCSTEHGNKRLDAA---YR--SLNGK--GP-----LYLLFS--VNGSGHFC-------------------------------------GVAEM---KSVVDYNAYA-GVWS-QD-----------------------------------KW-KGKFEVKWIFVKDVPNNQ-LRHIRLENNDNK-PVTNSRDTQEVP-------------------------LEKAKQVLKII
Dipodomys ordii	tr|A0A1S3GQM8|A0A1S3GQM8	DoYTHDF2	GR------------VFIIKSYSEDD--IHR--SIKYNIWCSTEHGNKRLDAA---YR--SMNGK--GP-----VYLLFS--VNGSGHFC-------------------------------------GVAEM---KSAVDYNTCA-GVWS-QD-----------------------------------KW-KGRFDVRWIFVKDVPNSQ-LRHIRLENNENK-PVTNSRDTQEVP-------------------------LEKAKQVLKII
	XP_012864393	DoYTHDC1	AR------------FFLIKSNNHEN--VSL--AKAKGVWSTLPVNEKKLNLA---FR--SARS--VILIFS--VRESGKFQ-------------------------------------GFARL---SSESHHGGSP-IHWV-LP----------------------AGMSAK-------ML-GGVFKIDWICRRELPFTK-SAHLTNPWNEHK-PVKIGRDGQEIE-------------------------LECGTQLCLLF
	XP_012871461	DoYTHDC2	VR------------YFIMKSSNLRN--LEI--SQQKGIWSTTPSNERKLNRA---FW--ESSM--VYLVFS--VQGSGHFQ-------------------------------------GFSRM---SSEIGREKSQ-------------------------------DWGSA-------GL-GGVFKVEWIRKESLPFQC-AHHLLNPWNDNK-KVQISRDGQELE-------------------------PQVGEQLLQLW
Callorhinchus milii	XP_007909300	CmYTHDF1	GR------------VFIIKSYSEDD--IHR--SIKYSIWCSTEHGNKRLDSA---YR--SMAGK--GP-----VYLLFS--VNGSGHFC-------------------------------------GVAEM---KSAVDYGTCA-GVWS-QD-----------------------------------KW-KGKFDVKWIFVKDVPNNQ-LRHIRLENNDNK-PVTNSRDTQEVP-------------------------LEKAKQVLKII

	XP_007894925	CmYTH DF3	GR------------VFIIKSYSEDD--IHR--SIKYSIWCSTEHGNKRLDAA---FR---SLNGK--GP-----LYLLFS---VNGSGHFC-----------------------------------GVAEM---KSAVDYNAYA-GVWS-QD---------------------------------KW-KGKFEVKWIFVKDVPNNQ-LRHIRLENNDNK-PVTNSRDTQEVP-----------------------LEKAKQVLKII
	XP_007893091	CmYTH DF2	GR------------VFIIKSYSEDD--IHR--SIKYSIWCSTEHGNKRLDSA---YR--TMNGK---GP-----VYLLFS---VNGSGHFC-----------------------------------GVAEM---KSTVDYSTCA-GVWS-QD---------------------------------KW-KGRFDVRWIFVKDVPNSQ-LRHIRLENNENK-PVTNSRDTQEVP-----------------------LEKAKQVLKII
	XP_007902407	CmYTH DC1	AR------------FFLIKSNNHEN--VSL--AKAKGVWSTLPVNEKKLNTA---FR---DARS---VILIFS---VRESGKFQ-----------------------------------GFARL---SSESHHGGSP-IHWV-LP--------------------AGMNAK-------ML-GGVFKIDWICRRELPFTK-TSHLVNLWNENK-PVKIGRDGQEIE-----------------------PDCGTQLCLLF
	XP_007895610	CmYTH DC2	VR------------FFILKSSNLRN--LEI--SQQKGIWSTTPSNERKLNRA---FW---ESSI---VYLVFS---VQGSGHFQ-----------------------------------GFARM---ISEIGRERSQ-------------------------------EWGSA-------GL-GGVFRVEWIRKESLPFQY-AHHLLNPWNDNK-KVQISRDGQELE-----------------------PQVGEQLVQLW
Xenopus tropicalis	tr|Q6P1V5|Q6P1V5	XtYTH DF1	GR------------VFIIKSYSEDD--IHR--SIKYSIWCSTEHGNKRLDSA---FR---SMNGK--GP-----VYLLFS---VNGSGHFC-----------------------------------GVAEM---KSPVDYGTSA-GVWS-QD---------------------------------KW-KGKFDVKWLFVKDVPNNQ-LRHIRLENNDNK-PVTNSRDTQEVP-----------------------LEKAKLVLKII
	tr|B1H2Z7|B1H2Z7	XtYTH DF3	GR------------VFIIKSYSEDD--IHR--SIKYTIWCSTEHGNKRLDAA---YR--SLNGK---GP-----LYLLFS---VNGSGHFC-----------------------------------GVAEM---KSVVDYNAYA-GVWS-QD---------------------------------KW-KGKFDVKWVFVKDVPNNQ-LRHIRLENNDNK-PVTNSRDTQEVP-----------------------LEKAKQVLKII
	XP_002934690	XtYTH DF2	GR------------VFIVKSYSEDD--IHR--SIKYNVWCSTEHGNKRLDAA---YR--SLNGK---GP-----LYLLFS--VNGSGHFC-----------------------------------GVAEM---RSAVDYNTCA-GVWS-QD---------------------------------KW-KGRFDVRWLFVKDVPNGQ-LRHIRLENNDNK-PVTNSRDTQEVP-----------------------LEKARQVLRII
	NP_001008121	XtYTH DC1	AR------------FFLIKSNNHEN--VSL--AKAKGVWSTLPVNEKKLNAA---FR---SARS---VILVFS---VRESGKFQ-----------------------------------GFARL---SSESHHGGSP-IHWV-LP--------------------AGMNAK-------ML-GGVFKIDWICRRELPFTK-CVHLTNPWNEHK-PVKIGRDGQEIE-----------------------PDCGTQLCLLF
	XP_004910524	XtYTH DC2	VR------------YFIMKSSNLRN--LEI--SQQKGIWSTTPSNERKLNQA---FW--ESST---VYLVFS--VQGSGHFQ-----------------------------------GFARM---TSEIGREKSQ------------------------------------DWGST-------SL-GGVFKVEWIRKESLPFQQ-AHHLLNPWNDNK-KVQISRDGQELE-----------------------TLVGEQLLLLW
Ornithorhynchus anatinus	tr|F7DG05|F7DG05	OaYTH DF1	GR------------VFIIKSYSEDD--IHR--SIKYSIWCSTEHGNKRLDSA---FR---SMNSK--GP-----VYLLFS---VNGSGHFC-----------------------------------GVAEM---KSPVDYGTSA-GVWS-QD---------------------------------KW-KGKFDVKWIFVKDVPNNQ-LRHIRLENNDNK-PVTNSRDTQEVP-----------------------LEKAKQVLKII
	tr|F6Q844|F6Q844	OaYTH DF3	GR------------VFIIKSYSEDD--IHR--SIKYSIWCSTEHGNKRLDAA---YR--SLNGK--GP-----LYLLFS---VNGSGHFC-----------------------------------GVAEM---KSVVDYNAYA-GVWS-QD---------------------------------KW-KGKFDVKWVFVKDVPNNQ-LRHIRLENNDNK-PVTNSRDTQEVP-----------------------LEKAKQVLKII
	XP_007662869	OaYTH DF2	GR------------VFIIKSYSEDD--IHR--SIKYNIWCSTEHGNKRLDAA---FR--SLNAK--GP-----VYLLFS---VNGSGHFC-----------------------------------GVAEM---CSAVDYNTCA-GVWS-QD---------------------------------KW-KGRFDVRWIFVKDVPNSQ-LRHIRLENNENK-PVTNSRDTQEAP-----------------------LEKAKQVLKII

	XP_028929054	OaYTHDC1	AR------------FFLIKSNNHEN--VSL--AKAKGVWSTLPVNEKKLNAA---FR---SARS--VILIFS---VRESGKFQ------------------------------------GFARL---SSESHHGGSP-IHWV-LP---------------------AGMNAK-------ML-GGVFKIDWICRRELPFTK-SAHLTNPWNEHK-PVKIGRDGQEIE------------------------LECGTQLCLLF
	XP_028912180	OaYTHDC2	VR------------YFIMKSSNSRN--LEI--SQQKGVWSTTPVNEGKLNQA---FF---DSTT--VFLVFS---VQGSGHFQ------------------------------------GFSRM---ISEIGWEKSE------------------------------DWGSS-------GL-GGMFKVDWIRKERLPFQQ-THHLLNPWNENK-KVQISRDGQELE------------------------PQIGEQLLKLW
Gallus gallus	tr|Q5F3J9|Q5F3J9	GgYTHDF1	GR------------VFIIKSYSEDD--IHR--SIKYTIWCSTEHGNKRLDSA---FR---SMNSK--GP-----VYLLFS---VNGSGHFC------------------------------------GVAEM---KSPVDYGTSA-GVWS-QD----------------------------------KW-GKGFDVKWIFVKDVPNNQ-LRHIRLENNDNK-PVTNSRDTQEVH------------------------LEKAKQVLKII
	tr|Q5ZLM1|Q5ZLM1	GgYTHDF3	GR------------VFIIKSYSEDD--IHR--SIKYSIWCSTEHGNKRLDAA---YR---SLNGK--GP-----LYLLFS---VNGSGHFC------------------------------------GVAEM---KSVVDYNAYA-GVWS-QD----------------------------------KW-GKGFDVKWIFVKDVPNNQ-LRHIRLENNDNK-PVTNSRDTQEVP------------------------LEKAKQVLKII
	tr|A0A1D5P778|A0A1D5P778	GgYTHDF2	GR------------VFIIKSYSEDD--IHR--SIKYNIWCSTEHGNKRLDAA---YR---SMNGK--GP-----VYLLFS---VNGSGHFC------------------------------------GVAEM---KSAVDYNTCA-GVWS-QD----------------------------------KW-KGRFDVRWIFVKDVPNSQ-LRHIRLENNENK-PVTNSRDTQEVP------------------------LEKAKQVLKII
	XP_025005417	GgYTHDC1	AR------------FFLIKSNNHEN--VSL--AKAKGVWSTLPVNEKKLNAA---FR---SARS--VILIFS---VRESGKFQ------------------------------------GFARL---SSESHHGGSP-IHWV-LP---------------------AGMNAK-------ML-GGVFKIDWICRRELPFTK-SAHLTNPWNEHK-PVKIGRDGQEIE------------------------PECGTQLCLLF
	XP_004949328	GgYTHDC2	
Mus musculus	sp|P59326|YTHD1	MmYTHDF1	GR------------VFIIKSYSEDD--IHR--SIKYSIWCSTEHGNKRLDGA---FR---SMSSK--GP-----VYLLFS---VNGSGHFC------------------------------------GVAEM---KSPVDYGTSA-GVWS-QD----------------------------------KW-GKGFDVKWIFVKDVPNNQ-LRHIRLENNDNK-PVTNSRDTQEVP------------------------LEKAKQVLKII
	sp|Q8BYK6|YTHD3	MmYTHDF3	GR------------VFIIKSYSEDD--IHR--SIKYSIWCSTEHGNKRLDAA---YR---SLNGK--GP-----LYLLFS---VNGSGHFC------------------------------------GVAEM---KSVVDYNAYA-GVWS-QD----------------------------------KW-GKGFEVKWIFVKDVPNNQ-LRHIRLENNDNK-PVTNSRDTQEVP------------------------LEKAKQVLKII
	sp|Q91YT7|YTHD2	MmYTHDF2	GR------------VFIIKSYSEDD--IHR--SIKYNIWCSTEHGNKRLDAA---YR---SMNGK--GP-----VYLLFS---VNGSGHFC------------------------------------GVAEM---KSAVDYNTCA-GVWS-QD----------------------------------KW-KGRFDVRWIFVKDVPNSQ-LRHIRLENNENK-PVTNSRDTQEVP------------------------LEKAKQVLKII
	sp|E9Q5K9|YTHDC1	MmYTHDC1	AR------------FFLIKSNNHEN--VSL--AKAKGVWSTLPVNEKKLNLA---FR---SARS--VILIFS---VRESGKFQ------------------------------------GFARL---SSESHHGGSP-IHWV-LP---------------------AGMSAK-------ML-GGVFKIDWICRRELPFTK-SAHLTNPWNEHK-PVKIGRDGQEIE------------------------LECGTQLCLLF
	sp|B2RR83|YTHDC2	MmYTHDC2	IR------------YFIMKSSNLRN--LEI--SQQKGIWSTTPSNERKLNRA---FW---ESSM--VYLVFS---VQGSGHFQ------------------------------------GFSRM---SSEIGREKSQ------------------------------DWGSA-------GL-GGVFKVEWIRKESLPFQF-AHHLLNPWNDNK-KVQISRDGQELE------------------------PQVGEQLLQLW
Macaca fascicularis	tr|A0A2K5U357|A0A2K5U357	MfYTHDF1	GR------------VFIIKSYSEDD--IHR--SIKYSIWCSTEHGNKRLDSA---FR---CMSSK--GP-----VYLLFS---VNGSGHFC------------------------------------GVAEM---KSPVDYGTSA-GVWS-QD----------------------------------KW-GKGFDVKWIFVKDVPNNQ-LRHIRLENNDNK-PVTNSRDTQEVP------------------------LEKAKQVLKII

	tr|A0A2K5VFW1|A0A2K5VFW1	MfYTHDF3	GR------------VFIIKSYSEDD--IHR--SIKYSIWCSTEHGNKRLDAA---YR--SLNGK---GP-----LYLLFS---VNGSGHFC--------------------------------------GVAEM---KSVVDYNAYA-GVWS-QD------------------------------------KW-KGKFEVKWIFVKDVPNNQ-LRHIRLENNDNK-PVTNSRDTQEVP--------------------------LEKAKQVLKII
	sp|Q4R5D9|YTHD2	MfYTHDF2	GR------------VFIIKSYSEDD--IHR--SIKYNIWCSTEHGNKRLDAA---YR--SMNGK---GP-----VYLLFS--VNGSGHFC--------------------------------------GVAEM---KSAVDYNTCA-GVWS-QD------------------------------------KW-KGRFDVRWIFVKDVPNSQ-LRHIRLENNENK-PVTNSRDTQEVP--------------------------LEKAKQVLKII
	XP_005555239	MfYTHDC1	AR------------FFLIKSNNHEN--VSL--AKAKGVWSTLPVNEKKLNLA---FR--SARS--VILIFS---VRESGKFQ--------------------------------------GFARL---SSESHHGGSP-IHWV-LP----------------------AGMSAK-------ML-GGVFKIDWICRRELPFTK-SAHLTNPWNEHK-PVKIGRDGQEIE--------------------------LECGTQLCLLF
	EHH54445	MfYTHDC2	VR------------YFIMKSSNLRN--LEI--SQQKGIWSTTPSNERKLNRA---FW--ESSM--VYLVFS---VQGSGHFQ--------------------------------------GFSRM---SSEIGREKSQ--------------------------------DWGSA-------GL-GGVFKVEWIRKESLPFQF-AHHLLNPWNDNK-KVQISRDGQELE--------------------------PQVGEQLLQLW
Pongo abelii	tr|A0A2J8RB63|A0A2J8RB63	PaYTHDF1	GR------------VFIIKSYSEDD--IHR--SIKYSIWCSTEHGNKRLDSA---FR---CMSSK---GP-----VYLLFS---VNGSGHFC--------------------------------------GVAEM---KSPVDYGTSA-GVWS-QD------------------------------------KW-KGKFDVKWIFVKDVPNNQ-LRHIRLENNDNK-PVTNSRDTQEVP--------------------------LEKAKQVLKII
	NP_001124613_YTHDF3	PaYTHDF3	GR------------VFIIKSYSEDD--IHR--SIKYSIWCSTEHGNKRLDAA---YR--SLNGK---GP-----LYLLFS---VNGSGHFC--------------------------------------GVAEM---KSVVDYNAYA-GVWS-QD------------------------------------KW-KGKFEVKWIFVKDVPNNQ-LRHIRLENNDNK-PVTNSRDTQEVP--------------------------LEKAKQVLKII
	XP_024105083	PaYTHDF2	GR------------VFIIKSYSEDD--IHR--SIKYNIWCSTEHGNKRLDAA---YR--SMNGK---GP-----VYLLFS--VNGSGHFC--------------------------------------GVAEM---KSAVDYNTCA-GVWS-QD------------------------------------KW-KGRFDVRWIFVKDVPNSQ-LRHIRLENNENK-PVTNSRDTQEVP--------------------------LEKAKQVLKII
	XP_024101338	PaYTHDC1	AR------------FFLIKSNNHEN--VSL--AKAKGVWSTLPVNEKKLNLA---FR--SARS--VILIFS---VRESGKFQ--------------------------------------GFARL---SSESHHGGSP-IHWV-LP----------------------AGMSAK-------ML-GGVFKIDWICRRELPFTK-SAHLTNPWNEHK-PVKIGRDGQEIE--------------------------LECGTQLCLLF
	sp|Q5R746|YTHDC2	PaYTHDC2	VR------------YFIMKSSNLRN--LEI--SQQKGIWSTTPSNERKLNRA---FW--ESSM--VYLVFS---VQGSGHFQ--------------------------------------GFSRM---SSEIGREKSQ--------------------------------DWGSA-------GL-GGVFKVEWIRKESLPFQF-AHHLLNPWNDNK-KVQISRDGQELE--------------------------PQVGEQLLQLW
Poecilia formosa	XP_007553615	PfYTHDF1a	GR------------VFIIKSYSEDD--IHR--SIKYSIWCSTEHGNKRLDAA---YR--AMTAK---GP-----VYLLFS---VNGSGHFC--------------------------------------GVAEM---RSPVDYGTSA-GVWA-QD------------------------------------KW-KGKFDVNWLFVKDVPNSQ-LRHIRLENNDNK-PVTNSRDTQEVP--------------------------LEKAKQVLKII
	XP_007557688	PfYTHDF1b	GR------------VFIIKSYSEDD--IHR--SIKYSIWCSTEHGNKRLDSA---FR---AMNGK---GP-----VYLLFS---VNGSGHFC--------------------------------------GVAEM---RSPVDYGTSA-GVWA-QD------------------------------------KW-KGKFDVDWLFVKDVPNSQ-LRHIRLENNDNK-PVTNSRDTQEVP--------------------------LEKAKQVLKII
	XP_007554569	PfYTHDF3	GR------------VFIIKSYSEDD--IHR--SIKYSIWCSTEHGNKRLDSA---YR---SLCNK---GP-----LYLLFS---VNGSGHFC--------------------------------------GVAEM---RSPVDYNAYA-GVWS-QD------------------------------------KW-KGKFEVKWIFIKDVPNNQ-LRHIRLENNDNK-PVTNSRDTQEVP--------------------------LEKAKQVLKVI

	XP_007561175	PfYTH DF2	GR------------VFIIKSYSEDD--IHR--SIKYNIWCSTEHGNKRLDAA---YR---SLAGK---------------------------------<br>---------------GP-----LYLLFS--VNGSGHFC------------------------------------GVAEM---RSPVDYNTSA-<br>GVWS-QD----------------------------------KW-KGRFDVRWIFVKDVPNSQ-LRHIRLENNENK-PVTNSRDTQEVP------------------------LDKARQVLKII
	XP_007541776	PfYTH DC1	AR------------FFLIKSNNHEN--VSL--AKAKGVWSTLPVNEKKLNAA---FQ---SARS---------------------------------<br>------------------------VILVFS--VRESGKFQ------------------------------------GFARL---SSESHHDGSP-<br>IHWV-LP---------------------AGMNAK-------ML-GGVFKIDWLCRRELPFIK-TAHLFNPWNDNK-PVKIGRDGQEIQ------------------------PKVGAQLCALF
Fundulus heteroclitus	tr|A0A146VPC4|A0A146VPC4	FhYTH DF1a	GR------------VFIIKSYSEDD--IHR--SIKYSIWCSTEHGNKRLDAA---YR---AMNSK---------------------------------<br>---------------GP-----VYLLFS--VNGSGHFC------------------------------------GMAEM---RSPVDYGTSA-<br>GVWA-QD----------------------------------KW-KGKFDVNWLFVKDVPNSQ-LRHIRLENNDNK-PVTNSRDTQEVP------------------------LEKAKQVLKII
	tr|A0A146N3Z6|A0A146N3Z6	FhYTH DF1b	GR------------VFIIKSYSEDD--IHR--SIKYSIWCSTEHGNKRLDSA---FR---AMNAK---------------------------------<br>---------------GP-----VYLLFS--VNGSGHFC------------------------------------GVAEM---RSPVDYGTSA-<br>GVWA-QD----------------------------------KW-KGKFDVDWLFVKDVPNSQ-LRHIRLENNDNK-PVTNSRDTQEVP------------------------LEKAKQVLKII
	tr|A0A146XRV8|A0A146XRV8	FhYTH DF3	GR------------VFIIKSYSEDD--IHR--SIKYSIWCSTEHGNKRLDAA---YR---SLCNK---------------------------------<br>--------------GP-----LYLLFS--VNGSGHFC------------------------------------GVAEM---RSPVDYNAYA-<br>GVWS-QD----------------------------------KW-KGKFEVKWIFIKDVPNNQ-LRHIRLENNDNK-PVTNSRDTQEVP------------------------LEKAKQVLKVI
	XP_012720020	FhYTH DF2	GR------------VFIIKSYSEDD--IHR--SIKYNIWCSTEHGNKRLDAA---YR---SLAGK---------------------------------<br>---------------GP-----LYLLFS--VNGSGHFC------------------------------------GVAEM---RSPVDYNTSA-<br>GVWS-QD----------------------------------KW-KGRFDVRWIFVKDVPNSQ-LRHIRLENNENK-PVTNSRDTQEVP------------------------LDKARQVLKII
	XP_021169391	FhYTH DC1	AR------------FFLIKSNNHEN--VSL--AKAKGVWSTLPVNEKKLNSA---FQ---AARS---------------------------------<br>------------------------VILVFS--VRESGKFQ------------------------------------GFARL---SSQSHHGGSP-<br>IHWV-LP---------------------AGMNAK-------ML-GGVFRIDWLCRRELPFIK-TAHLSNPWNDHK-PVKIGRDGQEIA------------------------PKIGAQLCALF
	XP_012707270	FhYTH DC2	VR------------YFIMKSNSIRN--IEI--SQQKGIWSTTPSNETKLTQA---FL---ANSF-------------------------------------<br>-------------------IILVFS--VQGSGHFC------------------------------------GYARM---TSMVRQDSCQ----------<br>---------------------DWGFT-------GL-GGVFSVEWIHKESLPFPF-TQHILNPWNDNK-RVQVSRDGQELE------------------------PQAGSQLLSLW
Nothobranchius furzeri	XP_015824020	NfYTH DF1a	GR------------VFIIKSYSEDD--IHR--SIKYSIWCSTEHGNKRLDSA---YR---AMNSK---------------------------------<br>---------------GP-----VYLLFS--VNGSGHFC------------------------------------GVAEM---CSPVDYGTSA-<br>GVWA-QD----------------------------------KW-KGKFDVNWLFVKDVPNSQ-LRHIRLENNDNK-PVTNSRDTQEVP------------------------LEKAKQVLKII
	XP_015823582	NfYTH DF1b	GR------------IFIIKSYSEDD--IHR--SIKYSIWCSTEHGNKRLDSA---FR---AMNGK---------------------------------<br>--------------GP-----VYLLFS--VNGSGHFC------------------------------------GVAEM---RSPVDYGTSA-<br>GVWA-QD----------------------------------KW-KGKFDVDWLFVKDVPNSQ-LRHIRLENNDNK-PVTNSRDTQEVP------------------------LEKAKQVLKII
	XP_015810707	NfYTH DF3	GR------------VFIIKSYSEDD--IHR--SIKYSIWCSTEHGNKRLDSA---YR---SLGNK---------------------------------<br>--------------GP-----LYLLFS--VNGSGHFC------------------------------------GVAEM---RSPVDYNAYA-<br>GVWS-QD----------------------------------KW-KGKFEVKWIFVKDVPNNQ-LRHIRLENNDNK-PVTNSRDTQEVP------------------------LEKAKQVLKVI
	XP_015825357	NfYTH DF2	GR------------VFIIKSYSEDD--IHR--SIKYNIWCSTEHGNKRLDAA---YR---SLSGK---------------------------------<br>--------------GP-----LYLLFS--VNGSGHFC------------------------------------GVAEM---RSPVDYNTSA-<br>GVWS-QD----------------------------------KW-KGRFDVRWVFVKDVPNSQ-LRHIRLENNENK-PVTNSRDTQEVP------------------------LDKARQVLKII

	XP_015798763	NfYTHDC1	AR------------FFLIKSNNHEN--VSL--AKAKGVWSTLPVNEKKLNAA---FR--SARS---VILVFS--VRESGKFQ-------------------------------------GFARL---ASESHHGGSP-IHWV-LP----------------------AGMNAK-------ML-GGVFKINWLCRRELPFIK-TAHLSNPWNEFK-PVKIGRDGQEIQ-------------------------PTVGAQLCALF
	XP_015829646	NfYTHDC2	VR------------YFVMKSSNARN--IDI--SQEKGIWSTTPSNETKLTKA---FM--GHNL--IILIFS--VQGSGHFQ-------------------------------------GYAHM---TSAASQESCQ-------------------------------DWGFV-------GL-GGGFSVEWVHKESLPFHL-TQHILNPWNDNK-KVQISRDGQELE-------------------------PQTGSQLVLLW
Carassius auratus	XP_026108152	CaYTHDF1	GR------------VFIIKSYSEDD--IHR--SIKYSIWCSTEHGNKRLDTA---FR--GINGK---GP-----VYLLFS--VNGSGHFC-------------------------------------GVAEM---RSPVDYGTSA-GVWA-QD-----------------------------------KW-KGKFDVDWLFVKDVPNSQ-LRHIRLENNDNK-PVTNSRDTQEVP-------------------------LEKAKQVLKII
	XP_026057128	CaYTHDF3	GR------------VFIIKSYSEDD--IHR--SIKYSIWCSTEHGNKRLDGA---YR--SLSAK--GP-----LYLLFS--VNGSGHFC-------------------------------------GVAEM---MSTVDYNAYA-GVWS-QD-----------------------------------KW-KGKFEVKWIFVKDVPNNQ-LRHIRLENNDNK-PVTNSRDTQEVP-------------------------LEKAKQVLKII
	XP_026141540	CaYTHDF2	GR------------IFIIKSYSEDD--IHR--SIKYNIWCSTEHGNKRLDAA---YR--SLANK--GP-----LYLLFS--VNGSGHFC-------------------------------------GVAEM---RSPVDYNTCA-GVWS-QD-----------------------------------KW-KGRFDVRWIFVKDVPNSQ-LRHIRLENNENK-PVTNSRDTQEVP-------------------------LDKARQVLKII
	XP_026110814	CaYTHDC1	AR------------FFLIKSNNHEN--VSL--AKAKGVWSTLPVNEKKLNAA---FR--SARS---VMLIFS--VRESGKFQ-------------------------------------GFARL---SSESHHGGSP-IHWV-LP----------------------AGMNAK-------ML-GGVFKIDWICRRELPFTK-TAHLSNPWNEHK-PVKIGRDGQEIE-------------------------LDCGTQLCMLF
	XP_026099624	CaYTHDC2	IR------------YFIMKSSNMRN--IDL--SQQRGIWSTTPNNEHKLTRA---FI--ESSA---VFLIFS--VQGSGHFQ-------------------------------------GYARM---ASAVSGERCL--------------------------------DWGSS-------GL-GGVFGVEWIRKESLAFQL-TQQLLNPWNDNK-RVQISRDAQELE-------------------------PQTGSQLLQLW
Salmo salar	tr|A0A1S3R6T3|A0A1S3R6T3	SsYTHDF1a	GR------------VFIIKSYSEDD--IHR--SIKYSIWCSTEHGNKRLDGA---FR--GLNFK--GP-----VYLLFS--VNGSGHFC-------------------------------------GVAEM---RSPVDYGTSA-GVWA-QD-----------------------------------KW-KGKFDVDWLFVKDVPNSQ-LRHIRLENNDNK-PVTNSRDTQEVP-------------------------LEKAKQVLKII
	tr|A0A1S3LGZ9|A0A1S3LGZ9	SsYTHDF1b	GR------------VFIIKSYSEDD--IHR--SIKYSIWCSTEHGNKRLDGA---FR--SLNGK--GP-----VYLLFS--VNGSGHFC-------------------------------------GVAEM---RSSVDYGTSA-GVWA-QD-----------------------------------KW-KGKFDVDWLFVKDVPNSQ-LRHIRLENNDNK-PVTNSRDTQEVP-------------------------LEKAKQVMKII
	tr|A0A1S3LMH3|A0A1S3LMH3	SsYTHDF1c	GR------------VFIIKSYSEDD--IHR--SIKYSIWCSTEHGNKRLDSA---FR--AINAK---GP-----VYLLFS--VNGSGHFC-------------------------------------GVAEM---LSPVDYGTSA-GVWA-QD-----------------------------------KW-KGKFDVDWLFVKDVPNSQ-LRHIRLENNDNK-PVTNSRDTQEVP-------------------------LEKAKQVLKII
	tr|A0A1S3MGD6|A0A1S3MGD6	SsYTHDF1d	GR------------VFIIKSYSEDD--IHR--SIKYSIWCSTEHGNKRLDSA---FR--AINAK---GP-----VYLLFS--VNGSGHFC-------------------------------------GVAEM---LSPVDYGTSA-GVWA-QD-----------------------------------KW-KGKFDVDWLFVKDVPNSQ-LRHIRLENNDNK-PVTNSRDTQEVP-------------------------LEKAKQVLKII
	XP_014015013	SsYTHDF3a	GR------------VFIIKSYSEDD--IHR--SIKYSIWCSTEHGNKRLDGA---YR--SLGAK--GP-----LYLLFS--VNGSGHFC-------------------------------------GVAEM---RSPVDYNAYA-GVWS-QD-----------------------------------KW-KGKFEVKWAFVKDVPNSQ-LRHIRLENNDNK-PVTNSRDTQEVP-------------------------LEKAKQVLKII

	XP_014036960	SsYTHDF3b	GR------------VFIIKSYSEDD--IHR--SIKYSIWCSTEHGNKRLDGA---YR--SLGAK----------------------------------<br>--------------GP-----LYLLFS---VNGSGHFC-------------------------------------GVAEM---RSPVDYNSYA-<br>GVWS-QD-----------------------------------KW-KGKFEVKWAFVKDVPNNQ-LRHIRLENNDNK-PVTNSRDTQEVP-------------------------LEKAKQVLKII
	XP_013979848	SsYTHDF2a	GR------------VFIIKSYSEDD--IHR--SIKYNIWCSTEHGNKRLDAA---YR--SLGAK---------------------------------<br>---------------GP-----LYLLFS---VNGSGHFC-------------------------------------GVAEM---RSPVDYNTCA-<br>GVWS-QD-----------------------------------KW-KGRFDVRWIFVKDVPNSQ-LRHIRLENNENK-PVTNSRDTQEVP-------------------------LDKARQVLKII
	XP_014065583	SsYTHDF2b	GR------------VFIIKSYSEDD--IHR--SIKYNIWCSTEHGNKRLDAA---YR--SLGAK---------------------------------<br>---------------GP-----LYLLFS---VNGSGHFC-------------------------------------GVAEM---RSPVDYNTCA-<br>GVWS-QD-----------------------------------KW-KGRFDVRWIFVKDVPNSQ-LRHIRLENNENK-PVTNSRDTQEVP-------------------------LDKARQVLKII
	XP_013994485	SsYTHDC1a	AR------------FFMIKSNNHEN--VSL--AKAKGVWSTLPVNEKKLNAA---FR--SARS--------------------------------<br>------------------------VILVFS---VRESGKFQ-------------------------------------GFARL---SSESHHGGSP-<br>IHWV-LP----------------------AGMNAK-------ML-GGVFKIDWICRRELPFTK-TAHLSNSWNEHK-PVKIGRDGQEME-------------------------PECGIQLCVMF
	XP_014002672	SsYTHDC1b	TR------------FFLIKSNNHEN--VSL--AKAKGVWSTLPVNEKKLNAA---FR--SARS--------------------------------<br>-----------------------VILVFS---VRESGKFQ-------------------------------------GFARL---SSESHHGGSP-<br>IHWV-LP----------------------AGMNAK-------ML-GGVFKIHWICRRELPFTK-TAHLSNPWNEHK-PVKIGRDGQEIE-------------------------PGCGMQLCMLF
	XP_013984132	SsYTHDC2	VR------------YFIMKSSNLRN--LDI--SQQKGIWSTTPSNELKLNRA---FL--ESSL------------------------------------<br>--------------------VFLVFS---VQGSGHFQ-------------------------------------GYARM---TSVIGQERCQ---------<br>----------------------DWGST-------GL-GGVFSVEWTRKESLPFQC-THQLLNPWNDNK-KVQISRDGQELE-------------------------PQAGGQLLQLW
Oncorhynchus mykiss	XP_021422541	OmYTHDF1a	GR------------VFIIKSYSEDD--IHR--SIKYSIWCSTEHGNKRLDGA---FR--SLNGK----------------------------------<br>--------------GP-----VYLLFS---VNGSGHFC-------------------------------------GVAEM---RSSVDYGTSA-<br>GVWA-QD-----------------------------------KW-KGKFDVDWLFVKDVPNSQ-LRHIRLENNDNK-PVTNSRDTQEVP-------------------------LEKAKQVMKII
	CDQ89726	OmYTHDF1b	GR------------VFIIKSYSEDD--IHR--SIKYSIWCSTEHGNKRLDGA---FR--SLNGK----------------------------------<br>--------------GP-----VYLLFS---VNGSGHFC-------------------------------------GVAEM---RSSVDYGTSA-<br>GVWA-QD-----------------------------------KW-KGKFDVDWLFVKDVPNSQ-LRHIRLENNDNK-PVTNSRDTQEVP-------------------------LEKAKQVMKII
	XP_021422899	OmYTHDF1c	GR------------VFIIKSYSEDD--IHR--SIKYSIWCSTEHGNKRLDSA---FR--AINAK-----------------------------------<br>-------------GP-----VYLLFS---VNGSGHFC-------------------------------------GVAEM---LSPVDYGTSA-<br>GVWA-QD-----------------------------------KW-KGKFDVDWLFVKDVPNSQ-LRHIRLENNDNK-PVTNSRDTQEVP-------------------------LEKAKQVLKII
	XP_021472143	OmYTHDF1d	GR------------VFIIKSYSEDD--IHR--SIKYSIWCSTEHGNKRLDSA---FR--AISAK-----------------------------------<br>-------------GP-----VYLLFS---VNGSGHFC-------------------------------------GVAEM---LSPVDYGTSA-<br>GVWA-QD-----------------------------------KW-KGKFDVDWLFVKDVPNSQ-LRHIRLENNDNK-PVTNSRDTQEVP-------------------------LEKAKQVLKII
	XP_021475945	OmYTHDF3a	GR------------VFIIKSYSEDD--IHR--SIKYSIWCSTEHGNKRLDGA---YR--SLGAK----------------------------------<br>--------------GP-----LYLLFS---VNGSGHFC-------------------------------------GVAEM---RSPVDYNAYA-<br>GVWS-QD-----------------------------------KW-KGKFEVKWAFVKDVPNSQ-LRHIRLENNDNK-PVTNSRDTQEVP-------------------------LEKAKQVLKII
	XP_021418082	OmYTHDF3b	GR------------VFIIKSYSEDD--IHR--SIKYSIWCSTEHGNKRLDGA---YR--SLGAK----------------------------------<br>--------------GP-----LYLLFS---VNGSGHFC-------------------------------------GVAEM---RSPVDYNSYA-<br>GVWS-QD-----------------------------------KW-KGKFEVKWAFVKDVPNNQ-LRHIRLENNDNK-PVTNSRDTQEVP-------------------------LEKAKQVLKII

	XP_021440084	OmYT HDF2a	GR------------VFIIKSYSEDD--IHR--SIKYNIWCSTEHGNKRLDAA---YR--SLGAK---GP-----LYLLFS---VNGSGHFC--------------------------------------GVAEM---RSPVDYNTCA-GVWS-QD-----------------------------------KW-KGRFDVRWIFVKDVPNSQ-LRHIRLENNENK-PVTNSRDTQEVP-------------------------LDKARQVLKII
	XP_021430447	OmYT HDF2b	GR------------VFIIKSYSEDD--IHR--SIKYNIWCSTEHGNKRLDAA---YR--SLGAK---GP-----LYLLFS---VNGSGHFC--------------------------------------GVAEM---RSPVDYNTCA-GVWS-QD-----------------------------------KW-KGRFDVRWIFVKDVPNSQ-LRHIRLENNENK-PVTNSRDTQEVP-------------------------LDKARQVLKII
	XP_021457161	OmYT HDC1a	AR------------FFLIKSNNHEN--VSL--AKAKGVWSTLPVNEKKLNAA---FR--SARS---VILVFS---VRESGKFQ--------------------------------------GFARL---SSESHHGGSP-IHWV-LP-----------------------AGMNAK-------ML-GGVFKIHWICRRELPFTK-TAHLSNPWNEHK-PVKIGRDGQEME-------------------------PGCGMQLCVTF
	XP_021478131	OmYT HDC1b	SS------------LFAYTN--------------------SMPPVCSVLNMI--CPP--VCSVLN---MVCPPVRA--------------------------------------GFARL---SSESHHGGSP-IHWV-LP----------------------AGMNAK-------ML-GGVFKIDWICRRELPFTK-TAHLSNSWNEHK-PVKIGRDGQEME-------------------------PGCGMQLCVTF
	XP_021446650	OmYT HDC2	VR------------YFIMKSSNLRN--IDI--SQQKGIWSTTPSNELKLNRA---FL--ESSL--VFLVFS---VQGSGHFQ--------------------------------------GYARM---TSVIGQERCQ-------------------------------DWGST-------GL-GGVFSVEWTRKESLPFQC-THQLLNPWNDNK-KVQISRDGQELE-------------------------PQAGGQLLQLW
Danio rerio	tr|Q802Z0|Q802Z0	DrYTH DF1	GR------------VFIIKSYSEDD--IHR--SIKYSIWCSTEHGNKRLDSA---FR--AINGK---GP-----VYLLFS---VNGSGHFC--------------------------------------GVAEM---RSPVDYGTSA-GVWA-QD-----------------------------------KW-KGKFDVDWLFVKDVPNSQ-LRHIRLENNDNK-PVTNSRDTQEVP-------------------------LEKAKQVLKII
	tr|E7FAP0|E7FAP0	DrYTH DF3	GR------------VFIIKSYSEDD--IHR--SIKYSIWCSTEHGNKRLDGA---YR--SLSAK--GP-----LYLLFS---VNGSGHFC--------------------------------------GVAEM---KSTVDYNAYA-GVWS-QD-----------------------------------KW-KGKFEVKWIFVKDVPNNQ-LRHIRLENNDNK-PVTNSRDTQEVP-------------------------LEKAKQVLKII
	NP_956544	DrYTH DF2	GR------------VFIIKSYSEDD--IHR--SIKYNIWCSTEHGNKRLDAA---YR--SLANK---GP-----PYLLFS---VNGSGHFC--------------------------------------GVAEM---RSPVDYNTCA-GVWS-QD-----------------------------------KW-KGRFDVRWIFVKDVPNSQ-LRHIRLENNENK-PVTNSRDTQEVP-------------------------LDKARQVLKII
	NP_001007411	DrYTH DC1	AR------------FFLIKSNNHEN--VSL--AKAKGVWSTLPVNEKKLNAA---FR--AARS---VVLIFS---VRESGKFQ--------------------------------------GFARL---SSESHHGGSP-IHWV-LP-----------------------AGMNAK-------ML-GGVFKIDWICRRELPFTK-TAHLANPWNEHK-PVKIGRDGQEIE-------------------------PECGTELCMLF
	XP_021332046	DrYTH DC2	VR------------YFIMKSSNPRN--IEL--SQQRSVWSTTPNNEQKLNRA---FQ--NSSA---VFLVFS---VQGSGHFQ--------------------------------------GYARM---TSAVSSERCL-------------------------------DFGSS-------GL-GGVFSVQWIHTESLAFQL-THKLLNPWNDNK-KVQISRDAQELE-------------------------PHTGNQLLQLW
Branchiostoma floridae	XP_002601468	BfYTH DF	SR------------FFVIKSYSEDD--IHR--SIKYHIWCSTEHGNKRLDQA---FR--ERKGK---GP-----IYLFFS---VNGSGHFC--------------------------------------GVAQM---MSEVDYNADT-GVWS-QE-----------------------------------KW-KGKLEVKWIFVKDVPNSQ-LRHIRLENNENK-PVTNSRDTQEVP-------------------------PEKGKQVLKIL
	XP_002609689	BfYTH DC2	NR------------YFILKCNNQRN--LDI--AMNQSIWATTPSNEKKLNKA---FK--DCQN---VYLVFS---VQGSGHFQ--------------------------------------GYARM---ASSISKDKVP-------------------------------EFSSA-------SL-GGAFQIEWIKRMSIPFQA-AHHLLNPWNENK-KVQISRDGQEIE-------------------------PQVGEQLLKAW

Ciona intestinalis	XP_002130883	CiYTHDF	AR------------FFVIKSYSEDD--IHR--SIKYNIWCSTDHGNKRLDAA---FR--EQQGH---------------------------------------<br>----------------GP-----VILLYS---VNGSGHFC------------------------------------GVAEM---LTQIDYSKRA-<br>GVWA-QD-----------------------------------KW-KGKFQVKWIYAKDVPNSQ-LRHIRLENNENK-PVTNSRDTQEVP------------------------AEKGRQVLKII
	XP_002127950	CiYTHDC1	AH------------FYLIKSNNHEN--VAL--AKARGVWSTPPSNEAKLNRS---FR---EARN-------------------------------------<br>------------------------VILIYS---VRESGAFQ-------------------------------------GFARL---ATEAKHNLSP-<br>IDWV-LP---------------------AGLSAK-------AL-GGVFKIDWLCKRELSFAK-TTDIYNTFNGNK-PVKIGRDGQEVE------------------------PNAGKVLCLEF
Strongylocentrotus purpuratus	XP_011684124	SpuYTHDF	SR------------IFIIKSYSEDD--IHR--SIKYGIWCSTEHGNKRLDAA---MR---ERQSK--<br>--------------GP-----VYLIYS---VNGSGHFC-------------------------------------GVAEM---MSEVDYTTNT-<br>GVWA-QD-----------------------------------KW-KGRFDVRWVYVKDVPNSQ-LRHIRLENNDNK-PVTNSRDTQEVL------------------------LDKAKQVMKII
	NW_022145609	SpuYTHDC1	AR------------YFLIKSNNHEN--IAL--AKAKGVWSTLPYNEQRLNAA---FR--EARN-------------------------------------<br>------------------------VLLIFS---VKESGKFQ-------------------------------------GFARM---RCESRRDGQP-<br>INWV-LP---------------------NGMNRS-------IL-GGVFKVDWITRNELPFTK-TTHLYNPWNDSK-PVKIGRDGQEIE------------------------SKCGESVCRLF
Acanthaster planci	XP_022109588	ApYTHDF	SR------------MFIIKSYSEDD--IHR--SIKYGIWCSTEHGNKRLDSA---FR--ERANK--<br>--------------GP-----VYLLYS--VNGSGHFC-------------------------------------GVAEM---MSPVDYNVNT-<br>GVWM-QD-----------------------------------KW-KGRFDVRWVYVKDVPNSQ-LRHIRLENNDNK-PVTNSRDTQEVS------------------------LDKGRQVVKII
	XP_022105412	ApYTHDC1	AR------------YFLIKSNNHEN--VAL--AKARGVWSTLPNNEQMLNQA---FR---ESRN-------------------------------------<br>-------------------------VLLIFS---VKESGKFQ-------------------------------------GFARM---RSESRRDGPN-<br>INWV-LP---------------------NGMSRS-------VL-GGVFKIDWITRQELAFSK-TGHLFNSWNDNK-PVKIGRDGQEVE------------------------TRCGEALCRLF
	XP_022100852	ApYTHDC2	VR------------FFIVKCNNAMN--IDL--SYSNGLWCVSPSTGQILSNA---FK--TSQA---<br>----------------------IFLIFS--VQGSGQFE-------------------------------------GFGRM---TSDLQYKQTI--DW---<br>------------------------RDSCM-------RN-GAEFGVQWMKRASVSFDQ-TRAILNPWNDNL-RVHVSRDGQELE------------------------PSVGQNLLQLW
Lingula anatina	XP_013381791	LaYTHDF	SR------------FFIIKSYSEDD--IHR--SIKYSIWCSTDHGNKRLDTA---YR---QQEGK--<br>--------------GP-----VYLLFS---VNGSGHFC-------------------------------------GVAQM---LSPIDYNRTS-<br>GVWA-QD-----------------------------------KW-KGQFEVKWIYVKDVPNSQ-LRHIRLENNENK-PVTNSRDTQEVP------------------------LEKGKQVMKIL
	XP_013407344	LaYTHDC1	AT------------YFIIKSNNHEN--VAL--AKAKGVWSTPPQNEFRINKA---YR---ESRN-------------------------------------<br>---------------------VILIFS---VRESGKFQ-------------------------------------GYARV---ASESDKDHPP-<br>IRWV-LP---------------------PGLSAK-------AL-SGVFKLDWVTRRDLPFTK-TAHLLNPWNDNK-PVKIGRDGQPVE------------------------PRCGEALCRMF
	XP_013406748	LaYTHDC2	CC------------YFVIKCNNDKQ--LDA--SFNRGIWGPQIQTERKLVKA---FR---EGKV---<br>-----------------------VYLIFS---VQGSGHFQ-------------------------------------GFAKM---LAEPIKEKAP-------<br>------------------------EFNYP-------GL-GGTLSIQWIKRANLPFKN-THHLTNPWNENR-RVQISRDGQEIE------------------------PSIGAQLIQLW
Octopus bimaculoides	XP_014787225	ObYTHDF	AR------------FFIIKSYSEDD--IHR--SIKYNIWCSTEHGNKRLDGA---YR--EREGK--<br>--------------GP-----VYLFYS---VNGSGHFC-------------------------------------GMAQM---MSPVDYSTNS-<br>GVWA-QD-----------------------------------KW-KGQFEVKWIYVKDVPNSQ-LRHIRLENNENK-PVTNSRDTQEVP------------------------PEKGKQVLKIL
	XP_014768358	ObYTHDC1	AR------------FFLIKSNNHEN--VAL--AKAKGVWSTPPQNEARLNQA---FR---EADN-------------------------------------<br>-------------------------VILVFS---VKESGKFQ-------------------------------------GFARL---SAESTRDHPP-<br>IRWV-LP---------------------PGLSAR-------AL-SGVFKLDWINRRDLQFTK-TTHLHNPWNENK-YVKIGRDGQEIE------------------------PTVGEALCKLF

	XP_014778535	ObYTHDC2	TSNNQRN--LDT--SFSKSIWTTSPNNEKKLNKA---FQ--DGKT--VYLIFS--IQGSGCFQ------------------------------------GYAKM---LSTFSKDRSA------------------------------EYSMP-------GL-GATCTVDWLKRGNIPFHN-VQHLSNPWNDNK-RIQVSRDCQEIE------------------------PFVGETLLKQW
Mizuhopecten yessoensis	OWF49575	MyYTHDF	AR------------FFVIKSYSEDD--IHR--SIKYSIWCSTEHGNKRLDSA---YK--ERESK--GP-----IYLFYS--VNGSGHFC------------------------------------GMAQM---MSTLDYNRSP-GVWA-QD----------------------------------KW-KGQLDVKWIYVKDVPNSQ-LRHIRLENNENK-PVTNSRDTQEVP------------------------PEKGKQVLKLL
	XP_021370725	MyYTHDC1	AR------------YFLIKSNNHEN--VAL--AKAKGVWSTPPQNEAKLNQA---FR--SCDN--VILIFS--VKESGKFQ------------------------------------GFARI---VEESTKDHPP-IRWV-LP---------------------PGLSAR-------AL-SGVFKLDWINRRELSFTK-TSHLHNSWNDNK-PVKIGRDGQEIE------------------------ARCGETVCKMF
	XP_021349616	MyYTHDC2	FFVMKCNNQSK--VDL--SMNKGVWATNKSNNKKLNSV---FQ---EGNT---VYLVFS--VQGSGHFQ------------------------------------GYAKM---KSEFVDKKVQ------------------------------DSSYH-------GL-DYCCDVEWIKRANLSFQS-TLQLLNPWDDNR-KVQTSRDGQEIE------------------------PHVGETLIKLW
Pomacea canaliculata	XP_025090552	PcYTHDF	AR------------FFIIKSYSEDD--IHR--SIKYNIWCSTEHGNKRLDQA---FR--EREGK--GP-----VYLIYS--VNGSGHFC------------------------------------GIAQM---VSPVDYNKNS-GVWA-QD----------------------------------KW-KGQFEVKWVYVKDVPNSQ-LRHIRLENNENK-PVTNSRDTQEVP------------------------PEKGKQVLKIL
	XP_025111582	PcYTHDC1	AR------------FFLIKSNNHEN--VAL--AKAKGVWSTPPQNEARLNQA---FR--ECDN--VILVFS--VKESGKFQ------------------------------------GFARL---AAESTKDHPP-IRWV-LP---------------------QGMNSR-------AL-SAVFKLDWVCRRELPFTK-TNHLHNPWNDDK-PVKIGRDGQEID------------------------PRCGESLCKLF
	XP_025077319	PcYTHDC2	CR------------YFVMKCNNQRN--LDI--SQSKGIWATSPANQAKMNRA---FR--DGKT--VYLIFS--IQGSGHFQ------------------------------------GYAKM---LTEIGRHKSP------------------------------DFASP-------GL-TGTFAVEWIKRANIPFMS-THHLYNTWNENR-KVQVSRDGQELE------------------------PGVGEALVKLW
Aplysia californica	XP_005091424	AcaYTHDF	AR------------FFIIKSYSEDD--IHR--SIKYNIWCSTDHGNKRLDQA---FR--EREGK--GP-----VYLIYS--VNGSGHFC------------------------------------GIAQM---MSPVDYNKSA-GVWA-QD----------------------------------KW-KGQFTVKWVYVKDVPNST-LRHIRLENNENK-PVTNSRDTQEVP------------------------PEKGKQVLRIM
	XP_005104889	AcaYTHDC1	AR------------FFLVKSNNHEN--VAL--AKAKGIWSTPPQNEARFNQA---FK--ECEN--VILIFS--VKESGKFQ------------------------------------GFARL---AEESTKDHPP-VRWV-LP---------------------PGMTSR-------AL-SGVFKLDWVSRKDLEFIK-TNHLFNLWNDNK-RVKIGRDGQEIE------------------------PRCGENLCKLF
	XP_005089719	AcaYTHDC2	FFIMKAANHKL--LEI--SQTKNFWATSTGHEGKLSKA---YM--SGRV---VYLIFS--VQGSGHFQ------------------------------------GFAKM---TSSIARTKSP------------------------------DFPSP-------GL-SAIFSVEWLKSAHLPFQL-CHHLLNPWNENK-KVQESRDGQELE------------------------AHVGSQLLKLW
Crassostrea gigas	XP_011456337	CgYTHDF	AR------------FFIIKSYSEDD--IHR--SIKYSIWCSTDHGNKRLDQA---FK--ERDNK--GP-----IYLFFS--VNGSGHFC------------------------------------GMAQM---MSSLDYGKQA-GVWA-QD----------------------------------KW-RGQFEVKWIYVKDVPNSQ-LRHIRLENNENK-PVTNSRDTQEVP------------------------PEKGKSVLKIM
	XP_011447601	CgYTHDC1	AR------------YFLIKSNNHEN--VAL--AKAKGVWSTPPQNEIRLNNA---YK--SCDN--VILIFS--VRESGKFQ------------------------------------GFARI---ADESTKDHPP-IRWV-LP---------------------PGLSAR-------AL-SGVFKLDWINRKELAFTK-TQHLHNAWNDNK-PVKIGRDGQEVE------------------------PRCGEALCKMF

	XP_011425711	CgYTH DC2	VK------------YFVVKPNNQKA--LDI--ALSNSVFATTPKSETKFNKA---IQ--DGKE---VYLIFS--MIDSAQFQ------------------------------------GYAKV---TAQSSQDKCP-------------------------------DMSGD-------GL-GGTFKIEWMKKTPVPFRA-TQNICNSWNENK-KVQYSKDGQELE------------------------YSCGERLIQVL
Biomphalaria glabrata	XP_013083543	BgYTH DF	AR------------FFIIKSYSEDD--IHR--SIKYNIWCSTDHGNKRLDQA---FR--EREGK---GP-----VYLIYS--VNGSGHFC------------------------------------GIAQM---MSPVDYNKSA-GVWA-QD---------------------------------KW-KGQFVVKWVYVKDVPNSQ-LRHIRLENNENK-PVTNSRDTQEVP------------------------PEKGKQVLRIV
	XP_013071333	BgYTH DC1	AR------------FFLVKSNNHEN--VAL--AKAKGVWSTPPQNEARFNQA---FR--ECDN---VILIFS--VKESGKFQ------------------------------------GFARL---SEESTKDHPP-IRWV-LP---------------------PGMTSR-------AL-SGVFRLDWITRRDLDFQK-TNHLFNAWNDNK-RVKIGRDGQEIE------------------------PRCGESLCKLF
	XP_013089748	BgYTH DC2	GR------------YFLMRCNNQKI--VDV--SETKGIWATSVSNETKLNRA---FN--SGKP--VYLIFS--VPGNANFQ------------------------------------GYAKM---TSLISKNRSP-------------------------------DFPGS-------GL-TGTFSVSWVKRATIPFSN-TNFLNNPWNENR-RVHTGRDGQEIE------------------------PKIGLELIKLW
Drosophila hydei	XP_023169635	DhYTH DF	AR------------FFVIKSYSEDD--IHR--SIKYEIWCSTDHGNKRLDDA---FK--ERQKE---GGN-----VLLFFS--VNKSGYFC------------------------------------GMAQM---MTSVDYNSTS-SVWS-QD---------------------------------KW-KGKFKVKWIYVKDVPNGE-LRHIRLENNDNK-SVTHSRDTQEIP------------------------NTKGIEVLQIL
	XP_023173281	DhYTH DC1	TR------------FFLIKSNNSDN--VQL--SKNKSVWATLPQNDANLSQA---FK--EARN---VLLIFS--VNESGKFA------------------------------------GFARM---AAPSRRDIPQ-VAWV-LP---------------------PSISPK-------AL-GGVIELDWICRKELSFNA-TLHLHNTWNEGK-PVKIGRDGQEIE------------------------PKIGAELCRLF
Stomoxys calcitrans	XP_013113692	ScaYTH DF	AR------------FFVIKSYSEDD--IHR--SIKYEIWCSTDHGNKRLDDA---FK--ERHKD---GGN-----ILLFFS--VNGSGHFC------------------------------------GMAQM---MTPVDYNSTS-SVWS-QD---------------------------------KW-KGKFKVKWIYVKDVPNNK-LRHITLENNENK-PVTNSRDTQEVP------------------------NAKGIEVLQIL
	XP_013118835	ScaYTH DC1	TR------------FFLIKSNNADN--VTL--SKNKNVWATLPQNDANLTQA---FK--ESRN---VLLIFS--VNESGKFA------------------------------------GFARM---SASSSRDIPQ-PDWV-LP---------------------SSISAK-------AL-GGVIEIDWICRKELSFNC-TSHLYNSWNEGK-PVKIGRDGQEIE------------------------PKVGAELCRLF
Lucilia cuprina	XP_023298015	LcYTH DF	AR------------FFVIKSYSEVD--VHR--SIKYEIWCSTDHGNKRLDDA---FK--ERSKD---GGN-----ILLFFS--VNGSGHFC------------------------------------GMAQM---MTSVDYNSTS-SVWS-QD---------------------------------KW-RGKFKVKWIYVKDVPNKE-LRHITLENNENK-PVTNSRDTQEVP------------------------HAKGIEVLQIL
	XP_023292455	LcYTH DC1	
Habropoda laboriosa	KOC65487	HlYTH DF	AR------------FFVIKSYSEDD--IHR--SIKYEIWCSTEHGNKRLDQA---YR--EASRE---GAP-----LYLFFS--VNGSGHFC------------------------------------GMAQM---VSPVDYKSNS-SVWS-QD---------------------------------KW-KGQFRVRWIYVKDVPNVQ-LRHIKLENNENK-PVTNSRDAQEVP------------------------HAKGVTVLRIL
	XP_017789685	HlYTH DC1	AR------------FFIIKSNNAEN--VTL--SKAKGVWSTLPQNEANLNQA---YR--ESRN---VLLVFS--VKESGKFA------------------------------------GFARL---STESRRDGAP-ISWV-LP---------------------PGLSAK-------AL-GGVFKVDWICRKELPFTA-TLHLYNPWNDGK-QVKIGRDGQEIE------------------------PRVAEELCRLF
Pieris rapae	XP_022130163	PrYTH DF	TR------------FFVIKSYSEDD--IHR--SIKYEIWCSTEHGNKRLDSA---FR--DRERE---GGS-----VYLFFS--VNGSGHFC------------------------------------GMARM---VSAVDYNSNS-SVWS-QD---------------------------------KW-KGQFRVRWIYVKDVPNGQ-LRHIKLENNENK-PVTNSRDTQEVP------------------------HAKGLQVLRIM

	XP_022113565	PrYTH DC1	TR------------FFLIKSNNAEN--ITL--SKAKGVWSTLPQNEANLNQA---YR---ESRN-----------------------------------<br>-----------------------VLLIFS--VKESGKFA---------------------------------------GFARL---GSESRRDVPA-<br>VSWV-LP-----------------------PGLSAK-------VL-DGVFKVDWICRKELPFSS-TLHLYNPWNEGK-PVKIGRDGQEIE--------------------------PKVAEELCRLF
	XP_022129346	PrYTH DC2	SR------------YFIVKADDLHS--VEM--AQASGSYPFTQNTIKKLQKA---KQ---EGSR-----------------------------------<br>------------------------VIMIFS--CAGASKIT--------------------------------------GAAAL--------------------GEA--------<br>---------------------------------AS--PLDWISNNHIPFHM-LRHITNGLAGGA-RVTGARDGTELC--------------------------STSGRGLLAAL
Frankliniella occidentalis	XP_026290617	FoYTH DF	AR------------FFVIKSYSEDD--IHR--SIKYEIWCSTEHGNKRLDEA---YR---EREAE-----------------------------------<br>---------------GGI-----VLLFFS--VNGSGHFC--------------------------------------GMAQM---ISPLDYNASS-<br>SVWS-QD-------------------------------------KW-KGQFGVRWIYVKDVPNVQ-LRHIRLENNENK-PVTNSRDTQEVP--------------------------FEKGRQVLRIM
	XP_026272952	FoYTH DC1	AR------------FFVIKSNNAEN--VTL--SKAKGVWSTLPQNETKLNQA---FK---ESRN----------------------------------<br>-------------------------VLLIFS--VKESGRFA--------------------------------------GFARL---NGESRHDVAP-<br>ISWV-LP-----------------------PGLSAK-------AL-GGVFKVDWVCRKELPFTA-TMHLYNPWNEGK-PVKIGRDGQEIE--------------------------PRVAEELCRLF
	XP_026287428	FoYTH DC2	R------------YFIIKAGNPRA--INA--SLRENVWAFTATTENKLITV---FR---EGFA---------------------------------------<br>--------------------VMLIFS--IHGSGNFQ--------------------------------------GVASL---VSDVSDVRDSGKVHV-<br>Q--------------------------DMAGP-------NL-CSPLPIKWLKYGDIPFHA-TRNIMNPYNENR-NIQTSRDGQEVE--------------------------PHVASQLCKLW
Hyposmocoma kahamanoa	XP_026323400	HkYTH DF	AR------------FFVIKSYSEDD--IHR--SIKYEIWCSTEHGNKRLDAA---FR---ERERE-----------------------------------<br>--------------GGM-----VYLFFS--VNGSGHFC--------------------------------------GMARM---MSAVDYNSNS-<br>SVWS-QD-------------------------------------KW-KGQFRVKWIYVKDVPNVQ-LRHIKLENNENK-PVTNSRDTQEVP--------------------------HAKGLQVLRIM
	XP_026316753	HkYTH DC1	TR------------FFLIKSNNAEN--ITL--SKAKGVWSTLPQNEANLNQA---YR---ESRN-----------------------------------<br>-----------------------VLLIFS--VKESGRFA---------------------------------------GFARL---ASESRRDVPP-ISWV-<br>LP-----------------------PGLSAK-------VL-DGVFKVDWICRKELPFNS-TLHLYNPWNEGK-PVKIGRDGQEIE--------------------------PKVAEELCRLF
	XP_026333402	HkYTH DC2	VR------------YFVVKTDDIHS--VEAA-AQNNGTFAFSPNTAKKLQKV---KQ---EGHR--------------------------------<br>---------------------------VVLFFS--CAASSKFV--------------------------------------GCASL-------------------LDP--<br>------------------------------------NG--TLDWAHAHQLPFHM-VRHISNSLAGGA-RIAGARDGTELC--------------------------PASGRALYAAL
Bombyx mori	XP_004924605	BmYTH DF	TR------------FFVIKSYSEDD--IHR--SIKYEIWCSTEHGNKRLDAA---YR---EREKE----------------------------------<br>---------------GGM-----VYLFFS--VNGSGHFC--------------------------------------GMARM---MSAVDYNSNS-<br>SVWS-QD-------------------------------------KW-KGQFRVKWIYVKDVPNAQ-LRHIKLENNENK-PVTNSRDTQEVP--------------------------HPKGLQVLRIM
	XP_004931496	BmYTH DC1	TR------------FFLIKSNNAEN--ITL--SKARGVWSTLPQNEANLNQA---YR---ESRN-----------------------------------<br>-----------------------VLLIFS--VKESGKFA---------------------------------------GFARL---GSESRRDVPP-ISWV-<br>LP-----------------------PGLSAK-------VL-DGVFKVDWICRKELSFNS-TLHLYKPWNEGK-PVKIGRDGQEIE--------------------------PKVAEELCRLF
	XP_012544932	BmYTH DC2	AK------------QFMIKVDELHA--LEA--AYSTGSYVFAQNIAKRLQKA---KM---EGHR--------------------------------<br>--------------------------VTVFFS--CPSANKFV--------------------------------------GCANL-------------------LDV--<br>------------------------------------SG--ALEWISTQHVPYHM-VRH----SCGGA-RGCVWREG-ELC--------------------------GAGGGALLAHS
Tribolium castaneum	XP_015835734	TcYTH DF	AR------------FFVIKSYSEDD--IHR--SIKYEIWCSTEHGNKRLDQA---YR---EREAH-----------------------------------<br>----------------GA-----VYLFFS--VNGSGHFC--------------------------------------GMAQM---VSPVDYNANS-<br>SVWS-QD-------------------------------------KW-KGQFKVRWIYVKDVPNVQ-LRHIRLENNENK-PVTNSRDTQEVP--------------------------HAKGLQVLRIM

	XP_969804	TcYTH DC1	AR------------FFVIKSNNAEN--ITL--SKAKGVWSTLPQNEANLNKA---YR--ESRN--VLLIFS---VKESGKFA-------------------------------------GFARL---HSESRHDVPA-ISWV-LP----------------------PGLSAK-------AL-GGVFKVDWICRKELPFSN-TMHLYNPWNDGK-PVKIGRDGQEIE-------------------------PRVAEELCRLF
	XP_008198757	TcYTH DC2	SV------------FLLIKVKQECS--VKI--AFDSNKWIFSPQTEKQVMAL---DNGGMN--VYLVYT---VQKLNAFY-------------------------------------GIAKY---LT--------------FDTT---------------------------------RH-KTSAAIEWIFKSCVPYKS-VSHLRNALNNNR-LIYDGLDGQEIE-------------------------MGAALQLW
Priapulus caudatus	XP_014679284	PcaYTH DFa	SR------------FFVIKSYSEDD--IHR--SIKYSIWCSTEHGNKRLDTA---FK---ERDSK--GS-----VYLFFS--VNGSGHFC-------------------------------------GLAEM---LTPIDHDTKS-SVWA-QD-----------------------------------KW-KGQFQVRWIYVKDVPNSA-LRHIRLENNENK-PVTNSRDTQEVP-------------------------ADKGKQVLKIM
	XP_014672005	PcaYTH DFb	AR------------FFIIKSYSEDD--IHR--SIKYSIWCSTEHGNRRLDQA---FK---ERHGK--GP-----VYLLFS--VNGSGHFC-------------------------------------GVAQM---LSEVDYDTQS-GVWA-QS-----------------------------------KW-KGQFQVKWIYVKDVPNSQ-LRHIRLENNENK-PVTNSRDTQEVP-------------------------FEQGKQVLKII
	XP_014667135	PcaYTH DC1	SA------------YFLIKSNNHEN--ISL--AKAKGVWSTPPQNEAKLNKA---FK--ECRN--VILVYS--VKESGKFQ-------------------------------------GYARL---SSESRHDIAP-PQWV-LP----------------------PGMTSR-------AL-GGVFKIDWVNRKELAFSK-TLHLHNSWNENK-PVKIGRDGQEIE-------------------------HNCGESLCRLF
Clonorchis sinensis	RJW65808	CsYTH DF	AR------------FFVIKSFSEDD--IHR--SIKYSVWCSTELGNKKLDSA---YV--GANNQ---YP-----IYLFFS--VNGSGHFC-------------------------------------GMAEM---TSRVDYDTRV-RVWA-QD-----------------------------------KW-QGAFSVRWIFVKDVPNTA-LRHIRIESNENK-PVTHSRDATELP-------------------------LERGRQVMEVF
	RJW63992	CsYTH DC1	AR------------YFMIKSNNYEN--VEI--AKTRNVWSTTKGNETRLNKA---FF--DCPN---VFLIFS--VRESGKFQ-------------------------------------GFAQI---IASSDPRI-K-VNWV-LP----------------------PRMDIN-------LL-SNPFRIKWISKRDLPFSK-VNHLTNPWNENK-PVKIGRDGQEIE-------------------------GVCGEALCRLL
Opisthorchis viverrini	OON14740	OvYTH DF	AR------------FFVIKSFSEDD--IHR--SIKYSVWCSTELGNKKLDSA---YV--GANNQ---YP-----IYLFFS--VNGSGHFC-------------------------------------GMAEM---TSRVDYDTRV-RVWA-QD-----------------------------------KW-QGAFSVRWIFVKDVPNTA-LRHIRIESNENK-PVTHSRDATELP-------------------------LERGRQVMEVF
	XP_009167386	OvYTH DC1	AR------------YFMIKSNNYEN--VEI--AKTRNVWSTTKGNETRLNKA---FF--DCPN---VFLIFS--VRESGKFQ-------------------------------------GFAQI---IASSDPRI-K-VNWV-LP----------------------PRMDIN-------LL-SNPFRIKWISKRDLPFSK-VNHLTNPWNENK-PVKIGRDGQEIE-------------------------GVCGEALCRLL
Schistosoma haematobium	XP_012796317	ShYTH DF	AR------------FFVIKSFSEDD--IHR--SIKYSIWCSTELGNKKLDTA---FA---EANHA--YP-----IYLFFS--VNGSGHFC-------------------------------------GMAEM---VSRVDYNARA-SVWA-QD-----------------------------------KW-QGKFSVRWIFVKDVPNTA-LRHIRIETNDNK-PVTHSRDTTELP-------------------------LERGKQVMEVL
	XP_012801596	ShYTH DC1	TK------------YFLIKSNNFEN--IEI--AKSRNVWATTKGNETRLNKA---FF--DYNN---VLLIFS--VRESGRFQ-------------------------------------GFARI---IASSDPRI-K-VDWV-LS----------------------SRMSTG-------LL-SNPFRIKWISKSDLPFTK-TGHLLNAWNEDK-PVKIGRDGQEIE-------------------------PTCGEALCRCF
Orbicella faveolata	XP_020606490	OfYTH DF	AR------------FFIIKSYSEDD--IHR--SIKYSVWTSTEHGNRRLNEA---FK---EQQGA--KAP-----IYLLFS--VNGSGHFC-------------------------------------GIAMM---MSEVDLEIET-GIWS-QD-----------------------------------KW-KGKFEVKWFYVKDVPNNA-LRHIRLENNDNK-PVTNSRDTQEVP-------------------------PEKGKQVLKII

	XP_020617190	OfYTH DC1	AR------------YFVMKSNNHEN--VAL--SKAKGVWSTPKANEKKLNAT---FK---RYKN--------------------------<br>---------------------------VILIFS--VKESGKFQ--------------------------------------GFARL---ASEAKHGGQP-<br>MPWV-LP---------------------PGMSAK-------AL-GGVFKLEWLNRRELWFSK-CLHLRNPWNDNK-EVKICRDGQEVE------------------------PSVGEALCRLF
	XP_020627768	OfYTH DC2	AR------------YFIMKCNNQRN--LDI--SMAKGIWATTLANEKKLNRA---FK---ETKL---------------------------------<br>------------------------VILVFS--VQGSGHFQ--------------------------------------GYAQM---TSPIGKDKSP------<br>-------------------------EFGSS-------SL-SGVFSVEWIKKASIPFQQ-AHHLVNPWNDHK-KVQISRDGQELE------------------------PKIGEDLCKLW
Exaiptasia pallida	XP_020892001	EpYTH DF	AR------------FFIIKSYSEDD--IHR--SIKYSVWTSTEHGNRRLNEA---FK---EQSKS-----------------------------------<br>--------------GP-----IYLIFS--VNGSGHFC--------------------------------------GIAQM---MSDVDFDIET-<br>GIWS-QD-----------------------------------KW-KGKFDVKWFYVKDVPNNA-LRHIRLENNDNK-PVTNSRDTQEIP------------------------PDKARQVVKII
	XP_020896581	EpYTH DC1	AR------------YFVMKSNNAEN--VLL--SKAKGVWSTPRTNEKKLSSA---FR---RYKN------------------------------<br>--------------------------VIVIFS--VKESGKFQ--------------------------------------GMARL---MGEATYGGPQ-<br>IPWV-LP----------------------PGMSAK-------AL-GGVFPIKWLNRQDLWFSK-CTHLRNPWNDNK-EVKICRDGQEVE------------------------PTVGEQLCRLF
	KXJ22716	EpYTH DC2	AR------------YFIIKCNNQRN--LSI--SMAKGIWATTIANEKKLNRA---FR---ECKK----------------------------------<br>----------------------IVLIFS--VQGSGHFQ--------------------------------------GFAHM---ASPIGKDKSP----------<br>---------------------EFGSS-------SL-GGVFKVDWIKKANIPFQE-AHHLVNPWNDHK-KVQISRDGQELE------------------------PTVGADLCKLW
Stylophora pistillata	XP_022806830	SpiYTH DF	AR------------FFIIKSYSEDD--IHR--SIKYSVWTSTEHGNRRLNDA---FK---EQQSS----------------------------------<br>-------------KAP-----IYLLFS--VNGSGHFC--------------------------------------GIAMM---TSEVDIEIET-<br>GIWT-QD-----------------------------------KW-KGKFDVKWFYVKDVPNNA-LRHIRLENNDNK-PVTNSRDTQEVP------------------------PEKGKQVLKII
	PFX31771	SpiYTH DC2	AR------------YFIMKCNNQRN--LDI--SMAKGIWATTLANEKKLNKA---FK---ESKH-------------------------------<br>-------------------------VMLVFS--VQGSGHFQ--------------------------------------GYAQM---TSPIGKDKSP----<br>---------------------------EFGSS-------SL-SGVFSVEWMKKASIPFQQ-AHHLVNPWNDHK-KVQISRDGQELE------------------------PKIGEELCMLW
Amphimedon queenslandica	XP_003386824	AqYTH DF	AR------------FFVIKSYAEDD--VHR--SIKYNVWCSTDHGNRRLDTA---FK---EQKSK------------------------------<br>-----------------GGG-----VYLLFS--VNGSGHFC--------------------------------------GVAQM---<br>MSEVELSTDT-GIWT-QD-----------------------------------KW-KGRFDIRWIYVKDVPNNQ-LRHIRLENNENK-PVTNSRDTQEVP------------------------VDKGKLVIKII
	XP_019850317	AqYTH DC1	TR------------YFVIKSNNYEN--VDI--AKSKNVWSTLPYNEKKLNKA---YR---DCRN-------------------------------<br>-------------------------VLLIFS--VKESGGFQ--------------------------------------GFAKL---VSESRSDVPR-<br>VHWV-LP----------------------PSMSAS-------QL-SHVFKLDWIHKGTLAFNL-CQDLKNPWNENK-PVKIGRDGQEIE------------------------PSVGEKLCKLW
Selaginella moellendorffii	XP_024531733	SmYTH DC1B	TR------------YFIIKSLNHQN--LSK--SVERGVWATPAVNEDILNEA---FQ---TSER-----------------------------------<br>---------------------VVLVFS--VNMSGYFQ--------------------------------------GYAEM---TSRPGRRKD--<br>NLWN-DA-----------------------NDGSS-------PW-GGVFSVDWLKLHDLPFQE-TSHLKNPLDDNK-PVKISKDCQELP------------------------REVGESLCALI
	XP_024516925	SmYTH DC1A	SR------------YFIVKSSNREN--LEL--SVRRGVWATHRNNEAKLNEA---LE---SCEH---------------------------------<br>-----------------------VILIFS--<br>VNETRHFQVSSLSTFFPSLFHDPFVSLRNQHKLCCVFLLRLCCSQGCARM---MSKAGGAAHGTAGWK-QS-----------------------HGMGS--------Y-<br>GRNFRLKWLKLCELSFHK-TRHLHNPYNENL-PVKISRDCQEVE------------------------PSVGQELAELL
	XP_002961786	SmYTH DF1	AK------------FFIIKSYSEDD--VHK--SIKYGVWASTPNGNKRLDAA---YK---EAAGE-----F-------------------------<br>-------------------P-----IFLFFS--VNGSGQFC--------------------------------------GVAEM---SGPMDFLRSV-<br>DFWQ-QD-----------------------------------KW-TGRFSVKWHFIKDITNGH-FRHIILENNDNK-PVTNSRDTQEVQ------------------------LDQGLEMLRIF

	Phvul.002G152600	PvYTH DC1B	IK------------YFIIKSLNREN--IDL--SIKKGIWATQIMNERILEEA---FH--NSGC---VILIFS--VNMSGSFQ--------------------------------------GYAQM---MSSIGRGRD--NVWS-EG-----------------------IGKSN-------PW-GRSFKVQWLCFNDLPFYK-TLHLKNPLNDYK-PVKISRDCQELS-------------------------PDIGLALCELL
	Phvul.006G130200	PvYTH DC1A	SR------------YFIVKSCNREN--LEL--SVQQGVWATQRSNESKLNEA---FD--SVEN--VILIFS--VNRTRHFQ---GCAKM---TSRIGGSVAG-GNWK-YA-----------------------HGTAH--------Y-GRNFSVKWLKLCELSFHK-TRHLRNPYNENL-PVKISRDCQELE-------------------------PSIGEQLASLL
	Phvul.006G121600	PvYTH DF1C	AK------------FFVIKSYSEDD--VHK--SIKYNVWSSTPHGNKKLENA---YE--DAKKI-AAE--KSGVCP-----IFLFFS---VNASGQFC---------------------------------------GVAEM---IGTVDFNKNM-DFWQ-QD-----------------------------------KW-SGSFPVKWHFIKDVPNPN-FRHIILENNENK-PVTNSRDTQEIV-------------------------YLKGLEMLKIF
	Phvul.003G119300	PvYTH DF2C	AK------------YFVIKSYSEDD--VHK--SIKYNVWSSTPHGNKKLQNA---YE--DAERI-AAG---KSGGCP-----VFLFFS--VNASGQFC---------------------------------------GVAEM---AGPVDFNKDM-DFWQ-QD-----------------------------------KW-SGSFPVKWHIIKDVPNAN-FRHIILENNENK-PVTNSRDTQEIM-------------------------YGKGLEMLKMF
	Phvul.004G132700	PvYTH DF3C	AL------------FFVIKSYNEED--IHK--SIKYNVWASTPNGNKRLDGA---YQ--DAQKR-MED---KGCKCP-----VFLFFS---VKWHIIKDVPNPQ-LRHIILENNDHK-PVTN------MN-------------------------FPQGVEILNIF
Phaseolus vulgaris	Phvul.005G045600	PvYTH DF4C	AL------------FFVIKSYSEDD--IHK--SIKYNVWASTPNGNKRLDGA---FQ--DAQKR-MEE---KGCKCP-----VFLFFS--VNASGQFC---------------------------------------GVAEM---TGRVDFNKSM-DFWQ-QD-----------------------------------KW-NGYFPVKWHIIKDVPNPQ-LRHIILENNDHK-PVTNSRDTQEVN-------------------------FPQGVEILNIF
	Phvul.010G165400	PvYTH DF1B	AK------------FFVIKSYSEDN--VHK--SIKYGVWASTPNGNRKLDAA---YR--QAMEK---QEACP-----IFLFFS---VNASAQFC---------------------------------------GVAEM---VGPVNFDKSV-DFWQ-QD-----------------------------------KW-SGQFPVKWHIIKDVPNSQ-FRHIVLENNDNK-PVTNSRDTQEVK-------------------------LPQGIEMLTIF
	Phvul.006G218800	PvYTH DF5C	AK------------FYVIKSFNEDD--VHK--GVKYNVWTSTPNGNKKLNIA---FL--DAEAK-LRQ---TGTKCP-----VFLFFS--VNASRQFV---------------------------------------GVAEM---VGPVDFKKDM-SFWK-LD-----------------------------------KY-NGFFSIKWHIIKDVPNNQ-FVHIILPSNENK-PVTFTRDTQEIG-------------------------LKQGLEMLNIF
	Phvul.002G247000	PvYTH DF1A	AK------------FFVIKSYSEDD--IHK--SIKYNVWASTQNGNKKLDAA---YQ--EAQQK-P---GGCP-----VFLFFS---VNTSGQFV---------------------------------------GLAEM---VGSVDFNKSV-EYWQ-QD-----------------------------------KW-NGCFPLKWHIVKDVPNNL-LRHITLDNNENK-PVTNSRDTQEVM-------------------------LEPGLKLIKIF
	Phvul.004G080300	PvYTH DF2A	AK------------FFVIKSYSEDD--IHK--SIKYNVWASTLNGNKKLDAA---YH--EAKEK-P--GDCP-----VFLLFS---VNTSGQFV---------------------------------------GLAEM---VSPVDFGRTL-EYWQ-QD-----------------------------------RW-TGCFSVKWHIIKDIPNSV-LRHITLENNENK-PVTNSRDTQEVN-------------------------FEKGIQVLKIF
	Phvul.001G110200	PvYTH DF3A	AK------------FFVIKSYSEDD--IHK--SIKYSVWASTPNGNKKLDAA---YQ--EAKEK-T--GGCP-----IFLLFS---VNTSGQFV---------------------------------------GLAEM---LGPVDFGKSV-DYWQ-QD-----------------------------------RW-TGCFSVKWHVIKDIPNSV-MRHITLENNENK-PVTNSRDTQEVK-------------------------FEKGVQIVKIF

	XP_006352991	StYTH DC1Ba	IR------------YFVIKSLNHEN--IQL--SVNRGIWATQAMNEAILDEA---FH--NSSK---VILIFS---VNMSGYFQ--------------------------------------GYAQM---ISSVGLRRD--QVWS-QG------------------------NGGRN-------PW-GRSFEVNWLRLYDLPFQR-TLHLKNPWNQYK-PVKISRDCQELP--------------------------PDIGEALCELL
	XP_006358382	StYTH DC1Bb	IR------------YFVIKSLNHEN--IQL--SVNRGIWATQAMNEAILDEA---FH--NSSK---VILIFS---VNMSGYFQ--------------------------------------GYAQM---ISSVGLRRD--QVWS-QG------------------------NGGRN-------PW-GRSFEVNWLRLYDLPFQR-TLHLQNPWNQYK-PVKISRDCQELP--------------------------PDIGEALCELL
	XP_006343481	StYTH DC1Aa	SR-------------YFIVKSCNREN--LEL--SVQQGVWATQRSNEAKLNEA---FD--SVEN--VILIFS---VNRTRHFQ--------------------------------------GCGKM---TSRIGGAANG-GNWK-HE------------------------HGTAH--------Y-GRNFSVKWLKLCELSFQK-THHLRNPYNENL-PVKISRDCQELE--------------------------PSVGEQLASLL
	XP_006359103	StYTH DC1Ab	SR-------------YFIVKSCNREN--LEL--SVQQGVWATQRSNEAKLNEA---FD--SVEN--VILVFS---INRTRHFQ--------------------------------------GLAKM---TSRIGGAAKG-GNWK-HE------------------------HGTAH--------Y-GRNFSLKWLKLCELSFQK-TRHLRNPYNENL-PVKISRDCQELE--------------------------ISVGEQLASLL
Solanum tuberosum	PGSC0003DMG400004607	StYTH DF1C	AK-------------FFVIKSYSEDD--VHK--SIKYNVWSSTPNGNKKLNSA---YE--DAQRI-TAG---NPRGCP-----IFLFFS---VNASGQFC--------------------------------------GVAEM---TGPVDFYNDM-DFWQ-QD------------------------------------KW-SGSFPVKWHFIKDVPNPN-FRHIILENNENK-PVTNSRDTQEIR--------------------------YKKGIEMLKVF
	XP_006338126	StYTH DF1B	AK-------------FFVIKSFSEDN--VHK--SIKYSVWASTPQGNRKLDAA---YA--EAKEM--NANCP-----VFLFFS---VNASGQFC--------------------------------------GVAEM---VGPIDFENNA-EYWQ-QD------------------------------------RW-SGKFPVKWHVIKDVPNSQ-FRHILLEHNDNK-PVTHSRDSQQVK--------------------------LPEGLEMLKIF
	PGSC0003DMG400024891	StYTH DF2C	AK-------------FYIIKSYSEDD--IHK--CVKYDVWSSTPNGNKKLDTA---FV--EAEAK-SSG---TGSSCP-----VFLFFS---VNGSGQFL--------------------------------------GVAEM---VGQVDFNRNM-DFWQ-LD------------------------------------KW-SGFFPLKWHIVKDVPNTQ-FRHIILENNDNR-PVTYSRDTQEIG--------------------------LKEGLEMLNIL
	PGSC0003DMG400024008	StYTH DF1A	AK-------------FFIIKSYSEDD--VHK--SIKYNVWASTPNGNKKLDAA---YQ--EAQQN-S---GGCP-----VFLFFS---VNTSGQFV--------------------------------------GVAEM---VGPVDFNKNV-EYWQ-QD------------------------------------KW-VGCFPVKWHIVKDVPNSL-LKHITLENNENK-PVTNSRDTQEVK--------------------------VEQGLQVLKIF
	Brara.B02704	BrYTH DC1Ba	TR-------------YFIIKSLNYDN--IQL--SVERGIWATQVMNEPILEGA---FH--NSGR---VVLIFS---VNMSGFFQ--------------------------------------GYAEM---LSPVGWRRD--HIWS-QG----------------------GGKNN-------PW-GRSFKVKWLRLSELPFQK-TLHLKNSLNDYK-PVKISRDCQELP--------------------------GDIGEALCELL
Brassica rapa	RID51204	BrYTH DC1Bb	TR-------------YFIIKSLNYDN--IQL--SVERGIWATQVMNEPILEGA---FH--NSGR---VVLIFS---VNMSGFFQ--------------------------------------GYAEM---LSPVGWRRD--HIWS-QG----------------------GGKNN-------PW-GRSFKVKWLRLSELPFQK-TLHLKNPLNDYK-PVKISRDCQELP--------------------------GDIGEALCELL
	Brara.H01885	BrYTH DC1Aa	NR-------------YFVVKSNNSEN--FEL--SVQQGVWATQRSNEAKLNEA---FD--TVNN--VILIFS---VNRTRHFQ--------------------------------------GCAKM---TSRIGGYIGG-GNWK-NE------------------------HGTQQ--------Y-GGNFSVKWLKLCELSFHK-TRNLRNPYNENL-PVKISRDCQELE--------------------------PSVGEELASLL

	Brara.G00734	BrYTH DC1Ab	NR------------YFVVKSCNLEN--FEL--SVQRGVWATQRSNEAKLNEA---FD--SVAN---VILIFS--VNRTRHFQ----------------------------------GCAKM---TSRIG----G-ENWK-HE-----------------------HGTAQ--------F-GRNFSVKWLKLCELSFHK-TRNLRNPYNENL-PVK
	Brara.F00450	BrYTH DF1C	AK------------FFVIKSYSEDD--VHK--SIKYSVWSSTLHGNKKLQSA---YE---DAQRI-ATE--KSCECP-----IFLFFS--VNASGLFC----------------------------------GMAEM---TGPVSFEKDM-DFWQ-QD-----------------------------------KW-SGSFPVKWHIIKDVPNSY-FRHIILHNNENK-PVTNSRDTQEIM-----------------------MKQGLEVLKIF
	Brara.G03650	BrYTH DF2C	AM------------FFVIKSYSEDD--IHK--SIKYSVWSSTLNGNKKLDNA---FQ---ESQKK-VAE--KGGTCP-----VFLFFS--VNASGQFC----------------------------------GVAEM---TGRVDYETSM-DFWQ-QD-----------------------------------KW-TGYFPVKWHIIKDVPNPQ-LRHVILENNENK-PVTNSRDTQEVR------------------------LPQGNEVLNIF
	XP_009124755	BrYTH DF3C	AM------------FFVIKSYSEDD--IHK--SIKYNVWSSTLNGNKKLDSA---YQ---ESQKK-VAE--NGGTCP-----VFLFFS--VNASGQFC----------------------------------GVAEM---TGRVDYEKSM-EFWQ-QD-----------------------------------KW-TGYFPVKWHIIKDVPNPQ-LRHVILENNENK-PVTNSRDTQEVR------------------------LPQGNEVLNIF
	Brara.B02362	BrYTH DF4C	AM------------FFVIKSYSEDD--IHK--SIKYNVWSSTLNGNKKLDSA---YQ---ESQKK-VAE--KGGTCP-----VFLFFS--VNASGQFC----------------------------------GVAEM---TGRVDYEKSM-EFWQ-QD-----------------------------------KW-TGYFPVKWHIIKDVPNPQ-LRHVILENNENK-PVTNSRDTQEVR------------------------LPQGNEVLDIF
	Brara.G02088	BrYTH DF5C	AM------------FFVIKSYSEDD--IHK--SIKYNVWSSTLNGNKKLDSA---YQ---ESQKK-VAE--KGGTCP-----VFLFFS--VNASGQFC----------------------------------GVAEM---TGRVDYEKSM-EFWQ-QD-----------------------------------KW-TGYFPVKWHIIKDVPNPQ-LRHVILENNENK-PVTNSRDTQEVR------------------------LPQGNEVLNIF
	Brara.E02805	BrYTH DF1B	AK------------LFIIKSYSEDN--VHK--SIKYNVWASTANGNKKLDAA---YR---EAKEE---KEPCP-----VFLLFS--VNASSQFC----------------------------------GVAEM---IGPVDFEKSV-DYWQ-QD-----------------------------------KW-SGQFPVKWHIIKDVPNSQ-FRHIILENNDNK-PVTNSRDTQEVK------------------------LEQGIEMLKIF
	Brara.A03292	BrYTH DF2B	AK------------LFIIKSYSEDN--VHK--SIKYNVWASTPNGNKKLDAA---YR---EAKEE--KEACP-----VFLLFS--VNASSQFC----------------------------------GVAEM---IGPVDFEKSV-DYWQ-QD-----------------------------------KW-NGQFPVKWHIIKDVPNSQ-FRHIILENNDNK-PVTNSRDTQEVK------------------------LEQGIEMLKIF
	Brara.H02025	BrYTH DF3B	AK------------FFVIKSYSEDN--VYK--SMKYCVWASTKNGNKKLDAA---YR---EAKTK--EVACP-----VFLLFS--VNASAQFC----------------------------------GVAEM---VGPVDFETSV-EYWQ-QD-----------------------------------RW-SGHFPVKWVIVKDVPNSL-FRHIIIEDNDNK-PVTNSRDTQEVG------------------------VEQGIEMINIF
	Brara.B01161	BrYTH DF4B	AK------------FFIVKSFSEDN--VHR--SIKYNVWASTPHGNKKLDTA---YR---DAEKM---GGKCP-----IFLFFS--VNASGQFC----------------------------------GVSEM---VGPVDFEKDA-GYWQ-QD-----------------------------------RW-SGQFPVKWHILKDVPNNR-FSHILLQNNDNK-PVTHSRDSQEVK------------------------LRQGIEMLRIF
	Brara.C01152	BrYTH DF5B	AR------------FFIVKSFSEDN--VHR--SIKYNVWASTPHGNKKLDTA---YR---DAEKM---GGKCP-----IFLFFS--VNASGQFC----------------------------------GVAEM---

			VGPVDFEKDA-AYWQ-QG---------------------------------KW-NGQFPVKWHIVKDVPNNR-FSHILLQNNDSK-PVTHSRDSQEVK------------------------LRQGIEMLRIF
	Brara.F00638	BrYTH DF6C	AT------------FFVIKSYSEDD--VHK--SIKYSVWSSTVNGNKKLDAA---YR--DAEAK-TLV--DGKKRP-----IFLFFS--VNASRQFV------------------------------------GLAEM---VGYVDMNKDL-DFWQ-VD---------------------------------KW-CGFFPVEWHVVKDVPNWE-LCHIVLHNNEGK-AVTHTRDTQEIK------------------------LKEGLQMLSIF
	Brara.C03469	BrYTH DF1A	AM------------FFVIKSYSEDD--VHK--SIKYNVWASTPNGNKKLAAA---YQ--EAQQK-P---GGCP-----IFLFFS--VNASGQFV------------------------------------GLAEM---TGPVDFDTNV-DCWQ-QD---------------------------------KW-TGSFPLKWHIVKDVPNSL-LKHITLENNENK-PVTNSRDTQEVK------------------------LEQGLKIVKIF
	Brara.A03258	BrYTH DF2A	AM------------FFVIKSYSEDD--VHK--SIKYNVWASTPNGNKKLAAA---YQ--EAQQK-P---GGCP-----IFLFFS--VNASGQFV------------------------------------GLAEM---TGPVDFDTNV-EYWQ-QD---------------------------------KW-TGSFPLKWHIVKDVPNSL-LKHITLENNENK-PVTNSRDTQEVK------------------------LEQGLKIVKIF
	Brara.E02771	BrYTH DF3A	AM------------FFVIKSYSEDD--VHK--SIKYNVWASTPNGNKKLAAA---YQ--EAQQK-P---GGCP-----IFLFFS--VNASGQFV------------------------------------GLAEM---TGPVDFNTNV-EYWQ-QD---------------------------------KW-TGSFPLKWHIVKDVPNSL-LKHITLENNENK-PVTNSRDTQEVK------------------------LEQGLKIVKIF
	Brara.B02570	BrYTH DF4A	AK------------LFVIKSYSEDD--IHK--SIKYNVWSSTPNGNKKLDAA---YN--EAKEK-SN--GSCP-----VFLMFS--VNTSGQFV------------------------------------GLAEM---VGPVDFNQTV-EYWQ-QD---------------------------------KW-IGCFPVKWHIVKDIPNSS-LRHITLENNENK-PVTNSRDTQEVT------------------------LEQCMKVIKIF
	Brara.G02587	BrYTH DF5A	AK------------LFVIKSYSEDD--VHK--SIKYNVWSSTPNGNKKLNAA---YN--EAKDK---SCP-----VFLIFS--VNTSGQFV------------------------------------GLAEM---VGPVDFNQTV-EYWQ-QD---------------------------------KW-VGCFPVKWHIVKDIPNSS-LRHITLENNENK-PVTNSRDTQEVK------------------------IEQGVKVIKIF
	Brara.A03796	BrYTH DF6A	AK------------FFVIKSYSEDD--VHN--SIKYGMWSSTPTGNKKLNAA---Y---QES-S---QDCP-----VYLLFS--VNASGQFV------------------------------------GVAEM---TGPVDFNKTM-EYWQ-QD---------------------------------KW-IGCFPVKWRIIKDVPNSL-LRHITLVNNENK-PVTNSRDTQEVK------------------------LEDGIKIIKIF
	Brara.E03479	BrYTH DF7A	EK------------FFVIKSYSEDD--VHN--SIKHGVWSSTPTGNKKLNAA---YY--EAQES-C---CP-----VYLLFS--VNASGQFV------------------------------------GVAEM---TGPVDFNKTV-EYWQ-QD---------------------------------KW-IGCFPVKWHIIKDVPNSF-LRHVTIANNENK-PVTNSRDTREVS------------------------VEDGTKIIKIF
Boechera stricta	Bostr.25463s0115	BsYTH DC1B	TR------------YFIIKSLNYEN--IQV--SVEKGIWATQVMNEPILEGA---FH--KSGR--VILIFS--VNMSGFFQ------------------------------------GYAEM---LSPVGWRRD--HIWS-QG----------------------GGKNN-------PW-GRSFRVKWLRLSELPFQK-TLHLKNPLNDYK-PVKISRDCQELP------------------------EDIGEALCELL
	Bostr.15697s0342	BsYTH DC1A	NR------------YFVVKSCNREN--FEL--SVQQGVWATQRSNEAKLNEA---FD--SVEN---VILIFS--VNRTRHFQ------------------------------------GCAKM---TSRIGGYIGG-GNWK-HE----------------------HGTAQ--------Y-GRNFSVKWLKLCELSFHK-TRNLRNPYNENL-PVKISRDCQELE------------------------PSIGEQLASLL
	Bostr.19424s0931	BsYTH DF1C	AK------------FFVIK--SEDD--VHK--SIKYGVWSSTLHGNKKLQSV---YE--DAQRI-ATE--KSCKCP-----IFLFFS--VNASGLIC------------------------------------GVAEM---

			TGPVYLDRDM-DFWQ-QD----------------------------------KW-SGSFPVKWYIIKDVPNSY-FRHIILHNNENK-PVTNSRDTQEIM------------------------- LKQGLEVLKLF
	Bostr.15654s0023	BsYTH DF2C	AK------------FFVIKSYSEDD--VHK--SIKYNVWSSTLHGNKKLQSA---YE---DAQKI-ATK-------------------------------------KSCECP-----IFLFFS--VNASGLFC-------------------------------------GMAEM---TGPVSFDKDM-DFWQ-QD----------------------------------KW-SGSFPVKWHIIKDVPNSY-FRHIILQNNENK-PVTNSRDTQEIM-------------------------LKQGLEVLKIF
	Bostr.20129s0365	BsYTH DF3C	AI------------FFVIKSYSEDD--IHK--SIKYNVWSSTLNGNKKLDSA---YQ---ESQKK-IAE---------------------------------------KSVKCP-----VFLFFS--VNASGQFC-------------------------------------GVAEM---VGRVDYKKSM-EFWQ-QD----------------------------------KW-TGYFPVKWHIIKDVPNPQ-LRHIILENNENK-PVTNSRDTQEVR-------------------------LPQGNEVLNIF
	Bostr.3640s0080	BsYTH DF1B	AR------------LFIIKSYSEDN--VHK--SIKYNVWASTPNGNKKLDAA---YR---EAKDE--KEPCP-----LFLLFS--VNASSQFC-------------------------------------GVAEM---VGPVDFEKSV-DYWQ-QD----------------------------------KW-SGQFPVKWHIIKDVPNSQ-FRHIILENNDNK-PVTNSRDTQEVK-------------------------LEQGIEMLKIF
	Bostr.15697s0116	BsYTH DF2B	TK------------FFVIKSYSEDN--VHK--SIKYCVWASTKNGNKKLDAA---YR---EAKTK---DVACP-----VFLLFS--VNASAQFC-------------------------------------GVAEM---VGPVDFNTSV-EYWQ-QD----------------------------------RW-SGHFPVRWLIVKDVPNSL-FRHIIIENNDNK-PVTNSRDTQEVG-------------------------LEQGIEMLNIF
	Bostr.26833s0433	BsYTH DF3B	AK------------FFIVKSFSEDN--VHR--SIKYNVWASTPHGNKKLDTA---YR---DAEKM---GGKCP-----IFLFFS--VNASGQFC-------------------------------------GVSEM---VGPVDFDKDA-GYWQ-QD----------------------------------RW-SGQFPVKWHIVKDVPNNR-FCHILLQNNDNK-PVTHSRDSQEVK------------------------- LRQGIEMLRIF
	Bostr.25494s0041	BsYTH DF4C	AK------------FFVIKSYSEDD--VHK--SIKYSVWSSTPNGNKKLDAA---FR---DAETK-TLE-------------------------------------DGKKRP-----IFLFFS--VNASRQFV-------------------------------------GLAEM---VGYVDFKKDL-DFWQ-VD----------------------------------KW-SGFFPVEWHVVKDIPNWE-LRHIILDNNEDK-PVTHTRDTHEIK------------------------- LKEGLQMLSIF
	Bostr.3640s0139	BsYTH DF1A	AM------------FFIIKSYSEDD--VHK--SIKYNVWASTPNGNKKLAAA---YQ---EAQQK-P--GGCP-----IFLFFS--VNASGQFV-------------------------------------GLAEM---TGPVDFNTNV-EYWQ-QD----------------------------------KW-TGSFPLKWHIVKDVPNSL-LKHITLENNENK-PVTNSRDTQEVK------------------------- LEQGLKIVKIF
	Bostr.26833s0133	BsYTH DF2A	AK------------FFVIKSYSEDD--IHK--SIKYNVWSSTPNGNKKLDAS---FN---EAKQK-S---DSCP-----VFLLFS--VNTSGQFV-------------------------------------GLAEM---VGPVDFNKTV-EYWQ-QD----------------------------------KW-IGCFPVKWHFVKDIPNSS-LRHITLENNENK-PVTNSRDTQEVK-------------------------LEQGIKVVKIF
	Bostr.2570s0314	BsYTH DF3A	AK------------FFVIKSYSEDD--VHN--SIKYGVWSSTPTGNKKLNAA---YY---EAKEN-S---QECP-----VYLLFS--VNASGQFV-------------------------------------GLAEM---VGPVDFNKTM-EYWQ-QD----------------------------------KW-IGCFPVKWHIIKDIPNSL-LRHITLANNENK-PVTNSRDTQEVN-------------------------LEHGTKIIKIF
Linum usitatissimum	Lus10006706.g	LuYTH DC1Ba	TR------------YFIIKSLNHHN--LQL--SVDEGIWATQVMNEPILEEA---FN---NSGK--VILIFS--VNMSGFFQ-------------------------------------GYAQM---VSSIGLRHD--HIWS-QG----------------------SGKSG-------SW-GRSFKVKWLQLNDLPFQR-TLHLKNPLNEYK-PVKISRDCQELP-------------------------EDIGETLCRLI

	Lus10007048.g	LuYTH DC1Bb	TR------------YFIIKSLNHHN--LQL--SVDEGIWATQVMNEPILEEA---FN--NSGK---VILIFS--VNMSGFFQ---GYAQM---VSSIGLRHD--HIWS-QG-----------------------SGKVG-------SW-GRSFKVKWLQLNDLPFQR-TLHLKNPLNEYK-PVKISRDCQELP----------------------------EDIGEALCRLI
	Lus10023254.g	LuYTH DC1A	SR------------YFIVKSCNREN--LEL--SVQQGVWATQRSNEAKLNEA---FD--SAEN--VILVFS--VNRTRHFQ---GCAKM---TSKIGATIGG-GNWK-YQ-----------------------HGTAH--------Y-GRNFSVKWLKLCELSFHK-TRHLRNPYNENL-PVKISRDCQELE----------------------------PSVGEQLASLL
	Lus10017111.g	LuYTH DF1C	AK------------FFVIKSYSEDD--VHK--SIKYNVWSSTLHGNKKLQSA---YE--DAQKI-AAE---KVTSCP-----VFLFFS---VNASGQFC---GVAEM---IGPVDLKKDM-DFWQ-QD---------------------------------------KW-SGSFPVKWHMIKDVQNTS-FRHIILENNEHK-PVTNSRDTQEVL----------------------------LKQGLQMLKIF
	Lus10018343.g	LuYTH DF2C	AK------------FFVIKSYSEDD--VHK--SIKYNVWSSTLHGNKKLQSA---YE--DAQKI-AAE---KATSCP-----VFLFFS---VNASGQFC---GVAEM---IGPVDLKKDM-DFWQ-QD---------------------------------------KW-SGSFPVKWHMIKDVQNTS-FRHIILENNEHK-PVTNSRDTQEVL----------------------------LKQGLQMLKIF
	Lus10034792.g	LuYTH DF1B	AK------------FFIIKSYSEDN--VHK--SIKYGIWASTPNGNKKLDAA---YA--DAKTK--EDPCP-----VFLLFS---VNASAQFC---GVAEM---TGPVNFEKSV-DYWQ-QD---------------------------------------KW-SGQFPVKWHMIKDVPNSQ-FRHIVLENNDNK-PVTNSRDTQEVK----------------------------LEQGIEMLSIF
	Lus10033335.g	LuYTH DF2B	AK------------FFIIKSYSEDN--VHK--SIKYGIWASTPNGNKKLDAA---YA--DAKTK--EDPCP-----VFLLFS---VNASAQFC---GVAEM---TGPVNFEKSV-DYWQ-QD---------------------------------------KW-SGQFPVKWHMIKDVPNSQ-FRHIVLENNDNK-PVTNSRDTQEVK----------------------------LEQGLEMLSIF
	Lus10037028.g	LuYTH DF3B	AK------------FFVIKSYSEDN--VHK--SIKYGVWASTQNGNKKLDAA---YS--EAQEK--QRAPP-----VFLLFS---VNASAQFC---GVAEM---VGPVDFEKSV-NYWQ-QD---------------------------------------KW-TGQFPVKWHIIKDVPNSQ-FRHIILENNDNK-PVTNSRDTQEVQ----------------------------LDHGIEMLNIF
	Lus10015778.g	LuYTH DF4B	AK------------FFVIKSYSEDN--VHK--SIKYGVWASTQNGNKKLDAA---YS--EAQET--QRAPP-----VFLLFS---VNASAQFC---GVAEM---VGHVDFEKSV-NYWQ-QD---------------------------------------KW-TGQFPVKWHIIKDVPNSQ-FRHIILGNNDNK-PVTNSRDTQEVQ----------------------------LDHGIEMLNIF
	Lus10027582.g	LuYTH DF5B	AK------------FFIIKSFSEDN--VHK--SVKYGVWASTPHGNKKLDAA---YN--QAKEI--DPKCP-----IFLLFS---VNASGQFC---GVAEM---LGPVDFGKDA-DYWQ-QD---------------------------------------RW-SGQFPVRWHIVKDVPNPR-FRHILLENNENK-PVTHSRDCQEVK----------------------------LKHGIEILKIF
	Lus10037365.g	LuYTH DF3C	AK------------FYVIKSYNEDD--IHK--SIKYNVWASTPNGNRKLDEA---YR--SAQEK-LSE---SGVNSP-----IFLFFS---VNGSGQFV---GIAEM---VGQVDFNKDM-DFWQ-LN---------------------------------------KW-NGFFPVKWHVVKDIPNNQ-LRHIILENNENK-PVTFSRDTQEIG----------------------------MKQGLEMLSIF
	Lus10035790.g	LuYTH DF4C	AK------------FYVIKSYNEDD--IHK--SIKYNVWASTPNGNRKLDES---YR--SAQEK-LSE---TGVKSP-----IFLFFS---VNGSGQFV---GIAEM---

			VGQVDFNKDM-DFWQ-LN----------------------------------KW-NGFFPVKWHIVKDIPNNQ-LRHIILENNENK-PVTFSRDTQEIG-------------------------MKQGLEMLSIF
	Lus10037364.g	LuYTHDF5C	AK------------FYVIKSYSEDD--IHK--SIKYNVWSSTPNGNKKLDAA---FR--DSEEK-SSE--SGTKCP-----VFLFFS---VNGSGQFV-----------------------------------GLAEM---TGLVDFNKDM-DFWQ-VD----------------------------------KW-SGFFPLKWHIVEDIPNTQ-LRHIVLENNDNR-PVTFSRDTQEIG-------------------------LKQGLEMLTIF
	Lus10002624.g	LuYTHDF1A	AK------------FFVIKSYSEDD--VHK--SIKYGVWTSTPNGNKKLDAA---YR--EASAK-A---ARCP-----VFLLFS---VNTSGQFV-----------------------------------GLAEM---TGPVDFDKTV-EYWQ-QD----------------------------------KW-TGCFAVKWHIIKDVPNST-LRHITLENNENK-PVTNSRDTQEIH-------------------------IEKGIEVLKIF
	Lus10020269.g	LuYTHDF2A	AK------------FFVIKSYSEDD--VHK--SIKYGVWTSTSNGNKKLDAA---YR--ESSAK-A---AQCP-----VFLLFS---VNTSGQFV-----------------------------------GLAEM---VGPVDFDKTV-EYWQ-QD----------------------------------KW-TGCFAVKWHIIKDVPNST-LRHITLENNENK-PVTNSRDTQEVH-------------------------IEKGIEVLKIF
Salix purpurea	SapurV1A.0260s0290	SpYTHDC1B	TR------------YFIIKSLNHHN--IQL--SIENGIWATQVRNEPILEEA---FR--NSGR--VILIYS---VNMSGFFQ-----------------------------------GYAQM---LSSVGWRHD--NLWS-EG-----------------------SGKSN-------PW-GRSFKVKWLQLNDLPFQK-TLHLKNPLNDYK-PVKISRDCQELP-------------------------EDIGEALCELI
	SapurV1A.0030s0120	SpYTHDC1A	SR------------YFIVKSCNREN--LEL--SVQQGVWATQRSNEIKLNEA---LD--SSDN---VILIFS---VNRTRHFQ-----------------------------------GCAKM---TSKIGASVGG-GNWK-YA-----------------------HGTAH--------Y-GRNFSVKWLKLCELSFHK-TRHLRNPFNENL-PVKISRDCQELE-------------------------PSIGEQLASLL
	SapurV1A.0114s0500	SpYTHDF1C	AK------------FFVIKSYSEDD--VHK--SIKYNVWSSTPHGNKKLHSA---YE--DARKL-DLG--RPRGCP-----IFLFFS---VNASGQFC-----------------------------------GVAEM---VGPVDFNRDM-DFWQ-QD----------------------------------KW-SGSFFVKWHIIKDIPNSS-FRHIILENNENK-PVTNSRDTQEIM-------------------------YRQGLEMLKMF
	SapurV1A.0072s0540	SpYTHDF2C	AK------------FFVIKSYSEDD--VHK--SIKYNVWSSTPHGNKKLQTA---FE--DAQKL-AAG--RPRGCP-----IFLFFS---VNASGQFC-----------------------------------GVAEM---IGPVDLHRDM-DFWQ-QD----------------------------------KW-SGSFLVKWHIIKDIPNSS-FRHIILVNNESK-PVTNSRDTQEIM-------------------------YKQGLEMLKIF
	SapurV1A.0149s0560	SpYTHDF3C	AF------------FFVIKSYSEDD--VHK--SIKYNVWASTPNGNKRLDSA---YQ--DAQQK-IAE---KGNSCP-----VFLFFS---VNASGQFC-----------------------------------GVAEM---IGRVDFNKTM-DFWQ-QD----------------------------------KW-NGCFPVKWHIIKDVPNPQ-LRHIILENNENK-PVTNSRDTQEVK-------------------------LPQGIDILNIF
	SapurV1A.0164s0020	SpYTHDF4C	AF------------FFVIKSYSEDD--VHK--SIKYNVWASTPNGNKRLDIA---YQ--DAQQK-IAE---KGNSCP-----VFLFFS---VNASGQFC-----------------------------------GVAEM---IGRVDFNKTM-DFWQ-QD----------------------------------KW-NGCFPVKWHIIKDVPNPQ-LRHIILENNENK-PVTNSRDTQEVK-------------------------LPQGIDILNIF
	SapurV1A.0093s0340	SpYTHDF5C	AF------------FFVIKSYSEDD--IHK--SIKYNVWASTPNGNKRLDNA---YI--DSQLK-IAE--KGCSCP-----VFLFFS---VNASGQFC-----------------------------------GVAEM---IGRVDFNKRM-DFWQ-QD----------------------------------KW-NGCFPVKWHIIKDVPNLH-LRHIILENNENK-PVTNSRDTQEVK-------------------------FPQGIEILNIF
	SapurV1A.2058s0020	SpYTHDF1B	AK------------FFIIKSYSEDN--VHK--SIKYGVWASTPNGNRKLDTA---YS--EAKDK---QDPCP-----VFLLFS---VNASAQFC-----------------------------------GVAEM---VGPVDFDKSV-DYWQ-QD----------------------------------KW-SGQFPVKWHIIKDVPNSR-FRHIVLENNDNK-PVTNSRDTQEVK-------------------------LKHGVEMLNIF

	SapurV1A.1769s0060	SpYTHDF2B	AK------------FFIIKSYSEDN--VHK--SIKYGVWASTPNGNRKLDTA---YS---EAKDK---QDPCP-----VFLLFS--VNASAQFC-------------------------------------GVAEM---VGPVDFDKSV-DYWQ-QD-----------------------------------KW-SGQFPVKWHIIKDVPNSR-FRHIVLENNDNK-PVTNSRDTQEVK-------------------------LKHGVEMLNIF
	SapurV1A.1485s0090	SpYTHDF3B	AK------------FFIIKSYSEDN--VHK--SIKYGVWASTPNGNRKLDTT---YR---EAKEK---QDPCP-----VFLLFS--VNASAQFC-------------------------------------GVAEM---TGPVDFDKSV-DYWQ-QD-----------------------------------KW-NGQFPVKWHLIKDVPNSQ-FRHIVLENNDNK-PVTNSRDTQEVK-------------------------LEQGIEMLSIF
	SapurV1A.0703s0020	SpYTHDF4B	AK------------FFIIKSYSEDN--VHK--SIKYGVWASTPNGNRKLDTT---YR---EAKEK---QDPCP-----VFLLFS--VNASAQFC-------------------------------------GVAEM---TGPVDFDKSV-DYWQ-QD-----------------------------------KW-NGQFPVKWHLIKDVPNSQ-FRHIVLENNDNK-PVTNSRDTQEVK-------------------------LEQGIEMLSIF
	SapurV1A.1271s0080	SpYTHDF5B	AK------------FFVIKSYSEDN--VHK--SIKYGVWASTPNGNKKLDAA---YR---EAKEN---HGTCQ-----IFLLFS--VNASAQFC-------------------------------------GVAEM---VGPVDFDKNV-DFWQ-QD-----------------------------------KW-SGKFPVKWHIIKDVPNSQ-FRHIVLENNDNK-PVTNSRDTQEVE-------------------------LEHGVEMLGIF
	SapurV1A.0430s0170	SpYTHDF6B	AK------------FFIIKSFSEDN--VHK--SIKYSIWASTPHGNKKIEAA---YR---EAKEK---EGNCP-----VFLLFS--VNASGQFC-------------------------------------GVAEM---VGPVDFEKDA-DYWQ-QD-----------------------------------RW-NGQFPVQWHIIKDVPNSR-FRHMLLENNDNK-PVTHSRDSQEVK------------------------LEQGIEMLKIF
	SapurV1A.0001s2340	SpYTHDF7B	AK------------FFIIKSFSEDN--VHK--SIKYNVWASTPHGNKKIDAA---YR---EAKEK--EGNCP-----VFLLFS--VNASGQFC-------------------------------------GVAEM---VGPVDFEKDA-EYWQ-QD-----------------------------------RW-NGQFPVQWHIVKDVPNSR-FRHILLENNDNK-PATHSRDSQEVK-------------------------LEQGIEMLKIF
	SapurV1A.0470s0280	SpYTHDF6C	AK------------FFVIKSYNEDD--VHK--SIKYDVWASTPNGNKKLDAA---FH---NAEVV-SSE--TSTKCP-----IFLFFS--VNGSGQFV-------------------------------------GLAEM---VGQVDFNKDM-DFWQ-ID-----------------------------------KW-NGFFPVKWHVIKDIPNGQ-LRHIVLENNDGH-SVTFSRDTQEIG-------------------------LEKGLEMLNIF
	SapurV1A.0081s0640	SpYTHDF1A	AK------------FFIIKSYSEDD--VHK--SIKYNVWASTPNGNKKLDAA---YQ---EAEQK-S--GCCP-----VFLLFS--VNTSGQFV-------------------------------------GLAEM---TGRVDFDKSV-EYWQ-QD-----------------------------------KW-TGYFPVKWHIVKDVPNSF-LKHITLDNNENK-PVTNSRDTQEVK-------------------------LEEGLKLIKIF
	SapurV1A.0699s0200	SpYTHDF2A	AK------------FFIIKSYSEDD--VHK--CIKYHVWASTPNGNKKLDAA---YQ---EAEQK-S--GGCP-----VFLLFS--VNTSGQFV-------------------------------------GLAEM---TGRVDFDKSV-EYWQ-QD-----------------------------------KW-TGYFPVKWHFVKDVPNSL-LKHITLENNENK-PVTNSRDTQEVK-------------------------LEHGLKVIKIF
	SapurV1A.0308s0100	SpYTHDF3A	AK------------FFVIKSFSEDD--VHK--SIKYSVWTSTPNGNKKLDAA---YK---QAKEN-Q---SDCP-----VFLLFS--VNTSGQFV-------------------------------------GLAEM---VGPVDFNKTV-EYWQ-QD-----------------------------------KW-TGCFPLKWHIIKDVPNGC-LRHITLENNENK-PVTNSRDTQEVI-------------------------FEKGVQILKIF

	SapurV1A.0668s0150	SpYTHDF4A	AK------------FFVIKSFSEDD--VHK--SIKYCVWTSTANGNKKLDAA---YK--QAKEN-PG---GGCP-----VFLLFS--VNTSGQFV--------------------------------------GLAEM---TGHVDFNKTV-EYWQ-QD-----------------------------------KW-TGCFPLKWHIIKDVPNGC-LRHITLENNENK-PVTNSRDTQEVM------------------------FEKGVQILKIF
	SapurV1A.2122s0020	SpYTHDF5A	AK------------FFVIKSFSEDD--VHK--SIKYCVWTSTANGNKKLDAA---YK--QAKEN-PG---GGCP-----VFLLFS--VNTSGQFV--------------------------------------GLAEM---TGHVDFNKTV-EYWQ-QD-----------------------------------KW-TGCFPLKWHIIKDVPNGC-LRHITLENNENK-PVTNSRDTQEVM------------------------FEKGVQILKIF
Brachypodium stacei	Brast07G208500	BstYTHDC1A	TR------------YFIVKSCNREN--LEI--SVQQGIWATQRSNEAKLNEA---FE--SMEN---VILIFS--INRTRNFQ--------------------------------------GCAKM---TSRIGGYIGG-GNWK-SA-----------------------NGTAH--------Y-GRNFSLQWLKLCELSFQK-THHLRNPYNDNL-PVKISRDCQELE------------------------PFIGEQLASLL
	Brast02G160500	BstYTHDF1C	AK------------YFVIKSYSEDD--VHK--SIKYNVWSSTPNGNRRLDAA---YS--EAQGR-NPW--K---CP-----IFLFFS--VNTSGQFC--------------------------------------GVAEM---VGLVDFHKDM-DFWQ-QD-----------------------------------KW-SGSFPVKWHLVKDVPNST-FRHIILENNENK-PVTNSRDTQEIP------------------------YKSGINMLALF
	Brast01G183400	BstYTHDF2C	AL------------FFVIKSYSEDD--IHK--SIKYNVWASTPNGNKRLDNA---YK--VAQER-MAG---KGTKCP-----VFLFFS--VNASGQFC--------------------------------------GVAEM---VGPVDFNRNM-NFWQ-QD-----------------------------------KW-NGFFPVKWHIIKDVPNPQ-FRHIILENNENK-PVTNSRDTQEVK------------------------FLQGAEMLNIF
	Brast08G195000	BstYTHDF3C	AK------------FFVIKSWGEAN--VHK--SVKYGVWSSSLQGNKKLDRA---FS--DAQLI-AAS--SSTKCP-----VFLFFS--VNQSNHFC--------------------------------------GVAEM---VGPVDFQKNM-DFWS-QD-----------------------------------RY-VGSFPVRWHIIKNIPNFA-LQYIVLQNNEHR-PVTFSRDTQEIP------------------------YGPGTSMLKIF
	Brast01G273700	BstYTHDF4C	AK------------FFVIKSIGEAD--IHK--SIKYGVWSSSSSGNSKLDGA---YR--DADRI-ARR---NSTKCP-----VFLFFS--VNGSGHFC--------------------------------------GLAEM---VGPVDFHKDM-DFWC-QD-----------------------------------KW-SGSFPVRWHIIKDVPNYT-LQHILLQNNENK-PVTHSRDTQEIP------------------------YIPGISMLKIL
	Brast03G001600	BstYTHDF1B	AK------------FFVIKSYTEDH--VHR--SIKYNVWASTASGNRKLDSA---YR--AAKEK---EDHCP-----IFLFFS--VNGSGQFC--------------------------------------GVAEM---IGPVDFDRSV-DYWQ-QD-----------------------------------KW-SGQFPVKWHIIKDVPNNL-LRHITLENNDNK-PVTNSRDTQEVK------------------------LEYGLQMLTIF
	Brast09G011900	BstYTHDF5C	AK------------FYMIKSYSEDD--IHK--GIKYNVWASTRNGNSKLDAA---FH--EAQNL-MKE--TGSKCP-----VFLFFS--VNTSGQFV--------------------------------------GLAEM---LGPVDFKKTM-EFWQ-QD-----------------------------------KW-NGFFPVIWHIVKDIPNRL-FKHITLENNDNR-PVTFSRDTQEIH------------------------LPQGLELLKIF
	Brast04G276500	BstYTHDF6C	AK------------FYMIKSYSEDD--IHK--GIKYNVWASTPNGNSKLNAA---FH--EAQNL-MEE---KGSKCP-----VFLFFS--VNTSGQFV--------------------------------------GLAEM---LGPVDFNKTM-DFWQ-QD-----------------------------------KW-NGFFPVIWHIVKDIPNRL-FKHITLENNDNR-PVTFSRDTQEIH------------------------LPQGLELLKNF
	Brast02G048000	BstYTHDF1A	AK------------FFVIKSYSEDD--IHK--SIKYNVWASTTNGNKKLDAA---YQ--EAQAK-S--SSCP-----IFLFFS--VNTSGQFV--------------------------------------GVAEM---TGPVDFEKTL-EYWQ-QD-----------------------------------KW-NGSFSVKWHIVKDVPNNI-LKHIILENNEGK-PVTNSRDTQDIN------------------------LEQGTQMLKIF

	Brast10G020600	BstYTHDF2A	AK------------FFVIKSYSEDD--VHK--SIKYNVWASTPNGNKKLDSG---YR--EAQEK-S--SECP-----VFLFFS---VNTSGQFV-------------------------------------GVAEM---VGPVDFDKTV-DYWQ-QD----------------------------------KW-NGCFSIKWHIVKDIPNNI-LKHITLDNNDNK-PVTNSRDTQEVK-------------------------LEQGLQMLKIF
	Brast02G336900	BstYTHDF3A	AK------------FFVIKSYSEDD--VHK--SIKYNVWASTPNGNKKLDTA---YQ--AANDE-S---SKSP-----VFLFFS---VNTSGQFV-------------------------------------GLAEM---VGPVDFNKTV-EYWQ-QD----------------------------------KW-TGCFPVKWHIVKDIPNNL-LKHIILEYNENK-PVTNSRDTQEVK-------------------------LEQGLQVLKIF
Panicum virgatum	Pavir.Db00395	PviYTHDC1Aa	SR------------YFIVKSCNREN--LEI--SVQQGIWATQRSNEAKLNEA---FE---STEN---VILIFS---INRTRHFQ-------------------------------------GCAKM---TSRIGGYIGG-GNWK-SA-----------------------HGTAH--------Y-GRNFSMQWLKLCELSFQK-THHLRNPYNDNL-PVKISRDCQELE-------------------------PFIGEQLASLL
	Pavir.Da00064	PviYTHDC1Ab	SR------------YFIVKSCNREN--LEI--SVQQGIWATQRSNEAKLNEA---FE---STEN---VILIFS---INRTRHFQ-------------------------------------GCAKM---TSRIGGYIGG-GNWK-SA-----------------------HGTAH--------Y-GRNFSMQWLKLCELSFQK-THHLRNPYNDNL-PVKVC
	Pavir.Ea02386	PviYTHDF1C	AL------------FFVIKSYSEDD--IHK--SIKYNVWASTPNGNKRLDNA---YR--LAQER-MAE---KGTKCP-----VFLFFS---VNASGQFC-------------------------------------GVAEM---VGAVDFNRNM-SFWQ-QD----------------------------------KW-NGFFSVKWHIIKDVPNPQ-FRHIILENNENK-PVTNSRDTQEVK-------------------------FPQGTEMLNIF
	Pavir.Eb02750	PviYTHDF2C	AL------------FFVIKSYSEDD--IHK--SIKYNVWASTPNGNKRLDNA---YR--LAQER-MAE---KGTKCP-----VFLFFS---VNASGQFC-------------------------------------GVAEM---VGPVDFNRNM-NFWQ-QD----------------------------------KW-NGFFSVKWHIIKDVPNPQ-FRHIILENNENK-PVTNSRDTQEVK-------------------------FPQGTEMLNIF
	Pavir.J03441	PviYTHDF3C	AK------------FFVIKSYNESD--VHK--SIKYGVWSTSSVGNRKLDAA---FR--EAQAI-AAS---SSTLCP-----VFLFFS---VNESFNFC-------------------------------------GVAEM---VGPVDYQNDM-DFWC-KD----------------------------------KW-TGSFPVKWHIIKNIRNYT-FRSILLQNNEYK-RVTWSRDTQEIH-------------------------YTPGTTMLELF
	Pavir.Eb02265	PviYTHDF4C	AK------------FFVIKSIGEAD--VHK--SIKYGVWSSSSNGNSKLDSA---FR--DADRI-SRR---NSTKCP-----VFLFFS---VNGSGHFC-------------------------------------GMAEM---VGPVDFHKDM-DFWC-QD----------------------------------KW-TGCFPVRWHIVKDIPNYC-LQHITLQNNENK-PVTHSRDTQEVP-------------------------YIPGMSVLKIF
	Pavir.Ea01248	PviYTHDF5C	AK------------FFVIKSIGEAD--VHK--SIKYGVWSSSSNGNSKLDSA---FR--DADRI-SRR---NSTKCP-----VFLFFS---VNGSGHFC-------------------------------------GVAEM---VGPVNFHKDM-DFWC-QD----------------------------------KW-TGCFPVRWHIVKDIPNYC-LQHITLQNNENK-PVTHSRDTQEIP-------------------------YIPGMSVLKIF
	Pavir.J39541	PviYTHDF1B	AR------------FFIIKSYSEDN--VHK--SVKYGVWASTTNGNKKLDSA---YC--EAKEK---EEHCP-----IFLLFS---VNASAQFC-------------------------------------GVAEM---IGPVDFEKSV-DYWQ-QD----------------------------------KW-TGQFPVKWHIVKDVPNNL-FRHIILENNDNK-PVTNSRDTQEVK-------------------------LEQGLEMLKIF

	Pavir.Fa00200	PviYTHDF2B	AR------------FFIIKSYSEDN--VHK--SVKYGVWASTTNGNKKLDSA---YR---EAKEK---EEHCP-----IFLLFS---VNASAQFC-----------------------------------GVAEM---IGPVDFEKSV-DYWQ-QD----------------------------------KW-TGQFPVKWHIVKDVPNNL-FRHIILENNENK-PVTNSRDTQEVK------------------------LEQGLEMLKIF
	Pavir.Fa01762	PviYTHDF3B	AK------------FFVIKSYTEDH--VHR--SIKYSVWASTASGNRKLDSA---YR---AAKEK---EEHCP-----IFLFFS---VNGSGQFC-----------------------------------GVAEM---IGPVDFDRSV-DYWQ-QD----------------------------------KW-SGQFPVKWHIIKDVPNNL-LRHIILENNDNK-PVTNSRDTQEVK------------------------LEQGLQMLTIF
	Pavir.Fb00853	PviYTHDF4B	AK------------FFVIKSYTEDH--VHR--SIKYSVWASTASGNRKLDSA---YR---AAKEK---EEHCP-----IFLFFS---VNGSGQFC-----------------------------------GVAEM---IGPVDFDRSV-DYWQ-QD----------------------------------KW-SGQFPVKWHIIKDVPNNL-LRHIILENNDNK-PVTNSRDTQEVK------------------------LEQGLQMLTIF
	Pavir.J38606	PviYTHDF5B	AK------------FFVIKSYTEDH--IHK--SIKYNVWASTATGNRKLNAA---YR---EAKDK---EELMA-----VV---TGQFC------------------------------------GVAEM---IGPVDFDMSV-DYWQ-ND----------------------------------RW-SGQFPVKWHIVKDVPNNL-VRHIILGNNKDK-RVTNSRDTQEVK------------------------LEQGVQMLAIF
	Pavir.J11462	PviYTHDF6B	AK------------FFVIKSYTEDH--VHK--SIKYNVWASTARGNRKLNAA---YR---EAKDK--EEYCP-----IFLFFS---VNGSGQFC-----------------------------------GVAEM---IGPVDFDKSV-DYWQ-ND----------------------------------KW-SGQFPVKWHIVKDVPNNL-VRHIILENNEDK-RVTNSRDTQEVK------------------------LEQGVQMLAIF
	Pavir.Ca00545	PviYTHDF6C	AK------------FFMIKSYSEDD--IHK--GIKYNVWASTANGNDKLDAA---YH---EAQIL-MKE--NGENCP-----VFLFFS---VNTSGQFV-----------------------------------GLAEM---LGPVDFKKTM-DFWK-GD----------------------------------KW-NGFFPIIWHIIKDIPNRL-FKHIILEHNDYR-PVTFSRDTQEIG------------------------LAQGMQMLKIF
	Pavir.Ia04185	PviYTHDF1A	AK------------FFVIKSYSEDD--IHK--SIKYNVWASTTNGNKKLDAA---YQ---EAQSK-G---SACP-----IFLFFS---VNTSGQFV-----------------------------------GVAEM---TGAVDFEKTL-EYWQ-QD----------------------------------KW-NGSFSVKWHIVKDVPNNI-LKHIILENNENK-PVTNSRDTQEIH------------------------LEQGLQMLKIF
	Pavir.Ib00367	PviYTHDF2A	AK------------FFVIKSYSEDD--IHK--SIKYNVWASTTNGNKKLDAA---YQ---DSQSK-G---SACP-----IFLFFS---VNTSGQFV-----------------------------------GVAEM---TGAVDFEKTL-EYWQ-QD----------------------------------KW-NGSFSVKWHIVKDVPNNI-LKHIILENNENK-PVTNSRDTQEIH------------------------LEQGLQMLKIF
	Pavir.Ca01254	PviYTHDF3A	AK------------FFVIKSYSEDD--VHK--SIKYNVWASTPNGNKKLDAG---YR---EAQEK-S---SDCP-----VFLFFS---VNTSGQFV-----------------------------------GVAEM---VGPVDLDKTV-EYWQ-QD----------------------------------KW-NGCFPLKWHIVKDVPNNI-LKHITLDNNDNK-PVTNSRDTQE
	Pavir.J30385	PviYTHDF4A	AK------------FFVIKSYSEDD--VHK--SIKYNVWASTPNGNKKLDAG---YR---EAQEK-S---SDCP-----VFLFFS---VNTSGQFV-----------------------------------GVAEM---VGPVDLDKTV-EYWQ-QD----------------------------------KW-NGCFPLKWHIVKDVPNNI-LKHITLDNNDNK-PVTNSRDTQE
	Pavir.Ia00570	PviYTHDF5A	AK------------FFVIKSYSEDD--VHK--SIKYNVWASTPNGNKKLDAG---YR---EAQEK-S---SDCP-----VFLFFS---VNTSGQFV-----------------------------------GVAEM---VGPVDFDKTV-EYWQ-QD----------------------------------KW-NGCFPLKWHIVKDVPNNI-LKHITLDNNDNK-PVTNSRDTQEVK------------------------LEQGLEMLKIF
	Pavir.J01552	PviYTHDF6A	AK------------IFVIKSYSEDD--VHK--SIKYNVWASTPNGNKKLDAG---YR---EAQEK-S---SDCP-----VFLFFS---VNTSGQFV-----------------------------------GVAEM---

			VGPVDFDKTV-EYWQ-QD--------------------------------KW-NGCFPLKWHIVKDVPNNI-LKHITLDNNDNK-PVTNSRDTQEVK-----------------------<br>LEQGLEMLKIF
	Pavir.Ba03744	PviYTHDF7A	AK------------FFIIKSYSEDD--VHK--SIKYNVWASTPNGNKKLDAA---YQ---EAKEK-S--------------------------<br>---------------SETP-----VFLLFS--VNASGQFV---------------------------------GLAEM---<br>VGRVDFNKTV-EHWQ-QD--------------------------------KW-TGCFPVKWHIVKDVPNSL-LKHIILENNENK-PVTNSRDTHEVK-----------------------<br>LEQGLQVLKIF
	Pavir.J38005	PviYTHDF8A	AK------------FFIIKSYSEDD--VHK--SIKYNVWASTPNGNKKLDAA---YQ---EAKEK-S--------------------------<br>---------------CESP-----VFLLFS--VNASGQFV---------------------------------GLAKM---<br>VGRVDFNKTV-EHWQ-QD--------------------------------KW-TGCFPVKWHIVKDVPNSL-LKHIILENNENK-PVTNSRDTHEVK-----------------------<br>LEQGLQVLKIF
Micromonas pusilla	MicpuC2.fgenesh1_pg.C_scaffold_8000034	MpYTH	VR------------FFVIKSFAEED--VRK--SVKHGCWTSTSQGNARLDAA---WRGEDVFTFEPHGD-----DEN-GDDERERE------<br>REGEGEGETAAAAAAAAAADSSSGSGSGSGSDPASDAEPA-SDSDEPSVRSVDTQTPGPPLLDDATPATAESATKTTIAPCSRPGSSGAPPSKPRVILFFS-----------<br>---VNSSGHFC----------------------------------GVAEM---TSPVD----------DDAVDATLLP---------------PAAA------<br>ASW-PGRFAVKWHIVKDVPNTA-LRHIRVCAGDKK-PVPNSRDAQEIE-----------------------PAQGALVLNIF
Micromonas commoda	EuGene.1100010578	McYTH	VR------------FFVMKSHGEDD--VHR--SLRYGWWSSTQAGNARLYAA---FRGLDAGEGESLGDLPKTLDESCGMDDLDRAFIVNAVKEDGDETEVLAP------------<br>---DLSDISRDSEESDSPSPAGPL-------------EASTADAAAADRAT-----SKDRAETP----RVVLFFS--<br>VNCSGHFC-----------------------------------GVAEM---IGPVE---RI-DPTAPRDAHLRSGGGGGGGGGGGGGGGPRRFPAASMLTNGFALA-<br>DGRFKVRWHWVKDVPNTV-LRHIRLVAGNEK-PVTNSRDAQEVE-----------------------PGQGAMVLSVF

*All accession numbers relate to the Uniprot, NCBI, and Phytozome databases

** In cases when a name was already attributed to a protein, this name was kept with the new name, refering to the species of origin and to the phylogenetic classification of the protein, added in parenthesis

Supplementary Table S4 the relative expression level of 77 organismal-development-related genes used for heatmap analysis.

gene_id	gene symble	Log2FC		
		osythdf1a	osythdf2a	osythdf3a
LOC_Os02g13310		-9.5619	-1.0855	-4.1578
LOC_Os03g51710	*OSH3*	-8.9869	-1.3526	-7.0396
LOC_Os01g46870		-8.9168	-1.5943	-5.3881
LOC_Os04g45330	*OsYABBY5; OsYAB3; TOB1*	-8.615	-1.2943	-4.3509
LOC_Os12g36890	*OsCSLD4; NRL1; ND1; sle1; DNL1; OsCD1*	-8.0836	-1.1736	-5.0574
LOC_Os09g34930		-7.8626	-1.2858	-3.9654
LOC_Os03g14080	*OsAUX2*	-7.7898	-1.3768	-4.9957
LOC_Os07g03250	*Crl-5*	-7.7566	-1.3525	-4.6714
LOC_Os01g33040	*DBS1; OsNACK*	-7.5488	-1.2459	-3.6439
LOC_Os04g48510	*OsGRF12*	-7.4847	-1.2526	-4.1748
LOC_Os04g51000	*RFL; APO2*	-7.3429	-2.1784	-6.2554
LOC_Os10g05690	*OsAUX4*	-7.318	-1.1632	-3.9296
LOC_Os04g56850	*OsARF11; OsMP; OsARF5*	-7.3041	-1.0846	-4.1656
LOC_Os01g67100	*FSM;CAF-1*	-7.1895	-1.0183	-3.4899
LOC_Os10g26340	*PLA1*	-7.0451	-1.0395	-7.8152
LOC_Os08g40170	*CDKB2;1; cdc2Os3*	-6.9668	-1.1371	-3.5603
LOC_Os08g41360		-6.9401	-3.3398	-2.7567
LOC_Os01g62605		-6.7356	-1.1936	-4.7526
LOC_Os04g28620		-6.6957	-5.648	-7.9919
LOC_Os11g04190	*OsPIN1c; OsPIN1b*	-6.5802	-1.191	-3.375
LOC_Os03g10040		-6.2745	-1.7185	-4.2933
LOC_Os02g03120		-6.2414	-2.663	-2.3359
LOC_Os03g56050		-6.1617	-1.3587	-5.2617
LOC_Os06g10310	*OsGRF2*	-6.1049	-1.2917	-5.0023
LOC_Os12g10670		-6.0781	-1.3261	-3.8577
LOC_Os05g27980		-6.0617	-1.3375	-3.7453
LOC_Os02g28850		-5.9803	-1.1244	-3.4852
LOC_Os03g41600		-5.8846	-2.1204	-1.9548
LOC_Os03g11600	*DL; OsYABBY*	-5.8114	-1.7158	-3.8138
LOC_Os04g38570	*OsABCB14*	-5.7616	-1.4281	-3.2766
LOC_Os12g38400	*OsMYB91*	-5.7002	-1.2739	-4.2461

LOC_Os03g49880	*OsTB1; FC1; SCM3*	-5.5398	-1.7117	-6.3093
LOC_Os04g02640		-5.3723	-1.5771	-6.279
LOC_Os04g33560		-5.3588	-1.0619	-3.1647
LOC_Os08g42600	*OsRBR1; PIS1*	-5.3002	-1.0986	-3.0869
LOC_Os04g28260		-5.2725	-1.0827	-2.8998
LOC_Os06g36680		-5.2476	-1.8521	-3.2175
LOC_Os12g03130		-4.9757	-1.1114	-3.9286
LOC_Os01g15480		-4.6478	-1.1559	-3.0775
LOC_Os04g13140		-4.363	-1.2208	-2.5563
LOC_Os01g07480	*OsCrll4*	-4.0908	-1.1631	-1.6119
LOC_Os04g43300		-4.0777	-1.0175	-2.418
LOC_Os07g49140		-3.9888	1.3961	-3.0252
LOC_Os09g36160		-3.8437	-1.1961	-4.6417
LOC_Os09g25100		-3.741	-1.2983	-5.5075
LOC_Os01g55560		-3.7138	-1.5581	-3.2888
LOC_Os07g07920		-3.5831	1.4895	-2.6613
LOC_Os04g46580	OSSPL7	-3.4951	-1.635	-3.2
LOC_Os10g25620		-3.3118	-4.0817	-2.3927
LOC_Os02g46970		-3.2992	-1.1604	-4.2757
LOC_Os06g49830		-3.1737	-1.2646	-5.8352
LOC_Os08g31030		-2.9736	1.087	-1.2371
LOC_Os07g17210		-2.7093	2.2462	-1.8848
LOC_Os03g02800	*RIM1*	-2.645	1.0142	-2.1354
LOC_Os06g15020		-2.2586	1.6406	1.5527
LOC_Os01g12440		-2.2399	-1.8037	-2.0165
LOC_Os01g34560		-2.2126	1.3549	-1.1105
LOC_Os01g66100	*sd1; OsGA20ox2; qSD1-2; qPA1*	-2.1301	-1.0491	-1.9423
LOC_Os03g58600	*OsMEL1*	-2.1155	1.2839	-2.1407
LOC_Os02g58490	*OsAGO1c*	-1.8356	-1.2321	-1.1439
LOC_Os04g47080		-1.7186	1.081	2.543
LOC_Os05g44090		-1.5961	-1.6446	-2.3388
LOC_Os01g66590	*OsCrll3; OsIG1; OsAS2*	-1.5556	-1.0164	-2.4638
LOC_Os06g11330		-1.2077	-1.1194	-1.0372
LOC_Os11g35710		-1.0678	3.5252	-1.6476
LOC_Os01g18360		1.1451	1.7689	2.0873
LOC_Os01g25010		1.3611	1.1524	1.8913
LOC_Os04g40140		1.4495	2.645	1.5658

LOC_Os06g19540		1.5108	1.6758	1.6269
LOC_Os01g58860	*OsPIN9*	1.5857	2.1314	1.4208
LOC_Os02g12350		1.6883	2.0187	2.0254
LOC_Os03g59600		1.8122	2.3614	1.9622
LOC_Os01g58194		1.8911	1.3941	1.3227
LOC_Os08g29660		2.2899	1.0293	1.0592
LOC_Os01g50100		2.8393	1.6911	1.4538
LOC_Os01g64470		2.9377	1.9662	1.4414
LOC_Os04g12960		2.9503	3.1572	1.1924

Supplementary Table S5 Primers used in this study

Primer used for qPCR analysis	
RT-DF3C-2F	GTGAAGCCACAGGGTAGTAATG
RT-DF3C-2R	GTCTGTTCTCTTTCCCGTCATC
RT-DF1C-1F	TCCTCGAAGACTGCAAACAAGGC
RT-DF1C-1R	ATGCACCGCAAACTTGCTAGAC
RT-DF1A-1F	AGCCGTGACACACAAGAGGTAAAC
RT-DF1A-1R	TCCTGCATCAACTTCTGACGGTTC
RT-DF2C-2F	GAAGCGAAAGGCAATGGTAATG
RT-DF2C-2R	GGCTCACCTGCCACATTTA
RT-DF2A-1F	AAGCTGATGCAGGAGAAGAGAGC
RT-DF2A-1R	CATTGTCAAGAGAGCCCTGTCC
RT-DF5C-2F	GAGGCACATCCACAGCTAAA
RT-DF5C-2R	GCCCATCTTGAACCTCTGATTA
RT-DF3B-1F	CATTGGCTTCTGGAATGCATCAGG
RT-DF3B-1R	TTGCTGCCGTGCCTTGTATGAC
RT-DF4C-2F	TCAGCAAGGTTCCATCTCTTC
RT-DF4C-2R	GGTCAGCCTTTGTGCATTTC
RT-DC1A-1F	GGTGAGCAATTGGCTTCACTGC
RT-DC1A-1R	GCTGCAATTAGTATAGCCGTCAGC
RT-DF3A-1F	AGACGTCGTTACTGGATGACTTTG
RT-DF3A-1R	GCAGCTTCTCATCAACGACCTTC
RT-DF2B-2F	AGAAAGCCTTGCAGGAGAATAG
RT-DF2B-2R	GTCTGAGATGTGGGCCATAAG
RT-DF1B-2F	CTGATAACCCTGAAGAGCAGAC
RT-DF1B-2R	GCTTGTCCTTCACTTGGTAGAT
RT-DF3C-1F	TCTAGTGGAAACAGCAAACTGGAC
RT-DF3C-1R	AGAAGAGCCCACTACCATTCACG
RT-OE-2-F	CTGAGATGGTCGGTCCGGTAGAT
RT-OE-2-R	TCCCTGCTGTTTGTCACAGGTTT
RT-OE-3-F	GGCAGTTTGTTGGTGTTGCTGAA
RT-OE-3-R	CGGCTATTTGTCACTGGCTTGTTC
RT-DF2C-1F	AGGAAGAGCTCTGGGAAAGTGTC
RT-DF2C-1R	AACTGCCCGCTAGCATTAACAG
RT-OE-7-F	GATGATTGGACCTGTGGACTTCG
RT-OE-7-R	AGGCTGTTCGGGACATCCTTGAT

RT-OE-8-F	CAGTGGGCAGTTTGTAGGATTAG
RT-OE-8-R	AACCGATTTGGAATGTCCTTTATA
Primer used for vector construction	
DF3C-CF	AGATGATCCGTGGCATTATCCGTATGGTTATACGTTTTAGAGCTATGC
DF3C-CR	GCATAGCTCTAAAACGAAAATAGATGTCTTGGCCTGAGCCTCAGCGCAGCAGCTTA
DF1C-CF	AGATGATCCGTGGCAATATCCATTTTCATACCCGTTTTAGAGCTATGC
DF1C-CR	GCATAGCTCTAAAACGATTTTGTTACGAAGTATCTGAGCCTCAGCGCAGCAGCTTA
DF1A-CF	AGATGATCCGTGGCACGTCGCGCCGCCTCAGGTCCGTTTTAGAGCTATGC
DF1A-CR	TTCTAGCTCTAAAACGGACCTGAGGCGGCGCGACGCTGAGCCTCAGCGCAGCAGCTTA
DF2C-CF	AGATGATCCGTGGCAATCACACTAATTTTGTGGGTTTTAGAGCTATGC
DF2C-CR	GCATAGCTCTAAAACTATTGTATTGGTCAGGATCTGAGCCTCAGCGCAGCAGCTTA
DF2A-CF	AGATGATCCGTGGCAGTCGATCACTCCCCTGCTCCGTTTTAGAGCTATGC
DF2A-CR	TTCTAGCTCTAAAACAACAGTTGGGACAGGAGAACCTGAGCCTCAGCGCAGCAGCTTA
DF5C-CF	AGATGATCCGTGGCATATCATTCAGAAGAACGCGTTTTAGAGCTATGC
DF5C-CR	GCATAGCTCTAAAACGTATCTTGGATCTTGTTGCTGAGCCTCAGCGCAGCAGCTTA
DF3B-CF	AGATGATCCGTGGCATCTTCCCATTGACCCAGAGTTTTAGAGCTATGC
DF3B-CR	GCATAGCTCTAAAACGTGCAGATGGAACTGATGCTGAGCCTCAGCGCAGCAGCTTA
DF4C-CF	AGATGATCCGTGGCAACCAACATCCTCAGTGCAGTTTTAGAGCTATGC
DF4C-CR	GCATAGCTCTAAAACTTGCATTCTGGTCAGTGGCTGAGCCTCAGCGCAGCAGCTTA
DC1A-CF	AGATGATCCGTGGCAGCACTCGTCCGACCCCGGGTTTTAGAGCTATGC
DC1A-CR	GCATAGCTCTAAAACGGGGATGGCGGCGGGCACCTGAGCCTCAGCGCAGCAGCTTA
DF3A-CF	AGATGATCCGTGGCATCCACATACCTGAGCCATGAGTTTTAGAGCTATGC
DF3A-CR	TTCTAGCTCTAAAACGAAGTTGAATAACCATATGGCTGAGCCTCAGCGCAGCAGCTTA
DF2B-CF	AGATGATCCGTGGCAAGAGTGCTGCTAAAGAGCGTTTTAGAGCTATGC
DF2B-CR	GCATAGCTCTAAAACGGAGATAACATGATTGACCTGAGCCTCAGCGCAGCAGCTTA
DF1B-CF	AGATGATCCGTGGCATATCAATCTACCAAGTGAGTTTTAGAGCTATGC
DF1B-CR	GCATAGCTCTAAAACCTTGTGTTGGGTCAATCGCTGAGCCTCAGCGCAGCAGCTTA
SacIL-DF2CF	TTCTGCACTAGGTACCAGGCCTGTTCAGCCCACCACAGATTTCA
SacIR-DF2CR	CTGACGTAGGGGCGATAGAGCTCTGTTGCTTTGCCTTGTCCACT
SnaBIR-DF2CF	CGGGGATCCGTCGACTACTTCAGCCCACCACAGATTTCA
SnaBIL-DF2CR	AGGTGGAAGACGCGTTACTGTTGCTTTGCCTTGTCCACT
SacIL-DC1AF	TTCTGCACTAGGTACCAGGCCTGGCATACAAGCACTCCTACGACGAT
SacIR-DC1AR	CTGACGTAGGGGCGATAGAGCTCCGACGAGCCATTTGGAACAGA
SnaBIR-DC1AF	CGGGGATCCGTCGACTACGCATACAAGCACTCCTACGACGAT
SnaBIL-DC1AR	AGGTGGAAGACGCGTTACCGACGAGCCATTTGGAACAGA
DF3C-dn-F	TCCGGAGCTAGCTCTAGAATGCGAGTGGACTATCCCTTT

DF3C-dn-R	CCTCGAGACGTCTCTAGACTTCACTTTGATAGCCTT
DF1C-dn-F	TCCGGAGCTAGCTCTAGAATGGTTACGAAGTATGATCAA
DF1C-dn-R	CCTCGAGACGTCTCTAGATTTACATGAAAAGTTCTT
DF1A-dn-F	TCCGGAGCTAGCTCTAGAATGCCTGTCCAATATGATGAT
DF1A-dn-R	CCTCGAGACGTCTCTAGACTTACTGACATGCTCCTT
DF2C-dn-F	TCCGGAGCTAGCTCTAGAATGCCTGTTGATCATCCAGAT
DF2C-dn-R	CCTCGAGACGTCTCTAGATGTCAATGGGCCATCCTT
DF3B-dn-F	TCCGGAGCTAGCTCTAGAATGGTTATTGAGTATGAGGAT
DF3B-dn-R	CCTCGAGACGTCTCTAGACTCGACCTCATGGTTCTT
DF4C-dn-F	TCCGGAGCTAGCTCTAGAATGACAGTTCAGTATGAGCAT
DF4C-dn-R	CCTCGAGACGTCTCTAGATCCCTGGTGATATGCCTT
primer used for PCR sequencing	
CRAC-DF2B-F	CGACTCTGGGTAATCGGAGGAGG
CRAC-DF2B-R	TAGGAGAGGCTGGTGGCTGGTAGT
CRAC-DF3C-F	TTGACTTTTCCAGGTTACCAAACTT
CRAC-DF3C-R	AACAGGAGGAACTGAGGGTAATG
CRAC-DF1B-F	GGATTCGCAGGAGCCAAGAGTTG
CRAC-DF1B-R	TGAGGTGAAGCCGCAGGTGATG
CRAC-DF1C-F	GCCATATCTGTGGCATCAGGATT
CRAC-DF1C-R	AAGGTTTGGACGCCTCCTATTCT
CRAC-DF2C-F	CAGGGTCTTTATTATCCAGCAAC
CRAC-DF2C-R	ACAGGCTTGTTCTCGTTATTCTC
CRAC-DF5C-F	AAATGTCATCGGGAAGTGGGTAT
CRAC-DF5C-R	GGATTACATGCAAGAAAATGAGGAC
CRAC-DF3B-F	TTGGGTAAAGGTGGAGAACAACA
CRAC-DF3B-R	CCCAGGAGTTGGTGAATACAGAAG
CRAC-DF4C-F	AAGCCAGGACACTTGGAAGAGTATA
CRAC-DF4C-R	CAAAGGAGGGATGGCATTATTCT
CRAC-DC1A-F	GTCCCAAGTCCCAACTCCCAAC
CRAC-DC1A-R	CTCCAACCCAAGAGCTAACAAACG

Supplementary Table S6 Log2 expression changes of abiotic response genes in OsYTH mutants.

gene_id	gene symble	Log2FC									
		osythdf1a	*osythdf2a*	*osythdf3a*	*osythdf1b*	*osythdf2b*	*osythdf3b*	*osythdf1c*	*osythdf3c*	*osythdf4c*	*osythdf5c*
LOC_Os11g04020	OsTOM1	3.2792	7.4229	4.4971	-10.2747	-7.1067	-1.8743	-10.7481	-8.1509	-1.2217	-8.441
LOC_Os11g26790	RAB21; Rab16A	-0.7541	0.655	-4.6795	-2.1252	-6.0341	-4.334	-0.9926	-3.8629	-6.5668	-7.2578
LOC_Os01g72370	OsIRO2	-1.0762	4.1649	3.7685	-3.4543	-3.26	0.1071	-6.3446	-6.929	0.1176	-2.3838
LOC_Os03g17790	OsRCI2-5	0.3925	0.7344	-0.7367	-4.1495	-5.0063	-2.5996	-3.7051	-2.0998	-3.9781	-1.8866
LOC_Os03g12820	OsSRO1c; BOC1	0.2927	0.9939	-1.0999	-4.1682	-3.8594	-2.9352	-3.3651	-4.2107	-4.9093	-2.6602
LOC_Os02g10760	OsWR1	-0.1569	1.6293	0.2219	-2.7727	-1.758	-1.0787	-4.462	-0.3951	-1.1819	-1.0965
LOC_Os04g28420	rFKBP65	-0.0126	1.9587	1.2482	-1.9999	-1.9103	-1.6581	0.0152	-0.7631	-2.3771	-0.5023
LOC_Os12g39400	ZFP252; RZF71	-0.8923	-0.3044	-1.9578	-2.7631	-1.7294	-1.0259	-2.4661	-2.6747	-1.319	-2.3747
LOC_Os01g04800	OsRAV2	0.798	-0.272	-0.7896	-2.0584	-1.0062	-1.7092	-0.2974	-2.1543	-2.1038	-2.4577
LOC_Os05g34830	OsNAC52	1.0026	-0.0645	-0.0199	-2.9351	-1.6469	-1.1573	-0.1372	-3.4641	-2.1139	-1.4226
LOC_Os03g44380	OsNCED3	2.0449	-2.3295	1.1522	-1.3779	-0.0272	-0.7932	0.868	-3.2871	-4.9982	0.3461
LOC_Os03g32230	bsr-d1; ZOS3-12	6.1221	2.1551	1.6753	-1.112	-1.3549	-4.0854	1.9422	-2.2745	-3.5644	-1.0873
LOC_Os02g41510	OsMYB30	5.2242	1.7389	1.669	-0.3266	0.7217	-3.2884	2.0153	-1.1565	-0.518	-1.2932
LOC_Os01g61080	OsWRKY24	3.7233	0.1532	1.1274	-0.8505	0.7542	-1.4353	3.2491	-1.8999	-2.7649	-1.7122
LOC_Os07g26640		2.0774	3.1019	2.0239	1.5806	4.3036	0.2312	1.7276	3.3047	3.4691	-1.2601
LOC_Os02g55890	OVP3	0.7099	2.2184	0.8049	2.6642	2.6268	-1.1854	1.3301	3.3235	3.3431	0.0764
LOC_Os07g26660		1.2386	2.0218	0.9646	1.1967	2.1181	-2.6716	-2.2566	2.9063	0.5398	0.3232
LOC_Os03g20370	OsCam1-1	1.2014	-0.4156	0.5156	1.5053	1.0869	-1.1379	1.4713	1.4145	-0.3582	0.6861
LOC_Os02g47470	OsABA8ox1	2.0725	-1.1284	0.3294	1.0176	2.7681	-1.5304	3.2839	2.6201	-0.2035	0.3795
LOC_Os06g29790	OsPHO1;3	1.3371	-0.0175	0.4666	1.4351	1.2341	1.4791	0.7457	0.7633	0.1743	0.2197
LOC_Os05g51830	OsHDT1;HDT701	-0.5294	-0.5258	-0.4	2.5687	1.6139	0.1734	1.5598	2.6592	1.0175	1.756
LOC_Os02g53130	OsNR2; qCR2	0	-1.6365	0.5677	2.0279	4.1125	-1.5243	4.0553	2.1721	-3.1336	2.7605
LOC_Os04g51820	OsHKT1;1; OsHKT4	4.0549	1.4687	3.1727	0.314	1.3998	-0.9731	1.8912	0.1022	-0.6739	1.5438
LOC_Os06g48800	OsHKT2;4; OsHKT9	3.1048	-0.1044	2.5841	-0.0156	1.255	0.9039	1.8172	-1.6154	-1.2601	1.5969
LOC_Os02g52150	OsHSP24.1	1.4966	1.7964	1.6875	0.0918	0.3772	-0.9207	1.802	0.6593	-0.6719	1.2121
LOC_Os02g07830	OsHKT1;3; OsHKT6	1.7307	1.0571	1.8045	0.38	0.776	0.2489	1.4869	-0.3678	-0.5899	1.3482
LOC_Os02g10780	OsSPX2	2.3781	1.6273	1.7887	0.5156	0.5315	0.1733	0.2543	-1.5674	-0.5151	1.1946
LOC_Os04g01740	OsHSP1	-4.1178	1.5218	-1.864	-1.5222	-4.3195	-0.4964	-1.3461	-2.0332	-2.078	-2.3704
LOC_Os02g56920	OsGL1-6	-5.5386	-0.3876	-2.648	-1.8512	-3.6289	1.0069	-5.2235	-0.1738	0.3328	1.4248
LOC_Os06g40150	OsWR2	-7.1935	0.3689	-3.9251	-1.7815	-1.4235	-1.5792	-6.3569	-0.1031	-0.7874	-1.3909
LOC_Os01g15830	OsPOX1; ddOs319	-2.9293	0.2039	-1.7504	-1.0154	-1.7262	-1.1592	-2.573	0.7427	0.2868	-0.3067

LOC_Os06g12230	OsTCP19	-2.373	-1.6345	-2.4699	-0.2134	-1.2238	0.4586	-0.7745	0.6985	0.7167	-0.4599
LOC_Os03g06970	OsSDI1	-2.8728	1.0974	-2.9134	-0.7493	-0.9865	0.7087	-1.5789	-1.506	0.7097	-1.1904
LOC_Os02g58220	OsRPA2	-7.2101	-0.7715	-3.5271	1.995	-1.3754	-0.072	-4.3319	2.3253	1.4308	-2.4158
LOC_Os12g38400	OsMYB91	-5.7002	-1.2739	-4.2461	1.2356	0.0297	0.2821	-0.263	1.8048	0.8423	0.558
LOC_Os05g01730	OsDi19-6	-3.9695	-0.1226	-3.0293	2.1234	-1.0876	-1.1294	-1.6512	1.8631	0.3397	-0.4102
LOC_Os02g18410		-5.9437	-0.3254	-4.3456	2.4406	1.4235	-1.6656	0.237	3.3962	1.4854	0.5697
LOC_Os02g41904	CAL1	-8.2648	-1.4899	-3.6824	5.8619	-0.0508	-2.0616	-2.6434	5.5899	5.2243	-3.1448
LOC_Os01g56940	OsEXO1	-5.418	-1.4427	-3.7897	3.7485	-1.23	1.0147	-1.0311	3.8296	3.0369	-1.2945
LOC_Os10g05690	OsAUX4	-7.318	-1.1632	-3.9296	4.6849	-0.6765	0.4562	-2.3334	4.6487	3.8861	0.9784

Supplementary Table S7 Log2 expression changes of biotic-response genes in OsYTH mutants.

gene_id	gene symble	Log2FC									
		osythdf1a	*osythdf2a*	*osythdf3a*	*osythdf1b*	*osythdf2b*	*osythdf3b*	*osythdf1c*	*osythdf3c*	*osythdf4c*	*osythdf5c*
LOC_Os09g25070	OsWRKY62	0.0533	1.5696	0.8971	-4.9948	-2.0432	1.0718	-3.6035	-3.4029	2.0691	-1.7991
LOC_Os12g12010		-2.9448	-0.6273	-0.1078	-1.7396	-1.3692	-2.5783	-0.7713	-1.8189	-1.7731	-0.5742
LOC_Os12g11370		-0.4841	0.0032	-0.1538	-1.3639	-2.0095	-2.8819	-1.5858	-1.2705	-1.8403	-1.4848
LOC_Os12g17490		0.0447	0.0132	0.3972	-2.0656	-2.7488	-3.1688	-2.4434	-2.5546	-2.3857	-2.1736
LOC_Os12g12130		3.3146	0.8731	0	-3.4064	-3.4094	-3.7111	-2.8352	-3.4961	-3.3526	-3.21
LOC_Os02g14430	OsPrx30	1.1716	-2.573	-0.7368	-1.8581	-1.6216	-2.7997	-3.0237	-4.0243	-1.8807	-2.8318
LOC_Os10g10130	OsWAK112d	0.8629	-1.0833	0.2204	-3.275	-1.789	-0.1616	-2.0453	-4.9201	-2.8534	-3.2814
LOC_Os08g39840	OsHI-LOX; RLL; OsLOX9	3.5757	1.7762	1.5257	-4.5029	-2.5147	-1.7191	-0.8563	-7.079	-5.7451	-4.5283
LOC_Os08g04500	OsTPS3	2.5124	-1.0556	2.5999	-0.8625	-0.0785	0.7725	1.3457	-4.3147	-4.9098	0.6238
LOC_Os10g25230	OsJAZ13; OsTIFY11e	3.9878	-1.7234	1.7672	-1.071	0.6581	-3.9918	1.6437	-4.6038	-5.0541	-1.1949
LOC_Os06g44010	OsWRKY28	3.7097	0.8182	0.948	-1.3869	1.2229	-1.8984	4.3396	-2.2113	-2.1322	-1.3793
LOC_Os03g32230	bsr-d1; ZOS3-12	6.1221	2.1551	1.6753	-1.112	-1.3549	-4.0854	1.9422	-2.2745	-3.5644	-1.0873
LOC_Os04g48850	OsACS2	3.9619	1.3443	0.6496	-3.5308	-1.3392	-2.1932	0.8451	-2.9741	-3.2949	-3.6599
LOC_Os11g32170		1.5952	2.0732	2.3441	0.3862	0.6695	-2.2404	1.2968	2.3036	0.0886	-1.8319
LOC_Os09g14100		2.1577	2.3398	1.9147	-0.5393	0.458	-0.6742	-1.0567	1.1318	1.9078	-1.0003
LOC_Os02g02640		1.5195	1.6044	1.1555	0.0285	0.3489	-0.0797	-0.2442	0.4534	1.6391	-0.2275
LOC_Os05g31570		4.3948	3.2984	4.2185	-0.1273	0.365	-0.1255	0.5004	-0.4252	-0.7325	-0.0983
LOC_Os11g12320		3.2207	1.592	2.7703	0.3338	1.1138	-0.8846	1.0601	-0.4016	-0.0646	0.3713
LOC_Os03g40194		2.0885	1.1023	1.578	0.1987	0.4965	0.8544	1.0377	-0.3525	-0.5318	0.068
LOC_Os11g12340	OsRSR1	2.681	1.4543	1.6456	-0.6367	0.0562	-0.5726	0.4214	-1.476	-0.6402	-0.2096
LOC_Os06g03500	Bph37	1.5276	1.268	1.134	-1.1488	-0.183	-0.4523	-0.9153	-0.9927	0.2505	0.1083
LOC_Os02g57720	OsPIP1;3	2.1219	2.064	1.5316	0.0339	-0.9566	-0.8947	0.2744	-0.389	0.4598	0.5573
LOC_Os11g45930		1.2586	1.012	1.218	-0.1468	-0.162	-0.4333	0.0102	-0.3373	0.1015	0.0017
LOC_Os12g32790		1.8052	1.9369	1.5339	0.148	0.34	-0.3752	0.3794	-0.0775	0.6808	-0.5421
LOC_Os11g45980		2.3116	2.8606	2.0794	2.9145	3.5974	0.1262	2.5974	3.8378	4.4043	0.5905
LOC_Os12g17430		2.2495	1.5356	2.2386	0.9074	2.7378	-0.8021	3.6279	1.4286	0.6058	2.3035
LOC_Os11g17330		1.8625	0.8071	2.5702	1.7217	3.6142	1.6105	3.9774	1.2419	-0.3212	2.0683
LOC_Os06g41670		0.0035	1.9885	-0.726	1.4024	2.0834	1.1616	2.083	2.5013	2.7577	1.9742
LOC_Os11g37880		-0.0012	0.2702	-1.4369	1.899	2.2075	-1.0239	1.9016	1.848	1.3864	1.3358
LOC_Os04g53050		-0.5266	1.2766	2.6819	2.8468	0.6451	0.2638	1.9436	3.4479	1.3325	1.6121
LOC_Os06g41640		0.4664	0.7308	1.7911	1.2202	1.2075	1.2275	1.4034	1.0466	1.3801	0.5835
LOC_Os11g44690		2.009	1.5379	1.287	1.0705	1.6759	0.1046	0.7787	1.314	0.3598	0.896
LOC_Os11g12050		1.4215	1.2248	1.3999	0.9463	0.7931	-0.0516	1.8656	1.3945	0.4936	0.4415

LOC_Os12g12120		1.0621	-0.0887	2.3987	1.3462	2.001	1.366	1.9394	0.1504	1.6269	1.8123
LOC_Os11g11950		0.177	-0.0337	0.1847	1.9865	1.9501	1.3158	2.0103	1.7775	1.9052	2.1946
LOC_Os11g45050		-0.0906	0.1635	0.0323	1.3649	1.2672	1.497	1.1865	0.7384	0.5668	1.5341
LOC_Os05g01140	OsJMT1	-4.251	2.0644	-3.5894	-1.7733	-1.153	-0.3508	-4.3423	0.7307	-0.1277	-0.4646
LOC_Os04g39150		-6.2474	0.4748	-2.8745	-1.488	-1.2672	-1.8819	-4.4468	0.4178	-0.0273	-1.324
LOC_Os01g28450	OsPR1b	-2.569	-3.1894	-1.8784	1.8782	0.4103	1.5161	0.5027	-0.4447	-2.7678	-3.2751
LOC_Os07g48020	Perox4	-4.3273	-1.4594	-1.4255	0.2657	0.5262	-1.5043	1.1996	0.4785	-0.5642	0.9936
LOC_Os03g36920		-1.8449	-1.393	-2.3	2.3622	0.9559	-0.1088	-0.5613	2.3346	1.3954	1.2536
LOC_Os04g08390	Bph40	-2.8048	1.2703	-2.8307	0.7215	0.137	-1.5462	-2.4981	1.4766	1.2483	-0.8018
LOC_Os02g58490	OsAGO1c	-1.8356	-1.2321	-1.1439	0.4068	-0.7631	0.0094	-0.9028	0.5015	0.7344	-0.303
LOC_Os10g04090		-2.6957	-1.1651	-1.9781	-1.0469	-0.8866	-0.2167	-2.0348	0.2643	0.3574	-0.5123
LOC_Os11g39280		-2.4754	-1.3968	-2.2923	-0.357	-1.2445	-0.2769	-0.998	-0.0195	0.5501	-1.0456
LOC_Os11g30060		-3.9156	-1.6799	-4.8419	5.8175	1.1582	1.6569	1.7997	5.8305	5.2439	3.0626
LOC_Os03g63240		-4.6272	1.5095	-2.3512	3.4646	2.9732	0.9981	2.2794	3.1816	3.3442	4.2558
LOC_Os12g11930		-1.7147	-1.9645	-1.5875	3.972	2.8508	2.378	2.7183	3.8027	3.4394	2.5465
LOC_Os12g10870		-0.612	0.2568	-0.1824	6.5847	6.7623	6.1674	6.7061	6.0332	5.4152	5.3463
LOC_Os12g11860		1.8083	0.1971	0.8463	7.6367	7.6215	5.1893	7.9751	5.8773	7.2298	8.0244
LOC_Os12g11680		0.4401	0.4996	0.8306	6.9993	6.8344	6.5209	7.3262	6.5152	6.9686	7.2723
LOC_Os11g11810	RGA5	0.0357	-0.2446	0.2108	7.7418	7.8081	7.6888	7.6922	7.7285	7.873	7.691
LOC_Os11g44960		1.2039	2.4188	1.4243	5.2452	2.9167	4.3807	4.1847	5.1518	4.824	1.8113
LOC_Os11g11770		0.3173	-0.2071	0.2119	4.5139	4.1224	3.266	4.3221	4.4308	3.8725	3.7848
LOC_Os11g45180		0.5342	0.4286	0.6771	4.8407	4.1713	5.0359	5.1678	4.2584	3.3372	3.2896
